# Overdiagnosis of Attention-Deficit/Hyperactivity Disorder in Children and Adolescents: A Systematic Scoping Review

**DOI:** 10.1001/jamanetworkopen.2021.5335

**Published:** 2021-04-12

**Authors:** Luise Kazda, Katy Bell, Rae Thomas, Kevin McGeechan, Rebecca Sims, Alexandra Barratt

**Affiliations:** 1 Sydney School of Public Health, Faculty of Medicine and Health, The University of Sydney, Sydney, New South Wales, Australia; 2 Institute for Evidence-Based Healthcare, Bond University, Gold Coast, Queensland, Australia

## Abstract

**Importance:**

Reported increases in attention-deficit/hyperactivity disorder (ADHD) diagnoses are accompanied by growing debate about the underlying factors. Although overdiagnosis is often suggested, no comprehensive evaluation of evidence for or against overdiagnosis has ever been undertaken and is urgently needed to enable evidence-based, patient-centered diagnosis and treatment of ADHD in contemporary health services.

**Objective:**

To systematically identify, appraise, and synthesize the evidence on overdiagnosis of ADHD in children and adolescents using a published 5-question framework for detecting overdiagnosis in noncancer conditions.

**Evidence Review:**

This systematic scoping review adhered to the Preferred Reporting Items for Systematic Reviews and Meta-analyses (PRISMA) Extension for Scoping Reviews and Joanna Briggs Methodology, including the PRISMA-ScR Checklist. MEDLINE, Embase, PsychINFO, and the Cochrane Library databases were searched for studies published in English between January 1, 1979, and August 21, 2020. Studies of children and adolescents (aged ≤18 years) with ADHD that focused on overdiagnosis plus studies that could be mapped to 1 or more framework question were included. Two researchers independently reviewed all abstracts and full-text articles, and all included studies were assessed for quality.

**Findings:**

Of the 12 267 potentially relevant studies retrieved, 334 (2.7%) were included. Of the 334 studies, 61 (18.3%) were secondary and 273 (81.7%) were primary research articles. Substantial evidence of a reservoir of ADHD was found in 104 studies, providing a potential for diagnoses to increase (question 1). Evidence that actual ADHD diagnosis had increased was found in 45 studies (question 2). Twenty-five studies showed that these additional cases may be on the milder end of the ADHD spectrum (question 3), and 83 studies showed that pharmacological treatment of ADHD was increasing (question 4). A total of 151 studies reported on outcomes of diagnosis and pharmacological treatment (question 5). However, only 5 studies evaluated the critical issue of benefits and harms among the additional, milder cases. These studies supported a hypothesis of diminishing returns in which the harms may outweigh the benefits for youths with milder symptoms.

**Conclusions and Relevance:**

This review found evidence of ADHD overdiagnosis and overtreatment in children and adolescents. Evidence gaps remain and future research is needed, in particular research on the long-term benefits and harms of diagnosing and treating ADHD in youths with milder symptoms; therefore, practitioners should be mindful of these knowledge gaps, especially when identifying these individuals and to ensure safe and equitable practice and policy.

## Key Points

**Question:**

Is attention-deficit/hyperactivity disorder (ADHD) overdiagnosed in children and adolescents?

**Findings:**

In this systematic scoping review of 334 published studies in children and adolescents, convincing evidence was found that ADHD is overdiagnosed in children and adolescents. For individuals with milder symptoms in particular, the harms associated with an ADHD diagnosis may often outweigh the benefits.

**Meaning:**

This finding suggests that high-quality studies on the long-term benefits and harms of diagnosing and treating ADHD for youths with milder or borderline symptoms are needed to inform safe and equitable practice and policy.

## Teaser

This systematic scoping review evaluates the multidecade pattern of diagnosis in attention-deficit/hyperactivity disorder in young people using a framework for identifying overdiagnosis for noncancer conditions.

## Introduction

Public debate over the appropriateness of attention-deficit/hyperactivity disorder (ADHD) diagnosis has grown along with diagnosis rates.^1,2,3,4,5,6^ Disagreement continues about how much of the increased diagnoses can be attributed to true increases in frequency, improved detection, or diagnostic inflation because of misdiagnosis and/or overdiagnosis.^7,8,9,10,11,12^ The concept of overdiagnosis is well established in cancer,^13,14^ but it also occurs in noncancer conditions.^15,16,17^ Methods to investigate overdiagnosis in noncancer conditions were published recently^18^ but have not been applied to ADHD yet.

Overdiagnosis of ADHD could happen because of diagnostic inflation^10,19^ by widening the definition to include ambiguous or mild symptoms, by explicitly changing the diagnostic definition,^10,20^ or by implicitly medicalizing behavioral patterns that previously would not have been considered abnormal^1,21^ (eg, those behaviors that are typical of children who are relatively young for their school year^22^). However, for increased detection to represent current overdiagnosis rather than previous underdiagnosis of ADHD, we also need evidence that these additional cases do not derive a net benefit from diagnosis (ie, these children’s overall health is not improved because the harms of diagnosis and treatment outweigh the benefits^23,24,25^). Although the benefits of appropriate diagnosis and treatment of ADHD may be well known,^12^ harms are less well appreciated. Physical and psychosocial harms (and financial costs) may be experienced directly by the young patients and their family, but economic and opportunity costs are experienced by the wider society.^7^

In this study, we systematically reviewed the literature to identify, appraise, and synthesize the evidence on overdiagnosis of ADHD in children and adolescents. Moreover, we aimed to highlight any existing evidence gaps. We used a 5-question framework for detecting overdiagnosis in noncancer conditions.

## Methods

Because of the broad research question, we conducted a systematic scoping review that adhered to the Preferred Reporting Items for Systematic Reviews and Meta-analyses (PRISMA) Extension for Scoping Reviews^26^ and Joanna Briggs Methodology,^27^ including the provision of a PRISMA-ScR Checklist. A summary of the methods is given here, and the details are published elsewhere.^28^

Overdiagnosis is defined here as occurring when a person is clinically diagnosed with a condition, but the net effect of the diagnosis is unfavorable.^18,23,29^ Misdiagnosis (when a child is incorrectly labeled with an ADHD diagnosis instead of an alternative condition^10,23^) and false-positive diagnosis (when a subsequent clinical encounter reveals a wrong initial diagnosis^23^) are not the focus of this article.

**Figure 1.**
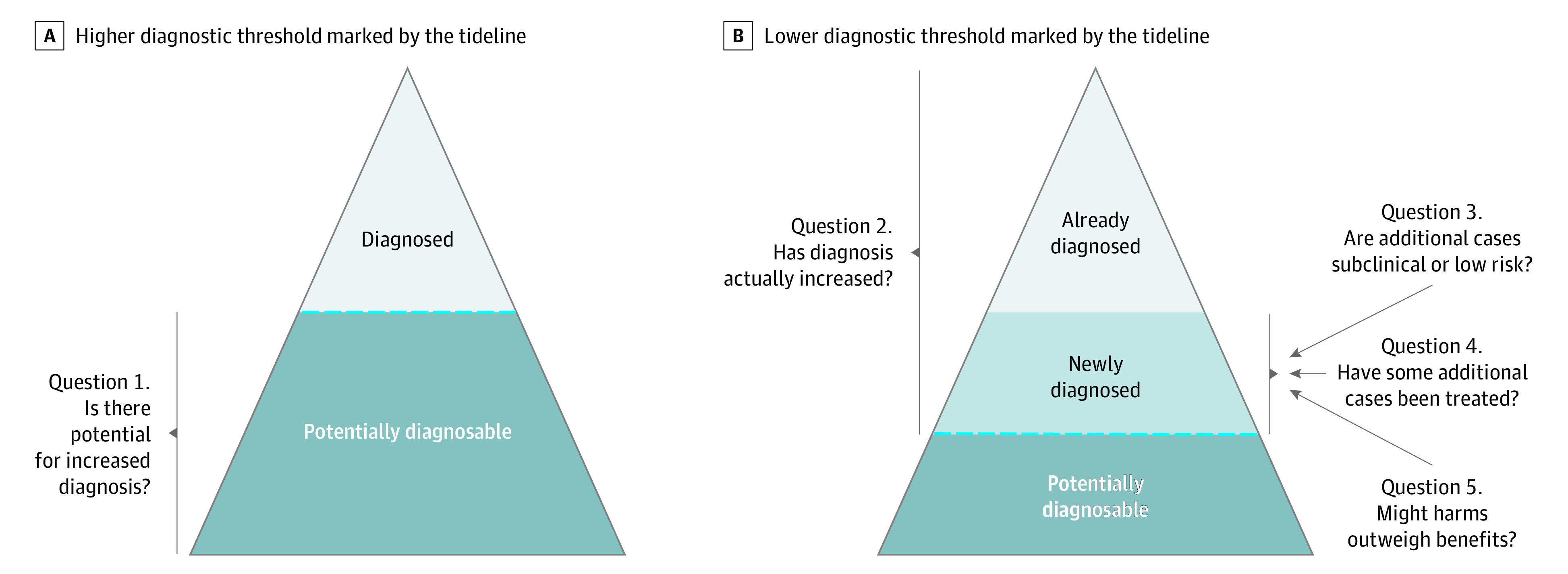
Five-Question Framework for Identifying Potential Attention-Deficit/Hyperactivity Disorder (ADHD) Overdiagnosis The model of an iceberg illustrates how the framework relates to subsets of the population who may be diagnosed with ADHD. Question 1 concerns youths who may be diagnosable with ADHD, question 2 concerns youths who are diagnosed with ADHD according to newer criteria or those who were already diagnosed with ADHD using older thresholds, and questions 3 to 5 concern youths who are newly diagnosed vs those who are already diagnosed (adapted from Bell et al^18^).

The conceptual basis for this review was a previously published framework for identifying characteristics that are consistent with overdiagnosis and subsequent overtreatment in noncancer conditions.^18^ All included data were mapped to these 5 questions: (1) Is there potential for increased diagnosis? (2) Has diagnosis actually increased? (3) Are additional cases subclinical or low risk? (4) Have some additional cases been treated? (5) Might harms outweigh benefits of diagnosis (5a) and treatment (5b) (Figure 1)?

Peer-reviewed primary and secondary studies in children and adolescents that were published in English between January 1, 1979, and August 21, 2020, were eligible for inclusion. Studies with mixed-age populations were included if it was possible to extract data from them separately or if most participants were aged 18 years or younger. Given the overwhelming amount of evidence on pharmacological ADHD treatment outcomes, we included systematic reviews and cohort studies only for question 5b. Studies needed to have a clear emphasis on ADHD.

Searches were performed on August 21, 2020, in MEDLINE, Embase, PsychINFO, and the Cochrane Library (eAppendix 1 in Supplement 1). These database searches were supplemented by backward citation searches of all included articles and forward citation searches on key research.

After the removal of duplicates and a pilot phase, 2 of us (L.K. and R.S.) independently screened abstracts using the web-based text mining tool Abstrackr (Brown University).^30,31^ Subsequently, the full texts of all potential articles were independently reviewed by ^1^ of us (L.K.) and another researcher. Any discrepancies were resolved through discussion.

### Data Extraction and Quality Assessment

Data were extracted into a standardized template, which was developed through an iterative process (eAppendix 2 in Supplement 1). Data from qualitative studies were mapped using NVivo, version 12 Plus (QSR International). Uncertainties were resolved by team discussion. A quality assessment of included studies was conducted by one of us (L.K.) using critical appraisal checklists developed by the Joanna Briggs Institute.^32^

### Data Synthesis and Analysis

Data were considered in the context of the 5 questions and then stratified into themes and subthemes for analysis. Each study could contribute data to more than 1 question.

To investigate whether a reservoir of potentially diagnosable ADHD existed (question 1), we looked for prevalence variations and other indicators, such as evidence of a spectrum of symptoms. For example, the lack of biological explanations for large prevalence variations among populations or among diagnostic standards would indicate a reservoir of potentially diagnosable disease. To analyze the data on ADHD diagnosis and treatment patterns (questions 2 and 4), we included any studies that provided time-trend data on clinical diagnosis or medication rates.

The question of whether additional diagnoses were predominantly mild cases (question 3) was central to ascertaining whether extra detection represented a net benefit or harm. However, severity of ADHD was not consistently defined or assessed, relying heavily on subjective interpretations.^25^ We grouped the evidence for this question into 2 categories: studies that reported ADHD severity and studies that reported degree of impairment as a proxy.

We divided the evidence on benefits and harms (question 5) into outcomes of the diagnosis and outcomes of any subsequent treatment, with a focus on the ratio of benefits to harms specifically for youths with milder ADHD-related behaviors.^18^ In addition, we considered the evidence on benefits and harms across the wider ADHD spectrum.

## Results

Of the 12 267 records retrieved, 334 studies (2.7%) were included. eAppendix 3 in Supplement 1 outlines the selection process in a PRISMA flow diagram.^26^

Of the 334 included studies, 61 (18.3%) were secondary and 273 (81.7%) were primary research articles. Most studies were published within the past 10 years (n = 217 [65.0%]) and were most commonly from North America (n = 128 [38.3%]), Europe (n = 93 [27.8%]), or Oceania/Asia (n = 35 [10.5%]) (Table 1; eAppendix 4 in Supplement 1 and Supplement 2).

**Table 1.** General Characteristics of Included Sources of Evidence

Characteristic	No. (%)
Total No.	334
Year of publication	
1979-1990	5 (1.5)
1991-2000	23 (6.9)
2001-2010	89 (26.6)
2011-2020	217 (65.0)
Region	
North America	128 (38.3)
Rest of Europe	35 (10.5)
Scandinavia	33 (9.9)
United Kingdom/Ireland	25 (7.5)
Asia	18 (5.4)
Australia/New Zealand	17 (5.1)
Middle East	10 (3.0)
Not specified/various regions	68 (20.4)
Study design	
Cohort	90 (26.9)
Cross-sectional	6 (1.8)
Diagnostic test accuracy	9 (2.7)
Prevalence	142 (42.5)
Qualitative	12 (3.6)
Randomized clinical trial	14 (4.2)
Systematic review	44 (13.9)
Other review	17 (5.1)

**Figure 2.**
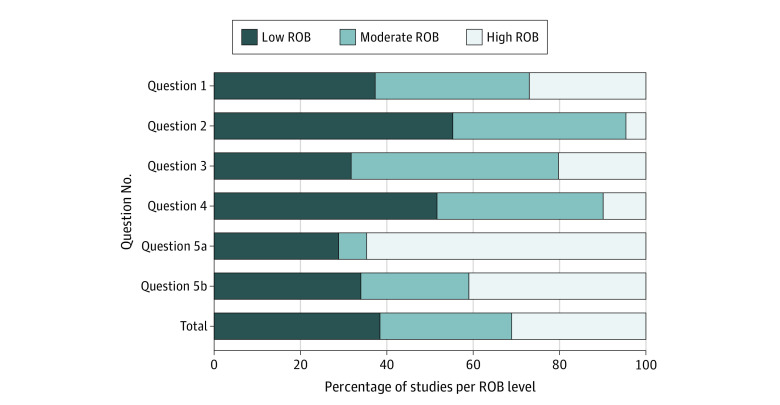
Risk of Bias (ROB) Assessment The full results from the critical appraisals are found in eAppendix 5 in Supplement 1.

The quality of included studies varied; approximately one-third of the studies were classified as having low (n = 129), moderate (n = 102), or high (n = 103) risk of bias. Studies that provided evidence for question 5 were more likely to be at high risk of bias (n = 69 [45.7%]) (Figure 2 and eAppendix 5 in Supplement 1).

Table 2 maps the evidence against the framework.^1,2,3,5,6,10,11,20,21,22,33,34,35,36,37,38,39,40,41,42,43,44,45,46,47,48,49,50,51,52,53,54,55,56,57,58,59,60,61,62,63,64,65,66,67,68,69,70,71,72,73,74,75,76,77,78,79,80,81,82,83,84,85,86,87,88,89,90,91,92,93,94,95,96,97,98,99,100,101,102,103,104,105,106,107,108,109,110,111,112,113,114,115,116,117,118,119,120,121,122,123,124,125,126,127,128,129,130,131,132,133,134,135,136,137,138,139,140,141,142,143,144,145,146,147,148,149,150,151,152,153,154,155,156,157,158,159,160,161,162,163,164,165,166,167,168,169,170,171,172,173,174,175,176,177,178,179,180,181,182,183,184,185,186,187,188,189,190,191,192,193,194,195,196,197,198,199,200,201,202,203,204,205,206,207,208,209,210,211,212,213,214,215,216,217,218,219,220,221,222,223,224,225,226,227,228,229,230,231,232,233,234,235,236,237,238,239,240,241,242,243,244,245,246,247,248,249,250,251,252,253,254,255,256,257,258,259,260,261,262,263,264,265,266,267,268,269,270,271,272,273,274,275,276,277,278,279,280,281,282,283,284,285,286,287,288,289,290,291,292,293,294,295,296,297,298,299,300,301,302,303,304,305,306,307,308,309,310,311,312,313,314,315,316,317,318,319,320,321,322,323,324,325,326,327,328,329,330,331,332,333,334,335,336,337,338,339,340,341,342,343,344,345,346,347,348,349,350,351,352,353,354,355,356^ A summary of the findings is described here. The full results are provided in eAppendix 6 in Supplement 1.

**Table 2.** Main Results Mapped to the 5-Question Framework^a^

Type of evidence (No. of studies)	Theme (No. of studies)	Subtheme (No. of studies)	Main outcomes (No. of studies)
**Question 1. Is there potential for increased diagnosis (n = 104 studies)**			
Prevalence variations (68)	By subpopulation (48)	Sex (25)	Lower diagnosis in girls than boys (23)^5,6,33,34,35,36,37,38,39,40,41,42,43,44,45,46,47,48,49,50,51,52,53^ Symptomatic girls less likely to be diagnosed (2)^54,55^
		SES or insurance status (21)	Higher diagnosis in lower SES (13)^5,6,35,36,39,43,45,46,49,53,56,57,58^ Higher diagnosis in higher SES (2)^59,60^ Higher diagnosis in public vs private health insurance (5)^5,41,53,58,61^ Lower diagnosis in no vs any health insurance (7)^5,53,54,58,62,63,64^ No association of insurance status with diagnosis in hypothetical scenario (1)^55^
		Race/ethnicity (21)	Lower diagnosis in Black and Hispanic vs White youths (14)^6,35,36,37,41,42,48,49,53,56,62,63,65,66^ Lower diagnosis in White vs Black youths (4)^39,51,52,67^ Lower diagnosis in non-English-speaking and migrant youths (3)^35,36,45^ No association of race/ethnicity with diagnosis in hypothetical scenario (1)^55^
		Relative age (12)	Youngest children in class more likely to be diagnosed (11)^22,68,69,70,71,72,73,74,75,76,77^ No difference in diagnosis probability by relative age (1)^78^
		Location/region (8)	Large variations in diagnosis by region (8)^6,33,35,36,39,46,56,59^
		Other (1)	Higher diagnosis and reported symptoms in larger classes (1)^42^
	By diagnosis (20)	Diagnostic criteria (20)	Broadening of criteria associated with increases in potential cases in comparisons between any *DSM* version and/or *ICD-10* (18)^10,20,52,79,80,81,82,83,84,85,86,87,88,89,90,91,92,93^ Broadening of age of onset associated with minimal increases in potential cases (2)^94,95^
Reservoir (44) attributed to	Medicalization (3)	Behavioral problems (3)	Society’s decreasing tolerance for different behavior associated with increased range of behavior diagnosed as abnormal (2)^57,96^ Mental health professionals from China and Indonesia rating same attention difficulties higher than mental health professionals from US and Japan (1)^97^
	Phenotype changes (5)	Trends over time (5)	No increase in clinically significant symptoms (4)^1,21,98,99^ Increase in youths with clinically significant symptoms (1)^100^ Increase in subthreshold symptoms (1)^1^
	Diagnostic inaccuracy (16) associated with	Over- and underdiagnosis (16)	Potential over- and underdiagnosis occurring (6)^11,64,65,101,102,103^ Potential underdiagnosis attributed to false-negatives (5)^1,21,38,104,105^ Potential overdiagnosis attributed to false-positives (5)^54,93,100,106,107^
	Spectrum of disorder (22) indicated by subtheme	Implications of extent of symptoms over time (13)	Continuous association between increasing symptoms and increased risk of various later adverse outcomes (13)^108,109,110,111,112,113,114,115,116,117,118,119,120^
		Subthreshold prevalence (8)	Many youths with subthreshold symptoms (6)^108,109,110,114,115,121^ Percentage of youths with symptoms decreases with age (2)^122,123^
		Verification of dimensional structure (7)	Inattention, hyperactivity, and impulsivity problems exist on a spectrum with ADHD at the end (7)^119,124,125,126,127,128,129^
**Question 2. Has diagnosis actually increased? (n = 45 studies)**			
Diagnosis (45)	Trend over time (45) indicated by subtheme	Change in prevalence (30)	Increasing trend (27)^3,5,21,34,39,41,44,47,50,61,66,74,130,131,132,133,134,135,136,137,138,139,140,141,142,143,144^ until early 2000s (3)^33,60,145^
		Change in incidence (12)	Increasing trend (11)^2,3,40,43,44,140,142,146,147,148,149^ until 2007 (1)^60^
		Change in lifetime prevalence (12)	Increasing trend (11)^1,2,5,6,21,36,37,40,53,56,150^ Stagnant in adolescents and increasing in children (1)^58^
**Question 3. Are additional cases subclinical or low risk? (n = 25 studies)**			
Severity of disorder (17) indicated by theme	Impairment levels (11) indicated by subtheme	Change in adverse outcomes over time (2)	Decreasing problems with increasing ADHD group (1)^100^ Reduction in hospital visits in medicated children decreasing with increasing group of youths with ADHD (1)^150^^,^^b^
		Difference in adverse outcomes by diagnostic criteria (9)	Broadening of diagnostic criteria and increasing of ADHD group associated with less impairment (5)^79,85,86,88,93^ Reduction in symptoms and impairments in medicated children decreasing with broadening of ADHD criteria (1)^151^^,^^b^ Sample size too small to detect differences (4)^84,87,94^
	Symptom severity (6) indicated by subtheme	Trend over time (3)	Severity proportions stable (1)^132^ Larger increase in moderate/severe cases (2)^5,138^
		Severity proportions (2)	Proportion of youths with severe ADHD being low (2)^36,152^
		Diagnostic criteria (1)	No difference in severity between late and early-onset cases (1)^92^
Level of symptoms (8)	Clinically significant symptom prevalence (8) by	Change over time (8)	Prevalence estimates of symptomatic ADHD remaining steady or slightly decreasing (6)^1,21,90,91,98,99^ Relative age effect increasing in later birth cohorts (2)^70,75^
**Question 4. Have some additional cases been treated? (n = 83 studies)**			
Medication (83)	Trend over time (83) indicated by subtheme	Prevalent treatment rate (58)	Increasing trend (55)^2,5,34,39,40,50,58,74,133,136,141,142,143,150,153,154,155,156,157,158,159,160,161,162,163,164,165,166,167,168,169,170,171,172,173,174,175,176,177,178,179,180,181,182,183,184,185,186,187,188,189,190,191,192,193^ Decreases in those younger than 6 years and increases in those older than 6 years (2)^194,195^ Mostly decreasing trend (2)^134,163^
		Incident treatment rate (9)	Increasing trend (5)^142,166,175,196,197^ Stable or variable rate (4)^172,198,199,200^
		Medication use (11)	Increasing trend (10)^50,201,202,203,204,205,206,207,208,209^ until 2010 (1)^210^
		Consultations in which ADHD medication is prescribed (10)	Increasing trend (9)^41,47,132,135,139,144,145,211,212^ Increasing trend until 2004 in those younger than 6 years (1)^213^
**Question 5a. Might harms outweigh benefits of diagnosis? (n = 31 studies)**			
Potential benefits of diagnosis (15)	Empowered (14) through	Explanation for problems (11)	Increased legitimacy and validation (5)^57,214,215,216,217^ Increased understanding, sympathy, and reassurance (8)^215,216,217,218,219,220,221,222^ Decreased guilt, blame, failure, and anger (6)^57,214,215,219,222,223^
		Increased control (6)	Decreased uncertainty; feeling of identity and belonging (3)^215,218,223^ Increased self-esteem and confidence (3)^215,218,220^ Expectation of solution (4)^214,215,218,222^
	Enabled (10) to	Support (10)	Increased ability to seek, receive, and accept support (10)^57,96,214,215,216,217,219,220,222,223^
Potential harms of diagnosis (29)	Disempowered (22) through	Excuse for problems (15)	Decreased responsibility for behaviors and problems (6)^96,214,216,220,224,225^ Increased deflection from underlying problems (3)^57,214,215^ No meaningful benefit from diagnosis or no change (10)^96,103,216,218,220,222,226,227,228,229^
		Loss of control (15)	Associated with control and manipulation by others (4)^96,214,220,223^ Increased passiveness and hopelessness (3)^215,216,222^ Self-fulfilling prophecy: perceived inability to change or achieve (by self or others) associated with exclusion and fewer opportunities (9)^64,215,217,230,231,232,233,234,235^^,^^b^
	Stigmatization (14) through	Permanent label and identity (14)	Enhanced prejudice, stereotypes, and judgment (14)^215,216,217,218,220,222,225,230,232,236,237,238,239,240^ Increased feelings of isolation, exclusion, and shame (3)^216,220,225^
**Question 5b. Might harms outweigh benefits of treatment (n = 120 studies)**			
Outcomes of pharmacological treatment (120)	Academic (19)	Cognitive and motor functioning (4)	Improvements in commission errors only; all others unchanged (1)^241^ Favorable outcomes in several aspects of cognition (1)^242^ and motor skills (1)^243^ No change after washout period (1)^228^
		Academic performance (15)	Worse educational outcomes in treated vs rest of the population (1)^244^ No treatment effect (3)^245,246,247^ Some small favorable outcomes in treated vs untreated youths (6)^248,249,250,251,252,253^ Substantial improvement in treated vs untreated or less treated youths (2)^254,255^ Decrease in academic outcomes after increased medication treatment (1)^256^ Potential harmful outcome, especially in youths with less severe symptoms (1)^64^^,^^b^ Medication treatment only beneficial for youths with more severe symptoms (1)^233^^,^^b^
	Accidents (12)	ED use and hospital admissions (5)	Fewer hospital contacts in treated vs untreated youths, but outcomes smaller in later, larger diagnosed and treated cohorts (1)^150^^,^^b^ No change in hospital contacts in treated vs untreated periods and youths (2)^257,258^ Fewer hospital contacts during treated vs untreated periods (2)^257,259^ Worse health outcomes in treated vs rest of the population (1)^244^
		Injuries and poisoning (8)	Lower risk of injuries in treated vs untreated periods (3)^259,260,261^ and youths (2)^262,263^ No change in injuries (1)^264^ or MVAs (1)^265^ during treated vs untreated periods Increase in unintentional poisonings with ADHD medication (1)^266^
	Cardiovascular (8)	Blood pressure and heart rate (2)	No association of treatment with blood pressure (2)^267,268^ but with heart rate (1)^268^
		Safety (6)	No association of treatment with severe cardiovascular events (3)^269,270,271^ Increased risk of arrhythmia (1)^272^ or any serious cardiac event (1)^273^ Not enough statistical power to detect small differences (3)^269,270,274^
	Efficacy (30)	Symptom reduction (30)	Substantial short-term symptom reduction for many (24)^196,241,247,275,276,277,278,279,280,281,282,283,284,285,286,287,288,289,290,291,292,293,294,295^ No symptom improvement after 48-hour washout period (1)^296^ No long-term difference in treated vs untreated youths (3)^245,297,298^ Individuals with more severe symptoms at baseline showed greatest treatment response in the long term (5 years) (1)^299^^,^^b^
	Physical (14)	Activity (2)	Lower levels of physical activity in treated vs untreated periods (1)^300^ and youths (1)^301^
		Height (12) and weight (5)	Growth delay (2)^302,303^ and decreased growth (6)^297,304,305,306,307,308^ or weight (3)^304,305,308^ No change observed and substantial heterogeneity (5)^267,301,303,309,310^
	Psychological (20)	Other (2)	Increased risk of psychosis (1)^311^ and tics (1)^312^
		Substance abuse (12)	No association of treatment with later substance abuse (4),^313,314,315,316^ reduced risk (5),^317,318,319,320,321^ and increased risk of stimulant abuse (1)^322^ Prescription stimulant misuse or diversion in youths with or without ADHD (2)^323,324^
		Suicidal behavior (6)	No association of treatment with risk of suicidal behavior (2),^325,326^ reduced risk (3),^327,328,329^ and increased risk (1)^330^
	Social and emotional (11)	Emotional (3)	Increased risk of irritability with amphetamine treatment (1)^331^ Mixed outcomes for various emotional behaviors with stimulant treatment (1)^332^ Decrease in happiness after increased medication treatment (1)^256^
		Criminal behavior (3)	Reduced risk of conviction and incarceration in treated vs untreated periods (1)^333^ No change in risk of receiving driving citation for treated vs untreated periods (1)^265^ Reduced risk of being charged with a crime in treated vs untreated youths, but effects were smaller in later, larger diagnosed and treated cohorts (1)^150^^,^^b^
		Social impairment (2)	Little change in social impairment in treated vs untreated period (1)^246^ Potentially relevant improvements in some domains in treatment vs placebo group (1)^334^
		Quality of life (4)	Small short-term improvements in quality of life (3)^334,335,336^; no impact (1)^337^
	Tolerability (29)	Adverse events (25)	Low occurrence of mild AEs (2)^196,277^ Relatively common mild or moderate AEs (16)^276,278,280,281,286,289,291,292,294,295,299,338,339,340,341,342^ Young children more vulnerable to AEs (2)^295,343^ Reporting of AEs unsatisfactory (6)^282,291,294,344,345,346^ Serious AEs rare but difficult to determine from reported data (5)^287,338,340,345,346^
		Discontinuation (6)	Moderate to high discontinuation rates (20%-44%) (4)^290,341,347,348^ Discontinuation similar to placebo group or low-quality evidence (2)^275,285^
	Various (8)	Mixed (8)	50% of various outcomes reported some benefits of treatment (1)^349^ Reduced risk of various outcomes (2)^350,351^ Lack of methodologically sound research on which to base decisions (1)^352^ Longer treatment duration indicated better outcomes for various domains (1)^353^ No long-term treatment effect for various functioning outcomes (3)^354,355,356^

Abbreviations: ADHD, attention-deficit/hyperactivity disorder; AE, adverse event; *DSM*, *Diagnostic and Statistical Manual of Mental Disorders*; ED, emergency department; *ICD-10*, *International Statistical Classification of Diseases and Related Health Problems, Tenth Revision*; MVA, motor vehicle accident; SES, socioeconomic status.

^a^ Full main results are presented in eAppendix 6 in Supplement 1.

^b^ Items refer specifically to the benefits and harms for young people with less severe ADHD behaviors.

### Large Reservoir of Potentially Diagnosable ADHD

A total of 104 studies were included to answer question 1. Large variations in ADHD diagnosis were found between subpopulations in 48 studies. Twenty-five studies provided evidence of variation between the sexes, showing lower diagnosis of ADHD in girls than in boys. Although biological reasons may exist,^38,45^ equally symptomatic girls were less likely to be diagnosed than boys in 2 studies.^54,55^ Eight studies showed decreasing ratios over time, which were indicative of a reservoir of diagnosable ADHD in girls.^5,6,34,37,40,41,44,47^

Of the 12 included studies that focused on relative age, 11 studies showed that the youngest children in class were more likely to be diagnosed with ADHD than the oldest children.^22,68,69,70,71,72,73,74,75,76,77^ One study did not confirm this finding.^78^ It was conducted in a low-prevalence setting in which only specialists diagnosed ADHD, suggesting that variation (and a potential reservoir) is much smaller where stricter adherence to diagnostic criteria may occur.

Youths from various migrant backgrounds were traditionally less likely to be diagnosed with ADHD in 15 studies.^6,35,36,37,41,42,45,48,49,53,56,62,63,65,66^ However, there is evidence that diagnosis rates increased rapidly (especially in young Black youths, often overtaking the rates in White youths).^6,37,39,41,51,52,56,66,67^ Twenty-one studies^5,6,35,36,39,41,43,45,46,49,53,54,55,56,57,58,59,60,61,62,63,64^ on diagnostic variation by socioeconomic or health insurance status and 8 studies^6,33,35,36,39,46,56,59^ on regional variation all demonstrated substantial differences.

Eighteen of 20 studies that compared diagnostic prevalence between 2 or more diagnostic criteria described a concurrent increase in potential cases with the broadening of criteria.^10,20,52,79,80,81,82,83,84,85,86,87,88,89,90,91,92,93^ Twenty-two studies reported a spectrum of ADHD-related behaviors showing that problems existed on a continuum, with ADHD on the extreme end in 7 studies^119,124,125,126,127,128,129^ and subthreshold behaviors on the other end displayed by a considerable proportion of young people in 8 studies,^108,109,110,114,115,121,122,123^ indicating a large reservoir of potentially diagnosable ADHD. This continuum was also described by a higher risk of adverse outcomes with increasing ADHD symptoms from subthreshold symptom levels to severe behaviors in 13 studies.^108,109,110,111,112,113,114,115,116,117,118,119,120^

Evidence of expanding reservoirs attributed to the medicalization of behavior was found in 3 studies.^57,96,97^ Four of 5 phenotype change studies reported stable or declining ADHD symptoms in the population, making it unlikely that the expansion was associated with an actual increase in ADHD symptoms over time.^1,21,98,99^

Sixteen studies that investigated diagnostic inaccuracies as a reason for variation reported potential underdiagnosis because of false-negative diagnosis^1,21,38,104,105^ and potential overdiagnosis because of false-positive diagnosis,^54,93,100,106,107^ often occurring simultaneously.^11,64,65,101,102,103^

### Consistent Increases in ADHD Diagnosis Between 1989 and 2017

Of the 45 studies included to answer question 2, 30 studies estimated change in diagnostic prevalence of ADHD over time, with 27 documenting increased trends^3,5,21,34,39,41,44,47,50,61,66,74,130,131,132,133,134,135,136,137,138,139,140,141,142,143,144^ and 3 observing a plateau in the early 2000s.^33,60,145^ Similar evidence came from studies that measured trends in annual diagnostic incidence^2,3,40,43,44,60,140,142,146,147,148,149^ or lifetime diagnostic prevalence,^1,2,5,6,21,36,37,40,53,56,58,150^ with nearly all of these studies (96.0%) confirming continuously increasing ADHD diagnoses.

### Many Additional Cases On the Milder End of the ADHD Spectrum

Twenty-five studies were included to answer question 3. Five studies reported that only a small proportion of all diagnosed youths displayed severe ADHD behaviors.^5,36,132,138,152^

Eleven studies used changes in impairment as a proxy for severity. Eight of these studies confirmed that impairment levels, adverse outcomes, and benefits of medication substantially decreased with the expansion of the group of diagnosed youths.^79,85,86,88,93,100,150,151^ Six studies that confirmed stable or declining ADHD behaviors in youths over several decades supported this finding.^1,21,90,91,98,99^ Correspondingly, 2 studies^70,75^ on the relative age effect on ADHD diagnosis reported that the proportion of youngest children in class who received a diagnosis compared with older children had increased in more recent birth cohorts. In contrast, 3 studies^5,132,138^ that reported time trends of parent- or clinician-perceived severity of the disorder showed larger relative increases in more severe cases (which could be associated with a growing tendency to report the same behaviors as more severe).

### Substantial Increases in Pharmacological Treatment for ADHD Between 1971 and 2018

Of the 83 studies included to answer question 4, 64 showed an increasing percentage of youths being pharmacologically treated for ADHD. Three studies^194,195,213^ identified a plateau or decrease in this trend for preschool-aged children, with 2 studies^134,163^ indicating a general declining trend in youths in Germany who received treatment. Annual incident treatment rates were reported to be increasing in 5 studies^142,166,175,196,197^ and to be stable or without a clear trend in 4 studies,^172,198,199,200^ whereas 10 of 11 studies^50,201,202,203,204,205,206,207,208,209^ reported increasing trends in medication dispensing or sales.

### Benefits May Be Outweighed by Harms in Youth With Milder ADHD Symptoms 

#### 

##### Diagnosis

A total of 31 studies reported the consequences of diagnosis (question 5a). We focused on the benefits and harms for milder cases. Only 2 studies^64,233^ provided information on this group and both reported harms and found that the diagnostic label could have adverse social, psychological, and academic effects when compared with undiagnosed youths with similar behaviors.

Regarding the general benefits of a diagnosis (across the full spectrum of ADHD cases), 2 main themes emerged. First, in 14 studies, an ADHD diagnosis was shown to create a sense of empowerment for those involved. It provided a biomedical explanation for experienced problems, supporting a sense of legitimacy^57,214,215,216,217^ accompanied by understanding and sympathy^215,216,217,218,219,220,221,222^ as well as decreased guilt, blame, and anger.^57,214,215,219,222,223^ Subsequently, this explanation could increase perceived control, with expectations of solutions,^214,215,218,222^ enhanced confidence,^215,218,220^ and a sense of belonging.^215,218,223^ Second, enablement was often experienced^57,96,214,215,216,217,219,220,222,223^ and was characterized by increased support accompanying a diagnosis of ADHD and by an enhanced ability to seek and accept help.^215,216,222,223^

Two themes related to potential harms also emerged. First, in 22 studies, a biomedical view of difficulties was shown to be associated with disempowerment. By providing an excuse for problems, a decrease in responsibility by all involved can occur,^96,214,216,220,224,225^ often followed by inaction and stagnation.^96,103,216,218,220,222,226,227,228,229^ This view can also deflect from other underlying individual, social, or systemic problems,^57,214,215^ which can prompt a self-fulfilling prophecy, wherein the perceived inability to change reduces opportunities^64,215,217,230,231,232,233,234,235^ as well as promotes hopelessness and passiveness.^215,216,222^ This loss of control may be especially high when the diagnosis is used as a step toward coercing young people into correcting arguably problematic behaviors.^96,214,220,223^ Second, 14 studies reported on stigmatization. The diagnosis can create an identity that enhances prejudice and judgment,^215,216,217,218,220,222,225,230,232,236,237,238,239,240^ which are associated with even greater feelings of isolation, exclusion, and shame.^216,220,225^

##### Treatment

A total of 120 studies reported on the consequences of pharmacological treatment. Forty studies reported on the direct outcomes of pharmacological treatment of ADHD, including 2 studies^151,299^ on treatment efficacy stratified by severity of ADHD behaviors. These studies confirmed substantially greater treatment response in youths with more severe symptoms at baseline and diminished benefits in milder cases. None of the 29 studies that reported on direct harms of treatment differentiated between case severity.^196,275,276,277,278,280,281,282,285,286,287,289,290,291,292,294,295,299,338,339,340,341,342,343,344,345,346,347,348^

Of the 85 studies on indirect outcomes of treatment, 3 studies reported on youths with less severe ADHD. Two of these articles^64,233^ suggested that treatment was only beneficial to academic outcomes in youths with severe symptoms, with 1 study^64^ reporting a potentially harmful outcome in milder cases. Another study^150^ found that the benefits of medication in reducing hospital contacts and criminal behavior were smaller in later birth cohorts for whom treatment prevalence was higher (thus likely expanding treatment to milder cases).

Twenty-four studies that reported the direct outcomes of medication across the spectrum of symptoms supported substantial short-term symptom reduction.^196,241,247,275,276,277,278,279,280,281,282,283,284,285,286,287,288,289,290,291,292,293,294,295^ However, only 3 studies^245,297,298^ reported long-term follow-up beyond active treatment, finding no difference in symptoms between youths who were treated and those who were untreated in later life, and another study^296^ found no difference in symptoms after a 48-hour washout period. In terms of harms, active treatment was commonly associated with mild and moderate adverse events^276,278,280,281,286,289,291,292,294,295,299,338,339,340,341,342^ and high discontinuation rates.^275,285,290,341,347,348^ Ten studies mentioned unsatisfactory reporting of harms.^282,287,291,294,338,340,344,345,346^

Indirect treatment effects (across the spectrum of ADHD symptoms) were documented for diverse outcomes, including academic,^64,233,244,245,246,247,248,249,250,251,252,253,254,255,256^ cardiovascular,^267,268,269,270,271,272,273,274^ physical,^267,297,300,301,302,303,304,305,306,307,308,309,310^ psychological,^311,312,313,314,315,316,327,328,329^ social and emotional,^150,246,256,265,331,333,334,335,336,337^ and accidents.^150,244,257,258,259,260,261,262,263,264,265,266^ We found evidence of benefits for academic outcomes,^241,242,243,248,249,250,251,252,253,254,255^ injuries,^259,260,261,262,263^ hospital admissions,^150,257,259^ criminal behavior,^150,333^ and quality of life.^334,335,336^ In addition, harmful outcomes were evident for heart rate and cardiovascular events,^268,269,270,272,273,274^ growth^297,302,303,304,305,306,307,308^ and weight,^304,305,308^ risk for psychosis and tics,^311,312^ and stimulant misuse or poisoning.^266,322,323,324^ Treatment was associated with reduced physical activity in 2 studies. For suicidal behavior as well as emotional and social impairment, we could not find any favorable or unfavorable patterns.

The findings suggest that relatively large symptom reductions through medication translate to modest decreases in functional impairment at best while carrying risks. This ratio is likely worse for youth with milder ADHD in which large symptom reductions are impossible.

## Discussion

To our knowledge, this study is the first systematic scoping review on overdiagnosis of ADHD in youths. We found evidence of overdiagnosis and overtreatment of ADHD. We confirmed a large reservoir of diagnosable ADHD, consistently increasing rates of ADHD diagnosis and treatment, and a large proportion of newly detected cases with milder symptoms (in which harms may outweigh smaller benefits of diagnosis and treatment). Furthermore, we found few studies that assessed symptom severity among extra cases diagnosed through expanded disease definitions as well as the balance of benefits and harms for these individuals, representing a critical evidence gap.

### Implications of Findings

Our findings have implications for these individuals, who may be harmed by overdiagnosis and the adverse effects of medication during childhood, adolescence, and even adulthood. These findings are also relevant to the growing number of adults being newly diagnosed with ADHD^4^ and may be applicable to other conditions, such as autism.^358^

Several important research questions emerged during this review. Larger studies need to be conducted to confirm whether the additional ADHD cases now being diagnosed have milder symptoms. Future research is also required to evaluate whether diagnosing and treating milder ADHD cases may carry net harm. To reduce health and educational inequities, resources must be shifted from the overdiagnosis and overtreatment of ADHD to the needs of youths with more severe symptoms and who are more likely to benefit, including those currently underdiagnosed. Our research focused on overdiagnosis, and we did not address the misdiagnosis and underdiagnosis of ADHD. Although they are outside of the scope of this study, misdiagnosis and underdiagnosis are important complementary issues in a broader discussion of the principles of “right” care^359^ and equitable use of health care resources.^360^

We recommend that practitioners, parents, and teachers carefully weigh the potential benefits and harms that can accompany ADHD diagnosis and treatment, especially when identifying youths (or adults) with milder symptoms. For this group, the benefits of diagnosis and treatment may be considerably reduced or outweighed by harms.

An option to improve the balance of benefit to harm in practice may be to follow a stepped-diagnosis approach, as described by Batstra et al^9^ and Thomas et al.^25^ This approach incorporates the valid need for efficient diagnosis and treatment for severe cases as well as a watch-and-wait approach for borderline cases. It echoes management by active surveillance of low-risk prostate, breast, and thyroid cancers, in which overdiagnosis occurs frequently,^361^ and it ensures that resources are allocated where they are needed most and will be most valuable.^359^

### Strengths and Limitations

This study has several strengths. The 5-question framework enabled us to undertake a systematic scoping review, in accordance with international standards,^26^ to synthesize a large, heterogeneous set of studies. We undertook a critical appraisal of the included studies^32^ that allowed us to evaluate the quality of the evidence collected globally over many decades. Previous analyses focused on specific aspects of ADHD overdiagnosis, such as the existence of a relative age effect,^72^ differences between diagnosis and phenotype trends,^21^ or outcomes of medication.^294,346^ Although they contributed important evidence to the literature, these earlier studies were unable to draw overall conclusions on overdiagnosis of ADHD, which we were able to do.

This study also has several limitations. First, despite the inclusion of studies from many countries over a long period, these findings may not be applicable to all demographic groups. Given the scope of this study, we restricted the evidence for questions 4 and 5 to pharmacological treatment of ADHD, which is the most common and controversial treatment. Second, this review is limited by the availability and quality of evidence. Although our confidence in the outcomes for questions 2 and 4 is high, many studies included for questions 1, 3, and 5 were at high risk of bias. Third, parents or teachers were often the sole reporters of potentially subjective measures (eg, symptom severity, quality of life, and consequences of diagnosis). This lack of self-reported data means that it is unknown whether benefits and harms may have been reported differently by the youths themselves.

## Conclusions

In this systematic scoping review, we found convincing evidence of ADHD overdiagnosis and overtreatment in children and adolescents. Despite an abundance of research in the field of ADHD, gaps in evidence remain. In particular, high-quality studies on the long-term benefits and harms of diagnosing and treating ADHD in young people with milder symptoms are needed to inform safe and equitable practice and policy.

## Notes

Supplement 1. **eAppendix 1.** MEDLINE Search Strategy **eAppendix 2.** Data Extraction Template **eAppendix 3.** Flow Diagram of Included Studies **eAppendix 4.** Qualitative Data Extraction **eAppendix 5.** Critical Appraisal Results **eAppendix 6.** Results Table **eReferences**

Supplement 2. **Data Extraction Results**
