# Supplementary material for: Overdiagnosis of Attention-Deficit/Hyperactivity Disorder in Children and Adolescents: A Systematic Scoping Review
Source: JAMA Netw Open. 2021 Apr 12;4(4):e215335. doi: 10.1001/jamanetworkopen.2021.5335 (PMC8042533; doi:10.1001/jamanetworkopen.2021.5335)
Supplement: Supplement 1. — eAppendix 1. MEDLINE Search Strategy eAppendix 2. Data Extraction Template eAppendix 3. Flow Diagram of Included Studies eAppendix 4. Qualitative Data Extraction eAppendix 5. Critical Appraisal Results eAppendix 6. Results Table eReferences [file jamanetwopen-e215335-s001.pdf]

Supplementary Online Content

Kazda L, Bell K, Thomas R, McGeechan K, Sims R, Barratt A. Overdiagnosis of attention-deficit/hyperactivity disorder in children and adolescents: a systematic scoping review. *JAMA Netw Open*. 2021;4(4):e215335. doi:10.1001/jamanetworkopen.2021.5335

eAppendix 1. MEDLINE Search Strategy

eAppendix 2. Data Extraction Template

eAppendix 3. Flow Diagram of Included Studies

eAppendix 4. Qualitative Data Extraction

eAppendix 5. Critical Appraisal Results

eAppendix 6. Results Table

eReferences

This supplementary material has been provided by the authors to give readers additional information about their work.

© 2021 Kazda L et al. *JAMA Network Open.*

eAppendix 1. MEDLINE Search Strategy

<table>
<tr><th>0. Is ADHD overdiagnosed?</th><th>1. Is there potential for increased diagnosis?</th><th>2. Is diagnosis actually increased?</th><th>3. Are additional cases subclinical or low risk?</th><th>4. Are additional cases treated?</th><th>5.b) Might harms outweigh benefits for treatment?</th><th>5.a) Might harms outweigh benefits for diagnosis?</th></tr>
<tr><td colspan="7">((exp Attention Deficit Disorder with Hyperactivity/ OR adhd.ti. OR hyperkinesis.ti. OR exp Hyperkinesis/ OR Attention Deficit Hyperactivity Disorder.ti. OR Attention Deficit Disorder with Hyperactivity.ti. OR Hyperkinetic Disorder.ti.)</td></tr>
<tr><td colspan="7">AND</td></tr>
<tr><td colspan="7">(Child*.tw. OR Child/ OR Adolescen*.tw. OR exp Adolescent/ OR exp Infant/ OR Infan*.tw. OR Minors/ OR p?ediatric*.tw. OR Pediatrics/ OR primary school*.tw. OR school*.tw. OR kindergarten.tw. OR pre-school.tw. OR Pre School.tw. OR elementary school.tw. OR student*.tw. OR secondary school.tw. OR Schools/ OR high school*.tw. OR Child Psychiatry/ OR Adolescent Psychiatry/)</td></tr>
<tr><td colspan="7">AND</td></tr>
<tr><td rowspan="3">(overdiagnos*.mp. OR over diagnos*.mp. OR overtest*.mp. OR over test*.mp. OR exp Medical Overuse/ OR overuse*.mp. OR over use*.mp. OR overdetect*.mp. OR over detect*.mp. OR insignificant disease.mp. OR overtreat*.mp. OR over treat*.mp. OR inconsequential disease.mp. OR overmedical*.mp. OR unnecessary procedure*.mp. OR exp Unnecessary Procedures/ OR pseudodisease.mp. OR pseudo disease.mp. OR "too much medicine".mp. OR nondisease.mp. OR non</td><td>((continuum OR continual* OR continuous* OR dimension* OR categoric* OR spectrum OR subthreshold OR threshold OR full syndrome OR dichotomous OR linear association OR distribution of symptom* OR full symptom*).tw))</td><td>(prevalence/ OR prevalen*.ti. OR incidence/ OR inciden*.ti. OR frequency.ti. OR rate.ti. OR definition*.ti. OR diagnos*.ti. OR Diagnosis/ OR phenotype.ti)</td><td>(severity.tw. or impair*.tw. or mild.tw. or moderate.tw. or severe.tw. or extreme.tw. or subclinical.tw. or subthreshold.tw)</td><td colspan="2">(treatment*.ti. or exp Therapeutics/ or medication*.ti. or pharma*.ti. or Pharmaceutical Preparations/ or Ritalin.ti. or exp Methylphenidate/ or Central Nervous System Stimulants/ or stimulant*.ti. or drug*.ti. or therapeutics/ or drug therapy/ or therapeutic*.ti. or Methylphenidate.ti. or psychostimulant*.ti. or Dexmethylphenidate.ti. or Dexmethylphenidate Hydrochloride/ or Atomoxetine*.ti. or Atomoxetine Hydrochloride/ or nonpsychostimulant.ti. or exp Amphetamines/ or amphetamine*.ti. or adderrall.ti or antipsychotic*.ti. or Antipsychotic Agents/)</td><td>(label*.mp.))</td></tr>
<tr><td>NOT</td><td>AND</td><td>AND</td><td>AND</td><td>AND</td><td>NOT</td></tr>
<tr><td>(Autism.ti. OR Autistic.ti. OR exp Autistic Disorder/ OR exp Autism spectrum Disorder/)</td><td>(trend*.tw. OR field trial*.tw. OR follow-up studies/ OR follow-up.tw. OR chang*.tw. OR</td><td>(prevalence/ OR prevalen*.ti. OR incidence/ OR inciden*.ti. OR frequency.ti. OR</td><td>(trend*.ti. OR change*.ti. OR variation*.ti. OR vary*.ti. OR increase*.ti. OR</td><td>(Treatment Outcome/ or outcome*.tw. OR consequence*.tw. OR impact*.tw.)
AND</td><td>(off-label.tw. OR Open-Label.tw.)</td></tr>
</table>

© 2021 Kazda L et al. *JAMA Network Open.*

disease.mp. OR "false positive*".mp. OR overdefinition*.mp. OR over definition*.mp. OR misdiagnos*.mp. or Diagnostic Errors OR variation of care.mp. OR medicali*.mp. OR Medicalization/))		variation*.tw. OR vary*.tw. OR increas*.tw. OR decreas*.tw. OR pattern*.tw. OR expan*.tw.))	rate.ti. OR trend*.ti. OR change*.ti. OR variation*.ti. OR vary*.ti. OR increase*.ti. OR decrease*.ti. OR pattern*.ti. Or expand*.ti.))	decrease*.ti. OR pattern*.ti. Or expand*.ti. OR overprescri*.ti. OR prescri*.ti. OR underprescri*.ti.))	(Patient Harm/ or harm*.tw. OR Cost-Benefit Analysis/ OR benefit*.tw. OR cost*.tw. OR Risk/ or risk*.tw. or improv*.tw. or positive.tw. or negative.tw. OR worse.tw. or better.tw. OR adverse.tw. OR effect*.tw.)	
					AND	
					(((meta analysis or "systematic review").pt.) OR (Cohort.tw. OR longitud*.tw. OR observation*.tw. OR follow-up.tw. OR registries/ OR longitudinal studies/)))	
NOT						
((autobiography OR bibliography OR biography OR case reports OR comment OR congress OR consensus development conference, nih OR dataset OR dictionary OR directory OR editorial OR expression of concern OR festschrift OR government document OR guideline OR interactive tutorial OR lecture OR legal case OR legislation OR letter OR news OR newspaper article OR patient education handout OR personal narrative OR portrait OR scientific integrity review OR technical report OR video-audio media).mp. OR webcasts.pt.)						
AND						
limit search to (english language and yr="1979 -Current")						

© 2021 Kazda L et al. *JAMA Network Open.*

eAppendix 2. Data Extraction Template

Study Details

- Author
- Year
- Title
- Study Type
- Study Characteristics
- Main Study Focus
- Study length
- Study Year(s)

Population Characteristics

- Population size
- Setting/ Data Source
- Population Age
- Population Sex
- Country/ Region

Miscellaneous

- Funding
- Conflicts of Interest
- Potentially Relevant References

Question 0-4 specific items

- Main relevant focus
- Other relevant focus
- Outcome(s)
- Measure(s)
- Instrument(s)
- Source(s)
- Effect(s)
- Key relevant finding

Question 5a/b specific items

- Main relevant focus
- Other relevant focus
- Outcome(s)
- Measure(s)
- Instrument(s)
- Source(s)
- Exposure(s)
- Comparator(s)
- Effect(s)
- Key relevant finding

© 2021 Kazda L et al. *JAMA Network Open.*

eAppendix 3. Flow Diagram of Included Studies

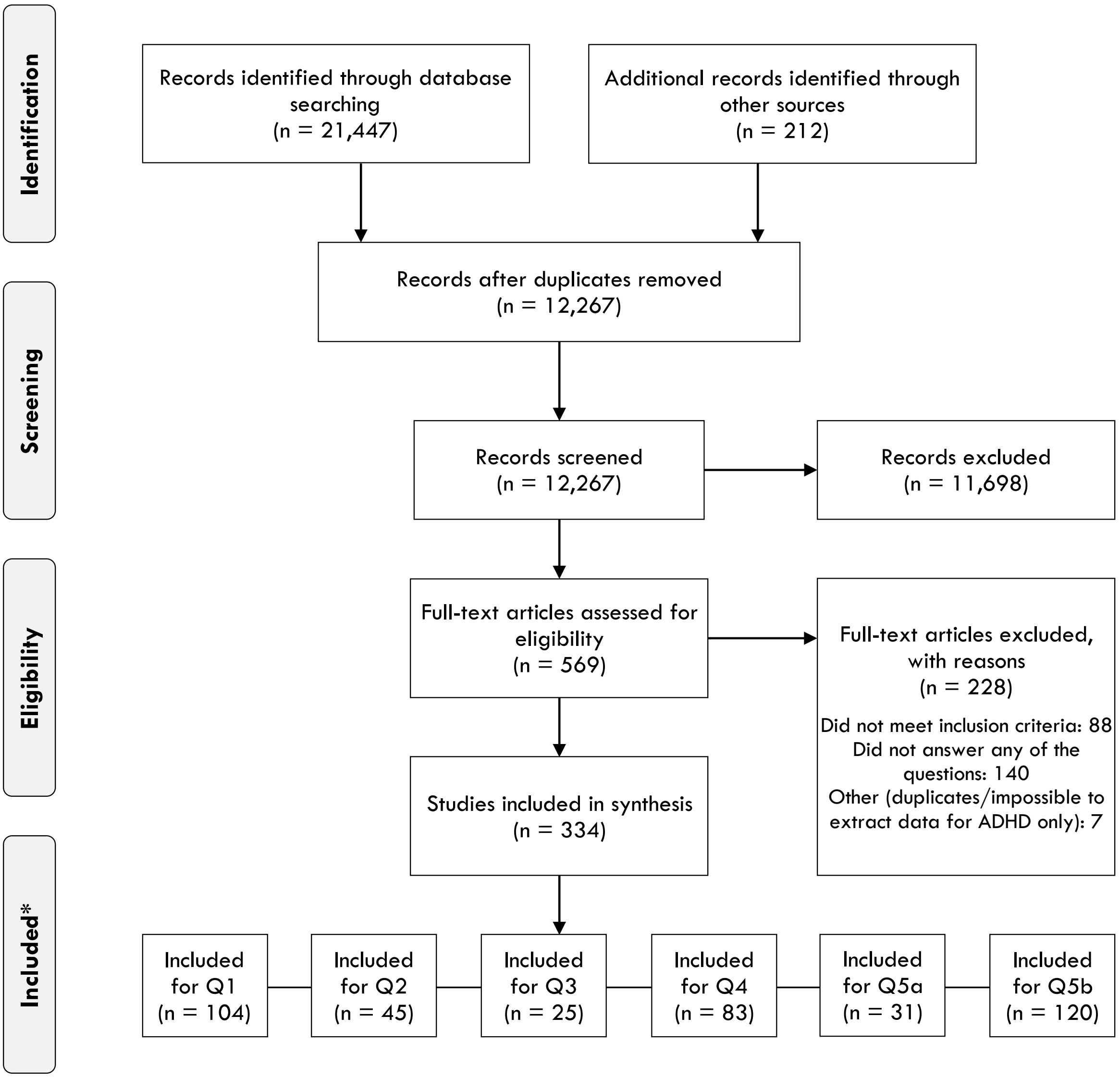

*It was possible for included studies to supply data for more than one question

Adapted from Moher D, et al (2009) Preferred Reporting Items for Systematic Reviews and MetaAnalyses: The PRISMA Statement[1]

© 2021 Kazda L et al. *JAMA Network Open.*

eAppendix 4. Qualitative Data Extraction

I. <u>Potential benefits of an ADHD diagnosis</u>

1. Empowerment through explanation for problems

a. Increased legitimacy and validation

Allan et al -2014

1. Sometimes we can have referrals or children or parents of very affluent people who are looking for reasons for antisocial behaviour that are a disorder [and] therefore explains [the] behaviour but we also have parents with children who are very impoverished and come from impoverished backgrounds emotionally and socioeconomically and you know sometimes that disorder label can increase – well, it increases their benefits. (Professional 10, child mental health)

Damico et al -1995

1. (21) "He's so much better now. Even when he doesn't do very well, at least we know that there's a reason-even the teachers do. . . . He's not a bad boy . . . or lazy. . . . He has a medical problem. . . . It explains so much! I think back to when he was a baby and then when he was biting kids in preschool-all explained by the ADD. That doctor appointment changed our lives!" [Interview; T.C. 5-26-92]

Klasen -2000

1. Their immediate reaction was often a sense of great relief. For the first time their definition of the reality of their child's problem had been confirmed by others and thus legitimated
2. being able to name the problem of their child seems to give parents a sense of power and agency. In contemporary society the medical framework, especially the diagnosis, can provide patients and parents with a powerful tool that carries considerable social legitimacy

Moore -2017

1. Labelling can be helpful, as practitioners often saw a diagnosis as validating problematic behaviour as a medical condition, which assists children and their parents to understand their difficulties and gives further access to support in school. Indeed, one practitioner said 'I think having that diagnosis can help them access other support" (Maisy, SENCo, Primary).

Rogalin et al -2015

1. the ADHD diagnosis gives struggle a name, an official validation, and it furnishes a sense of comfort with understanding the problem (Danforth and Navarro 2001). In an educational environment this explanation renders the problematic behaviors more reasonable as it gives a socially acceptable meaning to the behaviors as caused by a disorder avoiding that children are in different ways excluded for being just messy kids.

b. Increased understanding, sympathy and reassurance

Andersson Frondelus et al -2019

1. In the participants' experiences, being diagnosed essentially meant accommodating their differences in terms of ADHD-related traits. This meant seeing both the good and the bad of

© 2021 Kazda L et al. *JAMA Network Open.*

their condition as interrelated parts of themselves, like many others who shared these traits. Seeking acceptance and a sense of normality was important

2. All participants described early, painful memories of being different and feeling frustrated and misunderstood, before being diagnosed.
3. The label provided a name for their condition, which made it easier to explain their difficulties.
4. Parents, siblings, close friends and teachers could understand better and help to cope with difficult situations: “My two closest friends know [about the ADHD diagnosis] and they understand me better.”

Carr-Fanning et al -2018

1. Almost unanimously, parents were adamant that diagnosis was essential. They identified benefits in understanding ADHD in terms of helping to support their child and becoming self-empowered
2. ... I was delighted to get a diagnosis ... because I couldn’t understand what was wrong with my son ... I just threw myself into it tried to find out everything ... the empowerment of understanding the condition ... you start to see your child in a completely different light. Instead of the bold child that’s ALWAYS getting given out to that their name is ‘always’ worn out. And you are basically bashing them up...you start to see why ... You start to understand ...you start do things differently ... (mother, son 10 years).

Hamed et al -2015

1. On the other hand, some individuals have described that the extra attention from their teachers or parents that accompanied the diagnosis of ADHD helped affirm and build confidence (79, 80).

Klasen -2000

1. Generally, parents thought that seeing the child’s problems in medical terms helped them to understand that the behavior was not under the control of the child. Although the nonmedical way of conceptualizing the problem (like “naughty” or “stupid”) can lead to critical comments and anger, a medical explanation can help parents to develop their own metaphors in order to reframe the child’s behavior in a less blaming way. According to parents, seeing their child as “ill” rather than “bad” leads to increased sympathy and decreased anger
2. Causal beliefs of responsibility for failure promote anger, while causal beliefs of non responsibility promote sympathy.
3. they found it easier to see their child’s acts as nondeliberate. According to them, this increased their sympathy for their child and reduced their anger.

Moore -2017

1. Practitioners recognize the process of labelling occurring for children with ADHD. While the label may aid understanding and access to support, the negative aspects of labelling ‘can sometimes just compound them into their difficulties, rather than pull them out’ (Ryan, Pastoral Leader, Primary)

Rogalin et al -2015

1. Behavior becomes reasonable through the diagnosis. Participants describe the behavior of the children by attributing it to its cause: if behavior can be explained through ADHD than it is easier to socially accept these behaviors. Without knowing the “background”, the child remains just a messy kid, but if the diagnostic label is assigned to the child his or her actions become

© 2021 Kazda L et al. *JAMA Network Open.*

reasonable. The label is powerful as it adds another meaning to the behaviors of the children, both from the peers and the teachers' perspective.

If he would not have had the diagnosis, I don't think that his peers would have accepted him as much as they do now, because now there is an explanation to certain behaviors. (Leisure time teacher and assistant) We know the background (...). Background is important because one gains another understanding of the child. (Teacher)

2. (The diagnosis) is a way for the parents and for others surrounding the child, to know more exactly what this child needs, how to make this child function. (Leisure time teacher) You have to try to possess as much knowledge as possible to be able to be prepared on how to handle behavior. To find a sense of security in ones' own behavior. (Leisure time teacher)
3. Reassurance: As the problematic behaviors are symptoms of ADHD, i.e. symptoms caused by ADHD, the difficulties in school and elsewhere are a consequence of ADHD and not a consequence of lacking skills in parenting or of shortcomings in the educational area. In this way the responsibility of the problems is taken away from parents and education staff, reassuring them of the eligibility of their roles. The diagnostic label guarantees the adequacy of certain actions when relating to the child. In fact, it is felt that being aware of the diagnosis of a specific child, makes it easier to know how to behave in their regard.
4. it reassures professionals involved of their capacities and competences. The responsibility falls on the diagnosis and not on the adults surrounding the child

Wienen et al -2019

1. Five out of the 30 respondents point out that ADHD classification can bring peace and quiet, or put matters to rest. Respondent 27 described it thus: 'So I think, yeah, the peace of mind for that child, parents sometimes overlook it, but it is there all right.' Likewise, ADHD classification may offer peace of mind to parents too, as respondent 25 observed: 'And you notice that, that as soon as children have a diagnosis and there are new means available at home, ehr. . . yes, like it provides a kind of acceptance of, "oh look, now we know what is the matter. And now we can address it".' This accepting peace of mind also surfaces in contact between parents and the teacher, according to respondent 16: 'No, it gives peace of mind also in discussion with teachers. Like, that's what she is like, so how do we make it fly. Just clarity. And whether, to put it negatively, you really need a label for it, I don't know. As long as you have. . .well no, clarity and peace of mind. And that is better for the child, too. Also in your expectations and your. . . Well yeah, that sometimes you can expect a little more but you need to follow a different route or make things available in a different way.'
2. Sixteen out of 30 teachers mention ADHD classification as explanation of the fact that the teaching approach no longer works in relation to a child, or as confirmation that a particular approach that is already in use is deployed legitimately, that is, with good cause. Respondent 9 noted the lack of certainty that can beset teachers: 'When you notice that the actions that you undertake sort no effect again and again, as I have just described.' Respondent 14 mentioned instances where everything has been tried and has failed: 'When you conclude that you have tried everything but nothing has worked, and then it is really good for the child, but also for you as teacher.
3. Eleven out of the 30 respondents point out that ADHD classification brings greater empathy. Respondent 5, for example, noted the empathy a teacher has for a pupil: 'I think that it brings a bit of empathy. Or a lot of empathy, which means that you respond quite differently in most cases.'

© 2021 Kazda L et al. *JAMA Network Open.*

c. Reduced feelings of guilt, blame, fault, failure and anger

Allan et al -2014

1. with one possible reason being that some 'mainstream schools perhaps would be quite relieved to have their diagnosis because … it almost alleviates the blame that they're not doing the right thing' (Professional 6, specialist education).
2. non-diagnosis could come to imply fault.

Carr-Fanning et al -2018

1. In a similar manner, parents often described elation or relief when they received a diagnosis.
2. Young People (YP) who had not been diagnosed until adolescence described the frustration and repeated experiences of failure and distress prior to receiving a diagnosis, which was often a source of anger and resentment.
3. Parents reported that challenging behaviours were attributed to parenting (e.g. lack of discipline) and/or their child's lack of effort (e.g. 'lazy'), or wilful disobedience (e.g. 'bold brat'): "it was always HER she was the problem so she was constantly being told she was no good"
4. …some of the teachers I think they're like ADHD doesn't really exist they're just like you're just being bold looking for attention it's not really anything. I'm annoyed by that coz it's not true (female 17 years).

Comstock -2015

1. Similarly, there are many examples where AD/HD diagnosis gives these individuals a perceived distinct rationalization for various failures in life within these institutions. Important here are the kinds of failures that are attributed to the disorder. These failures are not referenced to any specific category of behavior, not even hyperactive or inattentive behavior, but rather to a troubled way of being. Here are some examples:

   "To finally be able, at the age of 39, to put a name to what I was repeatedly told was laziness or sloth is amazing enough. Once I explained that it was the cause of my sloppy work, poor behavior and other things all have noticed, then they could see the changes on meds, and all was fine with them. Why was I different? Why couldn't I cope with things that other people could? Why did I have such a difficult time holding down a job? Why were my finances in a mess? Why was I estranged from my family? Why couldn't I even begin to understand my two son's [sic] even though they acted like me?? "

   These individuals frequently see themselves as having failed to manage their resources in order to secure the best possible returns. They represent not a failed venture so much as a non-venture, one that was more unmanaged than mismanaged, outside of a knowledge about authentic self-representation, and therefore doomed from the beginning. A revelation about the biological origins of their problems, and the adopting of an AD/HD identity, is almost always viewed by these individuals as the first step to success.

Damico et al -1995

1. (5) "He was always labeled as 'lazy' . . . 'not working up to potential,' 'unmotivated' . . . oh, I hate those words-I hate them! There's always a reason for these words and their behaviors-well, do something about it! Don't just say the words! Just find the reason . . . but you can't say that, that wo . . . would reflect on the teaching style . . . that is attacking the teacher. Oh, no!

© 2021 Kazda L et al. *JAMA Network Open.*

We are just parents. We don't know anything. . . . It's no wonder we go out outside of the school." [Interview; N.S. 6-14-92]

2. "Bobby and I went to the school for a meeting with her. 'He's bright,' she said, 'but he just doesn't apply himself.' Now, I had enough. 'You ought to get him tested,' I said. 'You have programs here to help his reading and his learning.' She insisted that his problems were not due to any learning difficulties. . . . It was just his lack of motivation. . . . She probably blamed us. In the fourth grade we saw the same things. . . . Poor work from a smart boy. Ray is very good and he tries to please people. . . . It was a problem for him at school, and they just didn't help at all." [Interview; G.H. 6-1-92]
3. This confirmatory reaction has a more subtle implication, one that was expressed by one of the physicians during the interview phase (see example 17): If there is a real problem, medical or biological in nature, then the behaviors and difficulties that the parents have had to address have an explanation. That means that the problems are not due to "character flaws" or "poor parenting" or even "poor teaching" but, rather, to that problem within the child.
4. In effect, as Reid, Maag, and Vasa (1994) have suggested, the child, parents, and teachers are given a "no fault label.

Klasen -2000

1. Knowing that their child’s troubles could be due to an illness often helped parents to stop internalizing the problem as their own failure, weakness, or imagination.
2. The great majority of parents, however, felt that their child had already been labeled by his or her environment before getting a medical diagnosis. They regarded the nonmedical labels such as “stupid,” “lazy,” and “naughty” as even more damaging than medical ones for the child and for the family because such terms produced feelings of guilt and low self-esteem

Wienen et al -2019

1. respondent 20 mentions the same as an advantage for parents: ‘I think it is a relief for parents, that they really, that you can explain the behaviour. . .and that you know like, well this could be because of the ADHD. I think that is rather nice for parents.’
2. Five out of the 30 respondents point out that an ADHD classification leads to the removal of guilt or blame. This removal involves different actors, according to the teachers. It concerns removal of guilt or blame from the child, from the parent(s) and also removal of guilt or blame from the teacher by the parent(s). The latter may be the case if the parent(s) initially perceive the notable behaviour of the child to be the consequence of the teacher’s ways of doing. Respondent 16 talked about the removal of guilt or blame from the child: ‘I sometimes think that acknowledgement, for the child. . .you are not to blame, you just have it.’ Respondent 22 put it more generally: ‘I think that once parents know that my child has ADHD, it may perhaps remove a bit of uncertainty, like well, he’s got it, he is like that, nothing we can do about that.’ And finally, respondent 12 claimed that teachers are quickly blamed in cases where children are without ADHD classification but show disruptive behaviour: ‘which makes that parents say, “well, the teacher is to blame”’.
3. A large number of teachers report, in the interviews we have taken with them, that ADHD classification brings, in particular, a new shared starting point in the dialogue between parents and teachers about educating children. This new starting point can arise because an ADHD classification removes blame and guilt from all of teachers, parents and pupils: the notable behaviour is entirely attributed to ‘the disorder’ (Broomhead 2013; Moore et al. 2017; Rogalin and Nencini 2015), despite this being a product of questionable science and faulty

© 2021 Kazda L et al. *JAMA Network Open.*

reasoning. This shared 'new beginning' creates new room for mutual understanding between parents and teachers and obviates the need for blaming one another for the behaviour perceived in the child. The availability of ADHD classification thereby offers a supposedly independent or external validation of the conclusion that 'something is the matter' with the child so that parents and teachers can start a new working relationship free from guilt and blame – in shared innocence, as it were.

2. Empowerment through increased control

a. Reduced uncertainty leads to reclaiming of identity/ feelings of belonging

Andersson Frondelius et al -2019

1. In their view, normality was related to self-respect and to identification with the ADHD population.
2. the adolescents' experiences of being diagnosed with ADHD essentially meant a process towards understanding their traits as both 'odd' and 'unique', and realising how common their problems were.
3. Several participants described being proud of their diagnosis; identifying with and admiring certain people with ADHD. This finding may reflect the adolescents' desire to feel that they belong,…

Comstock -2015

1. One can see here that although AD/HD causes a misdirection of (unmanaged) energies, claiming the disorder as an identity ("My goal with ADD ...") allows the individual to positively manage these energies.
2. As we can see in this passage, the diagnosis of AD/HD (self or otherwise) is usually depicted as the defining moment by which the observing self takes over the disorganized, unobservant self: I've yet to recieve [sic] an official diagnosis, but I'm meeting with a Psychiatrist this Friday to hopefully get both confirmation for that which I firmly believe to be the reason for so many of my unexplained tendancies/behaviors [sic], and to get on track with treating this ever-increasing burdon [sic] that is living with myself and my mind.

   Significantly here, it is because of the fact that the individual cannot make meaning of his or her behaviors—not because he or she is troubled by behaviors considered deviant or abnormal—that the individual believes something is wrong. This individual's "unexplained tendencies/behaviors," it is asserted, will finally be explained with an official diagnosis that will serve as the principle by which the one self makes meaning of the other self, in short, as an identity. Diagnosis becomes the moment that the previously troubled individual confirms a new identity within and on the side of a knowledge about the proper way to live and the truth about human nature (for this reason genetics, and speculating on the AD/HD of family members, is a frequent theme in the posts).
3. For these individuals, the AD/HD identity is rooted in and at every point related to the knowledge that individuals are "naturally" self enterprising and self-interested. And in adopting the AD/HD identity, they position themselves firmly on the side of truth and knowledge. This turning to the hard truth of biology is consistently evident in these posts, especially in the frequent metaphorical construction of AD/HD diagnosis and treatment as a kind of "salvation." These terms of spiritual awakening appear frequently.

Klasen -2000

© 2021 Kazda L et al. *JAMA Network Open.*

1. They realized that they were not alone with their problem, and the chaos of different pressures and disturbing observations and experiences started to be restructured and framed in terms of the disorder.

a. Increased self-esteem and confidence

Andersson Frondelius et al -2019

1. Being diagnosed with ADHD meant to recognise some traits as positive and even appreciate them as a gift, feeling special and unique compared with most people.
2. While developing an understanding of the complexity of ADHD and its commonness, the participants gained confidence and a sense of dignity.

Hamed et al -2015

1. On the other hand, some individuals have described that the extra attention from their teachers or parents that accompanied the diagnosis of ADHD helped affirm and build confidence (79, 80).
2. Individuals diagnosed late reported low self-esteem and underachievement due to repetitive experiences of failure during childhood (80).

Klasen -2000

1. Having a diagnosis provided some parents with the confidence to face clinical, social, and school services and request the help that they or their child needed.

b. Expectation of solution

Andersson Frondelius et al -2019

1. With the diagnostic label as a door opener, the participants expected that their problems would be reduced or disappear. They hoped to finally calm down, to be able to focus and perform successfully.
2. The adolescents in this study put substantial hope for change in being diagnosed. The diagnosis was expected to change their life, but their hope was not always fulfilled.

Damico et al -1995

1. This second institutional contact was usually much more successful from the perspective of the parents. They believed that their children's problems had finally been addressed.
2. a parent expressed satisfaction with the medical diagnosis and the opinion that the problem should be solved by medical intervention. The views expressed in this excerpt were fairly typical: (14) "Well, now that she's diagnosed and on medication, the problems should vanish. We now know that the problems were inside her brain. The doctor said that her brain is like a switchboard that can't receive and handle all the calls because it's too easily distracted. With the medication, that's changed. I don't know how the teachers could help her more . . . maybe work on her learning problems?" [Interview; Q.A. 9-24921

Klasen -2000

1. Even though a diagnosis also brought some ambivalent feelings for some parents, it generally was seen as a constructive step forward in dealing with the serious problems of raising a hyperactive child.

© 2021 Kazda L et al. *JAMA Network Open.*

2. Receiving the diagnosis gave parents hope that the situation could improve. Armed with an explanation for their child's problems that did not imply their own guilt, many felt more in control of the circumstances of their lives and more able to work out ways of understanding and solving their problems. Having a diagnosis provided some parents with the confidence to face clinical, social, and school services and request the help that they or their child needed.
3. In general, parents were activated by the diagnosis. They found materials on hyperactivity, joined self-help groups, tried new parenting techniques, and seemed more hopeful.

Wienen et al -2019

1. Eight out of the 30 respondents note that an ADHD classification leads to a 'new' shared starting point in the collaboration between parents and teachers. Respondent 27 described it as follows: 'While with those parents whose child has been diagnosed there is often also a solution, medication or support, in any case, something that you can bat about. In those cases it becomes more of a collaborative little project I think, through which you can help the child.' Respondent 5 also talked about the shared perspective that emerges following classification: 'Sure, a clearer picture really, also clear towards parents. . .and then you do need to point all noses in the same direction, so to say. And when that is all sorted you can say, okay, what do we now need.

3. Enablement to support

a. Increased ability to seek, receive and accept support

Comstock -2015

1. Many individuals similarly embrace the AD/HD identity in more or less enthusiastic terms, based on the benefits they expect to see at work, school, in domestic relationships (indeed, most posts touch on at least one of these three institutions).

Klasen -2000

1. Having a diagnosis provided some parents with the confidence to face clinical, social, and school services and request the help that they or their child needed.
2. After the diagnosis the thought of having to learn special strategies to deal with her hyperactive child was much less threatening

Moore -2017

1. Labelling can be helpful, as practitioners often saw a diagnosis as validating problematic behaviour as a medical condition, which assists children and their parents to understand their difficulties and gives further access to support in school. Indeed, one practitioner said 'I think having that diagnosis can help them access other support" (Maisy, SENCo, Primary).
2. Practitioners recognize the process of labelling occurring for children with ADHD. While the label may aid understanding and access to support, the negative aspects of labelling 'can sometimes just compound them into their difficulties, rather than pull them out' (Ryan, Pastoral Leader, Primary)

Wienen et al -2019

1. A large number of teachers report, in the interviews we have taken with them, that ADHD classification brings, in particular, a new shared starting point in the dialogue between parents and teachers about educating children. This new starting point can arise because an ADHD classification removes blame and guilt from all of teachers, parents and pupils: the notable

© 2021 Kazda L et al. *JAMA Network Open.*

behaviour is entirely attributed to 'the disorder' (Broomhead 2013; Moore et al. 2017; Rogalin and Nencini 2015), despite this being a product of questionable science and faulty reasoning. This shared 'new beginning' creates new room for mutual understanding between parents and teachers and obviates the need for blaming one another for the behaviour perceived in the child. The availability of ADHD classification thereby offers a supposedly independent or external validation of the conclusion that 'something is the matter' with the child so that parents and teachers can start a new working relationship free from guilt and blame – in shared innocence, as it were.

b. Educational support

Damico et al -1995

1. Once the diagnosis was completed and the label was received, several additional events took place. In thirteen of the fourteen cases, physicians or psychologists recommended medication. In eleven of the thirteen cases, this recommendation was accepted by the parents. The other primary result of the label was that the parents returned to the schools with a diagnosis of ADHD in hand. In eight of the fourteen cases, the schools reacted immediately to the label and provided some services or accommodations for the students. In the other six cases, the schools were uncooperative, but eventually relented and provided some services or accommodations in five instances.
2. Services or accommodations that had not been previously provided were now made available. This occurred in thirteen of the fourteen cases. The following comment from a regular classroom teacher demonstrates this tendency for acquiescence: (10) "I have so many parents that are concerned. Whoever thinks that parents aren't concerned don't work in this school. So I get a lot of complaints and requests for more work or more help. I can't give it all. Now, you give me a medical diagnosis, something on paper and based on medical science, now that will get my attention." [Interview; K.B. 8-5-92]
3. When the formal label was delivered to the schools, the documentation of the disability label acted as a catalyst for actions requested earlier by the parents. Usually, evaluations, accommodations, and services not previously offered to the child and parents were now provided. The label, therefore, had a reactive power over the schools, the parents, and even the children. Perhaps this is a primary reason for the rapid growth of ADHD as a disability label over the last several years. Parents are increasingly recognizing that the concerns they express to the schools are not taken seriously until a disability label is assigned.

Rogalin et al -2015

1. If a child does not have a diagnosis but does behave like a child with ADHD, the same resources will not be given, this child will become just a rowdy kid, he might get detention, maybe he will be taken away from the class, but the discussion will not be concerning the fact that this child needs help/ assistance. (Leisure time teacher and assistant)
2. To see behaviours as symptoms of a disorder rather than unreasonable and unexplainable actions changes the interventions that are made in regards to the children. Assistance and support are the main focus in the presence of a diagnosis whereas detention and expulsion from the classroom are more likely if the child does not have a diagnosis.

Wienen et al -2019

1. Eleven out of the 30 teachers point out that an ADHD classification leads to new approaches, ideas, medication and right to support. As respondent 4 noted, 'So in order to better support these children by way of an assistant or whatever kind of effort, we need a diagnosis'. Some

teachers, including respondent 25, point out that new ideas and pointers may arise: ‘Yes, just purely those practical things like, how can I help the child. Also, because, especially for the child to find his or her own way in that.’

2. Eight out of the 30 respondents note that an ADHD classification leads to a ‘new’ shared starting point in the collaboration between parents and teachers. Respondent 27 described it as follows: ‘While with those parents whose child has been diagnosed there is often also a solution, medication or support, in any case, something that you can bat about. In those cases it becomes more of a collaborative little project I think, through which you can help the child.’ Respondent 5 also talked about the shared perspective that emerges following classification: ‘Sure, a clearer picture really, also clear towards parents. . .and then you do need to point all noses in the same direction, so to say. And when that is all sorted you can say, okay, what do we now need.

c. Financial support

Allan et al -2014

1. Sometimes we can have referrals or children or parents of very affluent people who are looking for reasons for antisocial behaviour that are a disorder [and] therefore explains [the] behaviour but we also have parents with children who are very impoverished and come from impoverished backgrounds emotionally and socioeconomically and you know sometimes that disorder label can increase – well, it increases their benefits. (Professional 10, child mental health)
2. This pursuit of diagnosis by impoverished people, driven by economic need, highlights the problems that can be caused by the medicalisation of poverty (Schram 2000).

d. Medical/ psychological support

Carr-Fanning et al -2018

1. Parents described how delayed diagnosis resulted in significant consequences and secondary problems, which led to the diagnosis. For example, a mother described her daughter’s (17 years) overdose at the age of 13 years as a cry for help.

Damico et al -1995

1. Once the diagnosis was completed and the label was received, several additional events took place. In thirteen of the fourteen cases, physicians or psychologists recommended medication. In eleven of the thirteen cases, this recommendation was accepted by the parents. The other primary result of the label was that the parents returned to the schools with a diagnosis of ADHD in hand. In eight of the fourteen cases, the schools reacted immediately to the label and provided some services or accommodations for the students. In the other six cases, the schools were uncooperative, but eventually relented and provided some services or accommodations in five instances
2. A parent expressed satisfaction with the medical diagnosis and the opinion that the problem should be solved by medical intervention. The views expressed in this excerpt were fairly typical: (14) "Well, now that she's diagnosed and on medication, the problems should vanish. We now know that the problems were inside her brain. The doctor said that her brain is like a switchboard that can't receive and handle all the calls because it's too easily distracted. With the medication, that's changed. I don't know how the teachers could help her more…maybe work on her learning problems?" [Interview; Q.A. 9-24921

© 2021 Kazda L et al. *JAMA Network Open.*

Hamed et al -2015

1. One of the major consequences of ADHD not being diagnosed is a lack of treatment. Untreated ADHD can pose a tremendous amount of psychological, financial, academic, and social burden to the individual and the community, which reflects the importance of diagnosing and treating the disorder (23, 66). While treatment has not been shown to completely "normalize" the developmental trajectory of individuals with ADHD, individuals with ADHD who do not receive treatment have poorer long-term outcomes compared to those that are treated (51). Untreated ADHD during childhood is a risk factor for later adult mental health issues, which extend beyond impairment in academics (66). A lack of treatment for ADHD also impairs social and occupational functioning and increases the likelihood of developing comorbid disorders like anxiety, depression, personality disorders, antisocial behaviours, and SUD (66, 67). Many mechanisms may be at work linking undiagnosed ADHD to vulnerabilities (27).
2. The prevalence of undiagnosed ADHD within a substance treatment population was approximately fivefold higher than the general population (66) suggesting that undiagnosed ADHD may have substance abuse requiring treatment as a consequence.
3. Also, as reviewed previously, the MRRs of individuals with delayed diagnosis of ADHD has been shown to be significantly higher than those diagnosed earlier, suggesting that a lack of diagnosis may accumulate risks to mortality (78).

Malacrida –2004

1. Many mothers reported being sent for interventions even though a label was not provided. They described multiple diagnoses that were vague and often not very helpful, including poor muscle tone, behavior problems and diffuse learning challenges. Almost inevitably, as a first assessment measure, British mothers were sent by educators to see psychiatrists or family therapists to resolve perceived family difficulties, and often these interventions took many years before women stepped 'outside the box' to seek an ADHD assessment.

II. <u>Potential harms of an ADHD diagnosis</u>

1. Disempowerment through excuse for problems

a. Decreased responsibility for behaviour, parenting and teaching problems

Damico et al -1995

1. (11) (Notes from science class. Teacher taking up homework, walking down aisles, and taking it from each student.) Mike's turn comes, he looks at the teacher and says, "I couldn't do it. It took too much time because of my ADD." 'Teacher frowns and responds, 'That's the third time you've told me that. That ADD is no excuse for not doing your work. You can fail just the same." [Field notes on M.N.; 10-21-921]
2. The physicians interviewed for this study did not hold the same beliefs about the objectivity of the diagnosis of ADHD. One physician, a pediatrician, expressed some of her concerns during the interview:
3. (17) "I feel a real burden at times about the whole ADD business. Parents are starting to come to me looking for the diagnosis. They're almost shoppers in search of that label as an explanation or even an excuse . . . and I'm uncomfortable with it. See, it's too easy to look at behaviors and then make a quick judgment of ADD . . . but I'm not certain we're always right.

© 2021 Kazda L et al. *JAMA Network Open.*

It's so subjective. . . . And then the parents want medication. I'll tell you, I would never be so eager to medicate my children-not in the same way that so many of these parents are doing." [Interview; K.T. 9-10-921

4. This confirmatory reaction has a more subtle implication, one that was expressed by one of the physicians during the interview phase (see example 17): If there is a real problem, medical or biological in nature, then the behaviors and difficulties that the parents have had to address have an explanation. That means that the problems are not due to "character flaws" or "poor parenting" or even "poor teaching" but, rather, to that problem within the child.
5. In effect, as Reid, Maag, and Vasa (1994) have suggested, the child, parents, and teachers are given a "no fault label".

Hamed et al -2015

1. Additionally, others have reported feeling that ADHD is viewed by others as a "convenient excuse" for their behavioral problems and they may be labeled as a "problem person" rather than a person who has a problem (79, 80)

Malacrida –2004

1. Mothers were told things like, 'oh, you don't want that [the diagnosis] to go on his record', or, 'if we label him, it will follow him wherever he goes' or quite simply that 'ADHD is just a label to excuse bad behaviour, and it won't be doing him any favours if we just slap a label on him'.

Moore -2017

1. Practitioners gave examples of how the ADHD label can be used as an excuse by students in the classroom:

   Sam came in last year when he was what, 9 or 10, said 'it's alright I don't have to do that I've got ADHD' (Kate, Teaching Assistant, Primary).

   And this may be encouraged at home: You do find that with the families ... it's an excuse, then the family come in and 'he's got ADHD so that it explains it all' and it's kinda like no it don't really explain it all there's more to it than just a label (Monica, Teacher, PRU).

   Participants saw that the diagnosis removes blame from the child, 'almost validates the behaviour and gives them a reason for it' (Paula, Teacher, PRU); placing the responsibility for behaviour elsewhere was not seen as entirely helpful

Singh -2011

1. Playing up the stigma of ADHD diagnosis is a double-edged sword: when used for prosocial ends it is a positive form of agency; when used for selfish ends, it ultimately diminishes agency. UK children report exploiting their ADHD diagnosis, primarily as an excuse for bad behavior:

   I don't get punished for nothing. It's easy to get away after fights because I have ADHD. I just make puppy eyes and it gets me round everything with my teachers. Alan, age 10

   Unlike US children, who rarely admit to using ADHD as an excuse for their behavior (because they believe it is wrong, but also because niche dynamics strongly encourage them to keep their diagnosis a secret), almost all UK children say they have used ADHD as an excuse. Frequently, it works, at least to a degree.
2. Children report that school personnel tell other children to stay away from them because they have ADHD, and they give lesser punishments to students with ADHD diagnoses.

© 2021 Kazda L et al. *JAMA Network Open.*

3. the more ADHD is socially available as an excuse for behaviors, the less control a child with ADHD has over how he is seen, and indeed, how he sees himself. Even well-meaning friends threaten a child's capacity for self-determination when they use ADHD as an excuse on his behalf. Exploiting ADHD fuels the short fuse stereotype

b. Increased deflection from underlying social, systemic or individual problems

Allan et al -2014

1. ...profound consequences of medicalisation is that it can obscure other interpretations for understanding the behaviour of children from disadvantaged communities who are at greater risk of being medicalised
2. The danger is that the problem becomes one of engagement with this classed-medicalised striation and in so doing risks missing other ways of conceptualising the issues involved with child behaviour problems. This brings to mind Schram's (2000) critique of the medicalisation of welfare. In Schram's view, 'poverty can be an important cause of psychological problems, but correcting those psychological conditions will not necessarily correct the poverty that produced those conditions in the first place' (Schram 2000, 92). In a similar manner, we contend that applying diagnoses to school and classroom-based problems does not correct the causes either.
3. This pursuit of diagnosis by impoverished people, driven by economic need, highlights the problems that can be caused by the medicalisation of poverty (Schram 2000).
4. the extent to which child behaviour is territorialised renders it difficult for other contexts and explanations to be pursued. In a territorialised landscape, the child behaviour assemblage might be seen in a bipolar way where non-diagnosis could come to imply fault.

Damico et al -1995

1. Because of the medical model, we are too often willing to localize academic and behavioral problems solely within the individual child rather than looking for contributing factors in the larger contexts of the child's experience.
2. This societal bias can lead the clinician to decontextualize observed behaviors and attempt to locate the deficits or difficulties within the child's cognitive or neurological system rather than to consider the complex array of variables and behaviors that make up the child's social, educational, and learning contexts.

Klasen -2000

1. The label just distracts from the issue, externalizes the problem. It does not particularly empower the child but tends to disempower it.
2. The danger is that the children might be labeled forever, even if they are not hyperactive. Maybe they have a different problem, like learning disability, which might be less socially acceptable.

c. No meaningful benefit from diagnosis/ no change apart from label

Andersson Frondelius et al -2019

1. The adolescents in this study put substantial hope for change in being diagnosed. The diagnosis was expected to change their life, but their hope was not always fulfilled.

Hamed et al -2015

© 2021 Kazda L et al. *JAMA Network Open.*

1. Despite struggles related to ADHD symptoms, receiving the diagnosis does not always reduce stress for an individual.

Malacrida –2004

1. In her story, and indeed in others like it, educational specialists, administrators and sometimes even teachers, despite medical and psychiatric assessments of ADHD, remained firmly unconvinced of the medicalized status of ADHD, thus making the administration of any kind of non-disciplinary treatment, including medication, problematic for diagnosed children.
2. Repeatedly mothers in both Canada and the UK expressed dismay that teachers seemed reluctant to 'own' the ADD/ADHD problem; they complained that teachers seemingly had little understanding and little interest in understanding or responding to their children's conditions, particularly once the label had been applied and treatment had been prescribed.

Moore -2017

1. However, participants noted that a diagnosis alone is 'Not a wand that can be waved' (Maisy, SENCo, Primary)

Wienen et al -2019

1. 'You may have that label, but in effect, absolutely nothing at all has in fact been changed'. Likewise, respondent 1 commented as follows: 'So, suppose a teacher finds that troublesome. So they want to stick a label on it. Because that tells them what to do. While I then think, really, the label tells me nothing more and nothing less.
2. Ten out of 30 teachers in the data set suggest that an ADHD classification offers no real benefits for educational practice. An example of this is respondent 1: 'I do try to translate it into an educational need, and a label achieves nothing more in those cases. Because you are still, even if a child has a label ADHD, what do you need from me?' Respondent 13 voiced similar concerns: 'But moreover I think, so the child now has a label, so what? I mean, I knew that already, surely? What adjustments do I need to make for him, and I don't think he'll be feeling any better just because there's a label on it.'
3. Two out of the 30 respondents point out the misunderstanding that an ADHD classification ensures that financial means flow to the school. As respondent 4 put it: 'you used to just be able to get money with a label. . .but that is no longer the case now.'

2. Disempowerment through loss of control

a. Diagnosis as first step to behaviour correction, control and manipulation by others

Comstock -2015

1. It is clear from these posts, however, that AD/HD is still sometimes, if not frequently, used as a form of overt behavior control and correction and that AD/HD is not always a positive identity for individuals but a label given to the powerless (such as children) by those with power (such as parents), from the "top down."

Damico et al -1995

1. the physicians are way too willing to label a child as ADD and then medicate them. Some teachers aren't much better. They see what Ritalin did to another kid-only focusing on the kid's disruptive behavior-but they like it . . . it makes the classroom calmer so they recommend

© 2021 Kazda L et al. *JAMA Network Open.*

medication-they can even suggest the doctor to see. Everyone knows who really pushes the drugs." [Interview; K.N. 9-13-92]

2. Each stated that he or she wasn't certain that the medication benefitted the child's learning capabilities, but that it did make the classroom a better place for the other children to learn.

Hamed et al -2015

1. Some perceived their identity was challenged by a diagnosis of ADHD and also felt less in-control of their lives, especially when faced with the prospect of taking medication for life (32).

Malacrida –2004

1. researchers have speculated that the inability to exercise discipline through student exclusion has led US educators to embrace ADD as a medical category and to use Ritalin as a 'substitute' for educational discipline strategies (Kiger, 1985). There is some support for this argument in the stories mothers tell about their children in this study. Canadian mothers, whose children were far less likely to have been suspended or expelled from school than the British children in the study, reported a far higher level of acceptance by educators of drug therapy than was evidenced by British educators. In part, this may stem from the relative lack of alternatives available to Canadian educators than their British counterparts in exercising classroom discipline.

b. Increased passiveness and hopelessness

Klasen -2000

1. Like parents of children with other chronic problems, some parents initially reacted with grief, denial, frustration, or anger. "I found it very hard to accept the diagnosis because it means there is something wrong with him that you have to accept and for which there is no cure. Before I thought it was something that could be fixed. I want him to be well adjusted, I want him to be in a mainstream school, I want him to be happy. You have no idea how it hurts; it just takes my life out. I want him to lead a normal life. I suffer because he suffers. It was a shock to accept that your child has a problem and there is something wrong with him. . . . I was upset, but it was a relief as well to know it wasn't down to me, to things I had been doing or not been doing."
2. Despite these mainly positive comments, however, parents emphasized that the diagnosis was also problematic: it made them realize that they would have to live with a chronically difficult child.
3. The GPs also seemed to think that once people see their problems as medical, they stop working toward improvement. Thus, the doctors' decision to withhold a medical label seems to have been based on the fear that the diagnosis would decrease the children's chances of recovery.
4. fear that medicalization can be disabling, making patients passive and dependent

Moore -2017

1. Practitioners recognize the process of labelling occurring for children with ADHD. While the label may aid understanding and access to support, the negative aspects of labelling 'can sometimes just compound them into their difficulties, rather than pull them out' (Ryan, Pastoral Leader, Primary)

Wienen et al -2019

© 2021 Kazda L et al. *JAMA Network Open.*

1. Respondent 22 put it more generally: 'I think that once parents know that my child has ADHD, it may perhaps remove a bit of uncertainty, like well, he's got it, he is like that, nothing we can do about that.'

c. Self-fulfilling prophecy: perceived inability to change or achieve (by self or others) leads to exclusion and reduced opportunities

Klasen -2000

1. Their other fear was that the child might overidentify with the diagnosis, so that the problem would turn into a self-fulfilling prophecy.
2. Generally, these doctors were very aware of the dangers of medicalization, which they frequently expressed in sociological terms. "I don't want to stigmatize a child unless it has some benefit. The danger is you are medicalizing something that perhaps should not be medicalized. One of the problems is that one might create a self-fulfilling prophecy."

Rogalin et al -2015

1. Participants show awareness of the risks that assigning the psychiatric diagnosis might limit the possibilities of the children's identity. As they report, the diagnosis becomes an obstacle to change as it invites the education staff to place the children with ADHD in the prototypical box. The children are no longer seen as potentially able but with some particular difficulties but rather as children who they know in advance are unable. In other words, if the child has a disorder, than it is natural that he or she is unable to do certain things.
2. The diagnosis becomes hard to get rid of, just like a criminal record, a label that informs others of who the child has been and still is. I think you can get a little "locked" in a certain idea of the child. (Leisure time teacher) If you do not have a diagnosis, one can say: ok, this child has some difficulties with this and this so let us help him with these things. If you have a diagnosis then they are not able to learn this and that, because they have this diagnosis and then they have these difficulties. (Leisure time teacher) It feels like a criminal record, you always have that label that you have committed that crime or that you always have had ADHD. (Teacher)
3. The diagnosed child is the one that is unable to do something, that lacks in abilities. This use of the diagnostic label recalls the reality of a handicap, of a person being unable to do something.
4. Because the diagnosis establishes that a child presents certain behaviors and not others, it lays the ground for the construction of a prototype constituted by expectations on the abilities and behaviors of who has been assigned the diagnosis. When a person is seen by others through the prototype- lenses it will also have impact on what actions are retained as adequate when interacting with the person under consideration (Gergen 1997a, b, 1999, 2009; Gergen and Gergen 1993). Much like the expression "anticipated others" (Goffman 1963,p. 12) indicates, the prototype consents anticipation of future behaviors and precautions can thus be made even before behaviors occur in order to avoid situations experienced as critical. Consequently, if someone is expected not to perform well at certain tasks or in certain academic or occupational areas (APA 2000) the person under consideration might be excluded from these tasks and areas in advance
5. As hypothesized by other authors (e.g. Levine 1997) a diagnostic label promotes the search and confirmation of similarities. It is precisely for this reason that it becomes important to possess as much knowledge as possible about a diagnosis. It becomes important because the knowledge is not neutral, it explains why the children with the diagnosis behave as they do and it helps to know what to do when these children react or say something and even how to

© 2021 Kazda L et al. *JAMA Network Open.*

intervene in advance in terms of precaution avoiding that anticipated unwanted situations occur. As a consequence, the identity of a child that has been assigned a diagnostic label is limited. Who the children are and what they can or cannot do depend, at least in part, of the opportunities that are given to them. If certain tasks/situations are avoided for the children with the diagnosis they are excluded from the opportunity to be able in those tasks/situations and on the contrary certain inabilities will become a part of their identity as they are interpreted as an outcome of the diagnosis thus as a natural part of who they are.

6. With the knowledge of a child having a diagnosis however, efforts to improve abilities or change the ways in which a child relate to a peer might still be made but it becomes easy to write off an inability to solve a task or fighting with a peer as a symptom of the disorder and therefore leave it to be. After all, if the children's behaviors are caused by a disorder, than it is only natural that they are unable to do certain things and that they behave in a certain way

3. Stigmatisation through permanent label/ identity

a. Creates an identity which enhances prejudice, stereotypes, judgement

Andersson Frondelius et al -2019

1. The participants described that the label 'ADHD' could mean different things, depending on the context. It could be used as a condescending stamp, but it was also a facilitator to professional help.

DosReis et al -2010

1. Another concern was that as a consequence of being labeled, their child would be treated differently or not given the same opportunities as their peers.

Hamed et al -2015

1. Some individuals have described perceiving a certain degree of stigma attached to being diagnosed with ADHD, influencing their willingness to disclose their diagnosis to other people.
2. At times, ADHD diagnosis led young people to feel hurt by their peers when they were teased or targeted because of their apparent academic delay and labeled as "retarded" (79).

Klasen -2000

1. A small number of parents feared that the label might bring disadvantages for their child. They were particularly worried about the stigma attached, which might lead to problems at school.
2. Generally, these doctors were very aware of the dangers of medicalization, which they frequently expressed in sociological terms. "I don't want to stigmatize a child unless it has some benefit. The danger is you are medicalizing something that perhaps should not be medicalized. One of the problems is that one might create a self-fulfilling prophecy."
3. A label can be very frustrating as it makes the kid stand out. It can also lead to scapegoating by increasing the conflict between parent and child.

Moore -2017

1. In the current study, some practitioners 'don't think it's nice to have that [ADHD] label' (Bryony, SENCo, PRU) because 'so many people are … stigmatized by these sorts of things' (Tarquin, Teacher, PRU). One participant pointed out that other learning difficulties like dyslexia are more 'socially acceptable' (Janet, Teacher, Secondary) than ADHD.

© 2021 Kazda L et al. *JAMA Network Open.*

Rogalin et al -2015

1. You don't take it just as any child. There is something that tells you "this is what a child with ADHD is". That they are messy, that they have problems with concentration, loud, that they take up space. That there are going to be problems. (Leisure time teacher)
2. On one hand we have the parts of the accounts that form an idea of a typical ADHD-child with typical behaviors. As a consequence of this idea, certain expectations are placed on the child whose performances and future behaviors are anticipated.
3. Participants show awareness of the risks that assigning the psychiatric diagnosis might limit the possibilities of the children's identity. As they report, the diagnosis becomes an obstacle to change as it invites the education staff to place the children with ADHD in the prototypical box. The children are no longer seen as potentially able but with some particular difficulties but rather as children who they know in advance are unable. In other words, if the child has a disorder, than it is natural that he or she is unable to do certain things.

Singh -2011

1. Children report that school personnel tell other children to stay away from them because they have ADHD, and they give lesser punishments to students with ADHD diagnoses.

Wienen et al -2019

1. The main disadvantage mentioned is that an ADHD classification bears down on a child for many years.

b. Increases feelings of isolation, exclusion and shame

Hamed et al -2015

1. Still other individuals report having the diagnosis of ADHD lead them to feel as though they were different and isolated (79).

Moore -2017

1. Another participant spoke of 'the shame' (Bryony, SENCo, PRU) surrounding ADHD and how this can lead students to be reluctant to ask for or accept help.

Singh -2011

1. Children report that school personnel tell other children to stay away from them because they have ADHD, and they give lesser punishments to students with ADHD diagnoses.

© 2021 Kazda L et al. *JAMA Network Open.*

eAppendix 5. Critical Appraisal Results

Author	Year	Overall appraisal
Akinbami, L. J.; Liu, X.; Pastor, P. N.; Reuben, C. A.	2011	High Risk of Bias
Akmatov, M. K.; Steffen, A.; Holstiege, J.; Hering, R.; Schulz, M.; Batzing, J.	2018	Low Risk of Bias
Alessi-Severini, S.; Biscontri, R. G.; Collins, D. M.; Sareen, J.; Enns, M. W.	2012	Low Risk of Bias
Allan, J.; Harwood, V.	2014	High Risk of Bias
Anderson, J.	2001	Moderate Risk of Bias
Andersson Frondelius, I.; V. Ranjbar; L. Danielsson	2019	Low Risk of Bias
Angold, A.; Erkanli, A.; Egger, H. L.; Costello, E. J.	2000	Moderate Risk of Bias
Arnold, L. E.; Hodgkins, P.; Caci, H.; Kahle, J.; Young, S.	2015	High Risk of Bias
Atladottir, H.O.; D. Gyllenberg; A. Langridge; S. Sandin; S. N. Hansen; H. Leonard; M. Gissler; A. Reichenberg; D. E. Schendel; J. Bourke; C. M. Hultman; D. E. Grice; J. D. Buxbaum; E. T. Parner	2015	Moderate Risk of Bias
Bachmann, C. J.; Wijlaars, L. P.; Kalverdijk, L. J.; Burcu, M.; Glaeske, G.; Schuiling-Veninga, C. C. M.; Hoffmann, F.; Aagaard, L.; Zito, J. M.	2017	Moderate Risk of Bias
Balazs, J.; Kereszteny, A.	2014	High Risk of Bias
Barbaresi, W. J.; Katusic, S. K.; Colligan, R. C.; Weaver, A. L.; Jacobsen, S. J.	2007	High Risk of Bias
Barczyk, Z. A.; J. J. Rucklidge; M. Eggleston; R. T. Mulder	2020	Low Risk of Bias
Barkley, R. A.; Fischer, M.; Smallish, L.; Fletcher, K.	2003	High Risk of Bias
Bastiaens, L.	2011	High Risk of Bias
Batzle, C. S.; Weyandt, L. L.; Janusis, G. M.; DeVietti, T. L.	2010	High Risk of Bias
Baumgaertel, A.; Wolraich, M. L.; Dietrich, M.	1995	Moderate Risk of Bias
Bax, A. C.; Bard, D. E.; Cuffe, S. P.; McKeown, R. E.; Wolraich, M. L.	2019	Moderate Risk of Bias
Beau-Lejdstrom, R.; Douglas, I.; Evans, S. J. W.; Smeeth, L.	2016	Moderate Risk of Bias
Benner-Davis, S.; Heaton, P. C.	2007	High Risk of Bias
Biederman, J.; Fitzgerald, M.; Kirova, A. M.; Woodworth, K. Y.; Biederman, I.; Faraone, S. V.	2018	High Risk of Bias
Biederman, J.; Monuteaux, M. C.; Spencer, T.; Wilens, T. E.; Macpherson, H. A.; Faraone, S. V.	2008	Moderate Risk of Bias
Biederman, J.; Wilens, T.; Mick, E.; Spencer, T.; Faraone, S. V.	1999	High Risk of Bias
Bjerkeli, P. J.; Vicente, R. P.; Mulinari, S.; Johnell, K.; Merlo, J.	2018	Low Risk of Bias
Boland, F.; Galvin, R.; Reulbach, U.; Motterlini, N.; Kelly, D.; Bennett, K.; Fahey, T.	2015	Low Risk of Bias
Boland, H; M. DiSalvo; R. Fried; K. Y. Woodworth; T. Wilens; S. V. Faraone; J. Biederman	2020	High Risk of Bias

© 2021 Kazda L et al. *JAMA Network Open.*

Bonati, M.; Cartabia, M.; Zanetti, M.; Reale, L.; Didoni, A.; Costantino, M. A.; Conte, S.; Renzetti, V.; Salvoni, L.; Molteni, M.; Trabattoni, S.; Effedri, P.; Fazzi, E.; Filippini, E.; Pedercini, E.; Zanetti, E.; Fteita, N.; Arisi, D.; Mapelli, R.; Frassica, S.; Oriani, S.; Trevisan, C.; Acquistapace, S.; Martinelli, O.; Villani, D.; Binaghi, E.; Deriu, A.; Vasile, G.; Borchia, A.; Morosini, P.; Breviglieri, M.; Capovilla, G.; Segala, R.; Battaini, C.; Bissoli, C.; Canevini, M. P.; Cropanese, I.; Fornaro, E.; Leonardi, G.; Merati, S.; Saccani, M.; Vaccari, R.; Valenti, V.; Balottin, U.; Chiappedi, M.; Vlacos, E.; Meraviglia, C.; Palmieri, M. G.; Ruffoni, G.; Rinaldi, F.; Soardi, F.; Luoni, C.; Rossi, G.	2018	Low Risk of Bias
Bowling, A.; Davison, K.; Haneuse, S.; Beardslee, W.; Miller, D. P.	2017	Low Risk of Bias
Brault, M. C.; Lacourse, E.	2012	Low Risk of Bias
Brossard-Racine, M.; Shevell, M.; Snider, L.; Belanger, S. A.; Majnemer, A.	2012	High Risk of Bias
Brownell, M. D.; Yogendran, M. S.	2001	Low Risk of Bias
Bruchmuller, K.; Margraf, J.; Schneider, S.	2012	Moderate Risk of Bias
Bruckner, T. A.; Hodgson, A.; Mahoney, C. B.; Fulton, B. D.; Levine, P.; Scheffler, R. M.	2012	Moderate Risk of Bias
Burcu, M.; J. Zito; L. Metcalfe	2016	Moderate Risk of Bias
Butte, N. F.; Treuth, M. S.; Voigt, R. G.; Llorente, A. M.; Heird, W. C.	1999	High Risk of Bias
Carr-Fanning, K.; Mc Guckin, C.	2018	High Risk of Bias
Castle, L.; Aubert, R. E.; Verbrugge, R. R.; Khalid, M.; Epstein, R. S.	2007	Moderate Risk of Bias
Catala-Lopez, F.; Hutton, B.; Nunez-Beltran, A.; Page, M. J.; Ridao, M.; Macias Saint-Gerons, D.; Catala, M. A.; Tabares-Seisdedos, R.; Moher, D.	2017	Low Risk of Bias
Centers for Disease, Control; Prevention	2005	Low Risk of Bias
Centers for Disease, Control; Prevention	2010	Low Risk of Bias
Chai, G.; Governale, L.; McMahon, A. W.; Trinidad, J. P.; Staffa, J.; Murphy, D.	2012	Moderate Risk of Bias
Chang, Z.; Ghirardi, L.; Quinn, P. D.; Asherson, P.; D'Onofrio, B. M.; Larsson, H.	2019	High Risk of Bias
Chang, Z; P. D. Quinn; L. O'Reilly; A. Sjolander; K. Hur; R. Gibbons; H. Larsson; B. M. D'Onofrio	2019	Low Risk of Bias
Charach, A.; Ickowicz, A.; Schachar, R.	2004	High Risk of Bias
Charles, L.; Schain, R.	1981	High Risk of Bias
Chen, Q.; Sjolander, A.; Runeson, B.; D'Onofrio, B. M.; Lichtenstein, P.; Larsson, H.	2014	Low Risk of Bias
Cheng, J. Y.; Chen, R. Y.; Ko, J. S.; Ng, E. M.	2007	Low Risk of Bias
Chien, I. C.; Lin, C. H.; Chou, Y. J.; Chou, P.	2012	Low Risk of Bias
Ching, C.; Eslick, G. D.; Poulton, A. S.	2019	High Risk of Bias
Chirdkiatgumchai, V.; Xiao, H.; Fredstrom, B. K.; Adams, R. E.; Epstein, J. N.; Shah, S. S.; Brinkman, W. B.; Kahn, R. S.; Froehlich, T. E.	2013	Moderate Risk of Bias

© 2021 Kazda L et al. *JAMA Network Open.*

Cho, S. C.; Kim, B. N.; Kim, J. W.; Rohde, L. A.; Hwang, J. W.; Chungh, D. S.; Shin, M. S.; Lyoo, I. K.; Go, B. J.; Lee, S. E.; Kim, H. W.	2009	Low Risk of Bias
Clavenna, A.; Bonati, M.	2014	High Risk of Bias
Coghill, D.	2010	High Risk of Bias
Coghill, D. R.; Banaschewski, T.; Soutullo, C.; Cottingham, M. G.; Zuddas, A.	2017	High Risk of Bias
Coghill, D. R.; Seth, S.; Pedroso, S.; Usala, T.; Currie, J.; Gagliano, A.	2014	High Risk of Bias
Coker, T. R.; Elliott, M. N.; Toomey, S. L.; Schwebel, D. C.; Cuccaro, P.; Emery, S. T.; Davies, S. L.; Visser, S. N.; Schuster, M. A.	2016	Moderate Risk of Bias
Coleman, Daniel; Walker, Janet S.; Lee, Junghee; Friesen, Barbara J.; Squire, Peter N.	2009	High Risk of Bias
Collins, K. P.; Cleary, S. D.	2016	Moderate Risk of Bias
Comstock, Edward	2015	Low Risk of Bias
Connor, D. F.	2002	High Risk of Bias
Cooper, W. O.; L. A. Habel; C. M. Sox; K. A. Chan; P. G. Arbogast; T. C. Cheetham; K. T. Murray; V. P. Quinn; C. M. Stein; S. T. Callahan; B. H. Fireman; F. A. Fish; H. S. Kirshner; A. O'Duffy; F. A. Connell; W. A. Ray	2011	Low Risk of Bias
Cornett-Ruiz, Stacey; Hendricks, Bryan	1993	High Risk of Bias
Cortese, S.; N. Adamo; C. Del Giovane; C. Mohr-Jensen; A. J. Hayes; S. Carucci; L. Z. Atkinson; L. Tessari; T. Banaschewski; D. Coghill; C. Hollis; E. Simonoff; A. Zuddas; C. Barbui; M. Purgato; H. C. Steinhausen; F. Shokraneh; J. Xia; A. Cipriani	2018	Moderate Risk of Bias
Cuffe, S. P.; Moore, C. G.; McKeown, R. E.	2005	Moderate Risk of Bias
Currie, J.; Stabile, M.; Jones, L.	2014	Moderate Risk of Bias
Dalsgaard, S.; A. P. Kvist; J. F. Leckman; H. S. Nielsen; M. Simonsen	2014	Low Risk of Bias
Dalsgaard, S.; Leckman, J. F.; Mortensen, P. B.; Nielsen, H. S.; Simonsen, M.	2015	Low Risk of Bias
Dalsgaard, S.; M. K. Humlum; H. S. Nielsen; M. Simonsen	2012	Low Risk of Bias
Dalsgaard, S.; Nielsen, H. S.; Simonsen, M.	2014	Low Risk of Bias
Dalsgaard, S.; Nielsen, H. S.; Simonsen, M.	2013	Low Risk of Bias
Damico, J. S.; Augustine, L. E.	1995	Low Risk of Bias
Danielson, M. L.; Visser, S. N.; Gleason, M. M.; Peacock, G.; Claussen, A. H.; Blumberg, S. J.	2017	Moderate Risk of Bias
Davidovitch, M.; Koren, G.; Fund, N.; Shrem, M.; Porath, A.	2017	Low Risk of Bias
Davies, M.; Coughtrie, A.; Layton, D.; Shakir, S. A.	2017	High Risk of Bias
Davis, D. W.; Feygin, Y.; Creel, L.; Williams, P. G.; Lohr, W. D.; Jones, V. F.; Le, J.; Pasquenza, N.; Ghosal, S.; Jawad, K.; Yan, X.; Liu, G.; McKinley, S.	2019	Low Risk of Bias
Didoni, A.; Sequi, M.; Panei, P.; Bonati, M.	2011	Moderate Risk of Bias
Diez-Suarez, A.; Vallejo-Valdivielso, M.; Marin-Mendez, J. J.; De Castro-Manglano, P.; Soutullo, C. A.	2017	Moderate Risk of Bias

© 2021 Kazda L et al. *JAMA Network Open.*

Donfrancesco, R.; Marano, A.; Calderoni, D.; Mugnaini, D.; Thomas, F.; Di Trani, M.; Innocenzi, M.; Vitiello, B.	2015	Moderate Risk of Bias
Dopfner, M.; Breuer, D.; Wille, N.; Erhart, M.; Ravens-Sieberer, U.	2008	Moderate Risk of Bias
DosReis, S.; Barksdale, C. L.; Sherman, A.; Maloney, K.; Charach, A.	2010	High Risk of Bias
Dryer, R.; Kiernan, M. J.; Tyson, G. A.	2006	High Risk of Bias
Elder, T.E.	2010	Moderate Risk of Bias
Ercan, E. S.; Kose, S.; Kutlu, A.; Akyol, O.; Durak, S.; Aydin, C.	2012	High Risk of Bias
Evans, W. N.; Morrill, M. S.; Parente, S. T.	2010	Low Risk of Bias
Fabiano, F; N. Haslam	2020	Moderate Risk of Bias
Fabiano, Gregory A.; Pelham, William E., Jr.; Majumdar, Antara; Evans, Steven W.; Manos, Michael J.; Caserta, Donald; Girio-Herrera, Erin L.; Pisecco, Stewart; Hannah, Jane N.; Carter, Randy L.	2013	High Risk of Bias
Fairman, K.A.; A. M. Peckham; D. A. Sclar	2020	Low Risk of Bias
Faraone, S. V.; Biederman, J.; Morley, C. P.; Spencer, T. J.	2008	High Risk of Bias
Fergusson, D. M.; Lynskey, M. T.; Horwood, L. J.	1997	Low Risk of Bias
Fergusson, David M.; Boden, Joseph M.; Horwood, L.	2010	Low Risk of Bias
Fergusson, David M.; Horwood, L.	1995	Moderate Risk of Bias
Fleming, M.; Fitton, C. A.; Steiner, M. F. C.; McLay, J. S.; Clark, D.; King, A.; Mackay, D. F.; Pell, J. P.	2017	Moderate Risk of Bias
Fogelman, Y.; Vinker, S.; Guy, N.; Kahan, E.	2003	Low Risk of Bias
Foreman, David M.; Ford, Tamsin	2008	Moderate Risk of Bias
Frank, E.; Ozon, C.; Nair, V.; Othee, K.	2015	High Risk of Bias
Froehlich, T. E.; Lanphear, B. P.; Epstein, J. N.; Barbaresi, W. J.; Katusic, S. K.; Kahn, R. S.	2007	Moderate Risk of Bias
Fullerton, C. A.; Epstein, A. M.; Frank, R. G.; Normand, S. L.; Fu, C. X.; McGuire, T. G.	2012	Low Risk of Bias
Fulton, B. D.; Scheffler, R. M.; Hinshaw, S. P.	2015	Moderate Risk of Bias
Furu, K.; Karlstad, O.; Zoega, H.; Martikainen, J. E.; Bahmanyar, S.; Kieler, H.; Pottegard, A.	2017	Low Risk of Bias
Gajria, K.; Lu, M.; Sikirica, V.; Greven, P.; Zhong, Y.; Qin, P.; Xie, J.	2014	High Risk of Bias
Garfield, C. F.; Dorsey, E. R.; Zhu, S.; Huskamp, H. A.; Conti, R.; Dusetzina, S. B.; Higashi, A.; Perrin, J. M.; Kornfield, R.; Alexander, G. C.	2012	Moderate Risk of Bias
Gayleard, J. L.; Mychailyszyn, M. P.	2017	Moderate Risk of Bias
Getahun, D.; Jacobsen, S. J.; Fassett, M. J.; Chen, W.; Demissie, K.; Rhoads, G. G.	2013	Low Risk of Bias
Ghanizadeh, A.	2013	High Risk of Bias
Ghanizadeh, A.; Fallahi, M.; Akhondzadeh, S.	2009	High Risk of Bias
Ghanizadeh, A.; Freeman, R. D.; Berk, M.	2013	High Risk of Bias
Giacobini, M.; Medin, E.; Ahnemark, E.; Russo, L. J.; Carlqvist, P.	2018	Low Risk of Bias

© 2021 Kazda L et al. *JAMA Network Open.*

Gibbs, S.; J. F. Beckmann; J. Elliott; R. L. Metsapelto; T. Vehkakoski; M. Aro	2020	High Risk of Bias
Girand, H. L.; S. Litkowiec; M. Sohn	2020	Low Risk of Bias
Goetz, M.; Yeh, C. B.; Ondrejka, I.; Akay, A.; Herczeg, I.; Dobrescu, I.; Kim, B. N.; Jin, X.; Riley, A. W.; Martenyi, F.; Harrison, G.; Treuer, T.	2012	High Risk of Bias
Grimmsmann, T.; W. Himmel	2020	High Risk of Bias
Groenman, A. P.; Oosterlaan, J.; Rommelse, N. N.; Franke, B.; Greven, C. U.; Hoekstra, P. J.; Hartman, C. A.; Luman, M.; Roeyers, H.; Oades, R. D.; Sergeant, J. A.; Buitelaar, J. K.; Faraone, S. V.	2013	High Risk of Bias
Gumy, C.; Huissoud, T.; Dubois-Arber, F.	2010	Low Risk of Bias
Habel, L. A.; Schaefer, C. A.; Levine, P.; Bhat, A. K.; Elliott, G.	2005	Low Risk of Bias
Halldner, Linda; Tillander, Annika; Lundholm, Cecilia; Boman, Marcus; Langstrom, Niklas; Larsson, Henrik; Lichtenstein, Paul	2014	Low Risk of Bias
Hamed, A. M.; Kauer, A. J.; Stevens, H. E.	2015	High Risk of Bias
Harstad, E. B.; Weaver, A. L.; Katusic, S. K.; Colligan, R. C.; Kumar, S.; Chan, E.; Voigt, R. G.; Barbaresi, W. J.	2014	Moderate Risk of Bias
Haslam, N.; Williams, B.; Prior, M.; Haslam, R.; Graetz, B.; Sawyer, M.	2006	Low Risk of Bias
Havey, J.; Olson, Julie M.; McCormick, Christine; Cates, Gary L.	2005	High Risk of Bias
Hire, A. J.; Ashcroft, D. M.; Springate, D. A.; Steinke, D. T.	2018	Moderate Risk of Bias
Hirota, T.; Schwartz, S.; Correll, C. U.	2014	Moderate Risk of Bias
Hoagwood, K. E.; Kelleher, K.; Zima, B. T.; Perrin, J. M.; Bilder, S.; Crystal, S.	2016	Low Risk of Bias
Hodgkins, P.; Sasane, R.; Meijer, W. M.	2011	Low Risk of Bias
Holden, S. E.; Jenkins-Jones, S.; Poole, C. D.; Morgan, C. L.; Coghill, D.; Currie, C. J.	2013	Moderate Risk of Bias
Holland, J.; Sayal, K.	2018	Moderate Risk of Bias
Hollingworth, S. A.; Nissen, L. M.; Stathis, S. S.; Siskind, D. J.; Varghese, J. M. N.; Scott, J. G.	2011	Low Risk of Bias
Holmskov, M.; Storebo, O. J.; Moreira-Maia, C. R.; Ramstad, E.; Magnusson, F. L.; Krogh, H. B.; Groth, C.; Gillies, D.; Zwi, M.; Skoog, M.; Gluud, C.; Simonsen, E.	2017	Low Risk of Bias
Hong, S. B.; Dwyer, D.; Kim, J. W.; Park, E. J.; Shin, M. S.; Kim, B. N.; Yoo, H. J.; Cho, I. H.; Bhang, S. Y.; Hong, Y. C.; Pantelis, C.; Cho, S. C.	2014	High Risk of Bias
Huang, C. L.; Chu, C. C.; Cheng, T. J.; Weng, S. F.	2014	Low Risk of Bias
Huang, C.L.; J. J. Wang; C. H. Ho	2020	Low Risk of Bias
Hudziak, J. J.; Heath, A. C.; Madden, P. F.; Reich, W.; Bucholz, K. K.; Slutske, W.; Bierut, L. J.; Neuman, R. J.; Todd, R. D.	1998	High Risk of Bias
Hugtenburg, J.; E. Heerdink; A. Egberts	2004	Low Risk of Bias
Humphreys, K. L.; Eng, T.; Lee, S. S.	2013	Moderate Risk of Bias
Huss, M.; Holling, H.; Kurth, B. M.; Schlack, R.	2008	Low Risk of Bias

© 2021 Kazda L et al. *JAMA Network Open.*

Huss, M.; Poustka, F.; Lehmkuhl, G.; Lehmkuhl, U.	2008	Moderate Risk of Bias
Jaber, L.; Rigler, S.; Shuper, A.; Diamond, G.	2017	Moderate Risk of Bias
Jangmo, A.; Stalhandske, A.; Chang, Z.; Chen, Q.; Almqvist, C.; Feldman, I.; Bulik, C. M.; Lichtenstein, P.; D'Onofrio, B.; Kuja-Halkola, R.; Larsson, H.	2019	Low Risk of Bias
Janols, L. O.; Liliemark, J.; Klintberg, K.; von Knorring, A. L.	2009	Moderate Risk of Bias
Jick, H.; Kaye, J. A.; Black, C.	2004	Moderate Risk of Bias
Johansen, M. E.; Matic, K.; McAlearney, A. S.	2015	Low Risk of Bias
Karanges, E. A.; Stephenson, C. P.; McGregor, I. S.	2014	Moderate Risk of Bias
Karlstad, O.; Furu, K.; Stoltenberg, C.; Haberg, S. E.; Bakken, I. J.	2017	Low Risk of Bias
Keen, D.; Hadijikoumi, I.	2011	Moderate Risk of Bias
Keilow, M.; Holm, A.; Fallesen, P.	2018	Moderate Risk of Bias
King, S.; Griffin, S.; Hodges, Z.; Weatherly, H.; Asseburg, C.; Richardson, G.; Golder, S.; Taylor, E.; Drummond, M.; Riemsma, R.	2006	Low Risk of Bias
Kirova, A. M.; Kelberman, C.; Storch, B.; DiSalvo, M.; Woodworth, K. Y.; Faraone, S. V.; Biederman, J.	2019	High Risk of Bias
Klasen, H.	2000	Low Risk of Bias
Klassen, A.; Miller, A.; Raina, P.; Lee, S. K.; Olsen, L.	1999	Moderate Risk of Bias
Knellwolf, A.-L.; J. Deligne; F. Chiarotti; G.-R. Auleley; S. Palmieri; C. Blum Boisgard; E. Autret-Leca	2008	Moderate Risk of Bias
Konrad-Bindl, D. S.; Gresser, U.; Richartz, B. M.	2016	High Risk of Bias
Koonce, Danel A.; Cruce, Michael K.; Aldridge, Jennifer O.; Langford, Courtney A.; Sporer, Amy K.; Stinnett, Terry A.	2004	High Risk of Bias
Kortekaas-Rijlaarsdam, A. F.; Luman, M.; Sonuga-Barke, E.; Oosterlaan, J.	2019	Low Risk of Bias
Lahey, B B; W. E. Pelham; A. Chronis; G. Massetti; H. Kipp; A. Ehrhardt; S. S. Lee	2006	Moderate Risk of Bias
Lahey, B. B.; Applegate, B.; McBurnett, K.; Biederman, J.; Greenhill, L.; Hynd, G. W.; Barkley, R. A.; Newcorn, J.; Jensen, P.; Richters, J.; et al.,	1994	High Risk of Bias
Lahey, B. B.; Loeber, R.; Stouthamer-Loeber, M.; Christ Green, M. A. G. S.; Russo, M. F.; Frick, P. J.; Dulcan, M.	1990	High Risk of Bias
Landgren, M.; Nasic, S.; Johnson, M.; Lovoll, T.; Holmgren, D.; Fernell, E.	2017	High Risk of Bias
Langberg, J. M.; Becker, S. P.	2012	High Risk of Bias
Langner, I.; Haug, U.; Scholle, O.; Lindemann, C.; Schroder, C.; Riedel, O.	2019	Low Risk of Bias
Larsson, H.; Ankcarsater, H.; Rastam, M.; Chang, Z.; Lichtenstein, P.	2012	Low Risk of Bias
Law, G.; Sinclair, Scott; Fraser, Nicole	2007	High Risk of Bias
Layton, T. J.; Barnett, M. L.; Hicks, T. R.; Jena, A. B.	2018	Low Risk of Bias

© 2021 Kazda L et al. *JAMA Network Open.*

Lecendreux, M.; Konofal, E.; Cortese, S.; Faraone, S. V.	2015	High Risk of Bias
Lecendreux, M.; Silverstein, M.; Konofal, E.; Cortese, S.; Faraone, S. V.	2019	High Risk of Bias
Lee, S. I.; Schachar, R. J.; Chen, S. X.; Ornstein, T. J.; Charach, A.; Barr, C.; Ickowicz, A.	2008	High Risk of Bias
Leibson, C. L.; Barbaresi, W. J.; Ransom, J.; Colligan, R. C.; Kemner, J.; Weaver, A. L.; Katusic, S. K.	2006	Low Risk of Bias
Leung, P. W.; S. L. Luk; T. P. Ho; E. Taylor; F. L. Mak; J. Bacon-Shone	1996	Low Risk of Bias
Levine, M.; Froberg, B.; Ruha, A. M.; Burns-Ewald, M.; Yen, M.; Claudius, I. A.; Arthur, A. O.; Tormoehlen, L.; Thomas, S. H.	2013	High Risk of Bias
Levy, F.; Hay, D. A.; McStephen, M.; Wood, C.; Waldman, I.	1997	High Risk of Bias
Liang, S. H.; Yang, Y. H.; Kuo, T. Y.; Liao, Y. T.; Lin, T. C.; Lee, Y.; McIntyre, R. S.; Kelsen, B. A.; Wang, T. N.; Chen, V. C.	2018	Moderate Risk of Bias
Lillemoen, P. K.; Kjosavik, S. R.; Hunskar, S.; Ruths, S.	2012	Low Risk of Bias
Lisska, M. C.; Rivkees, S. A.	2003	High Risk of Bias
Longridge, R.; Norman, S.; Henley, W.; Newlove Delgado, T.; Ford, T.	2019	High Risk of Bias
Lopez-Leon, S.; Lopez-Gomez, M. I.; Warner, B.; Ruiter-Lopez, L.	2018	Low Risk of Bias
Loughran, Sandra B.	2003	High Risk of Bias
Lubke, G. H.; Hudziak, J. J.; Derks, E. M.; van Bijsterveldt, T. C.; Boomsma, D. I.	2009	Moderate Risk of Bias
M. T. A. Cooperative Group	2004	Low Risk of Bias
Madsen, K. B.; Ravn, M. H.; Arnfred, J.; Olsen, J.; Rask, C. U.; Obel, C.	2018	Low Risk of Bias
Maia, C. R.; Cortese, S.; Caye, A.; Deakin, T. K.; Polanczyk, G. V.; Polanczyk, C. A.; Rohde, L. A.	2017	High Risk of Bias
Malacrida, C.	2004	High Risk of Bias
Man, K. K. C.; Coghill, D.; Chan, E. W.; Lau, W. C. Y.; Hollis, C.; Liddle, E.; Banaschewski, T.; McCarthy, S.; Neubert, A.; Sayal, K.; Ip, P.; Schuemie, M. J.; Sturkenboom, Mcjm; Sonuga-Barke, E.; Buitelaar, J.; Carucci, S.; Zuddas, A.; Kovshoff, H.; Garas, P.; Nagy, P.; Inglis, S. K.; Konrad, K.; Hage, A.; Rosenthal, E.; Wong, I. C. K.	2017	Low Risk of Bias
Man, Kenneth K.; Ip, Patrick; Hsia, Yingfen; Chan, Esther W.; Chui, Celine S.; Lam, May P.; Wong, Wilfred H.; Chow, C.; Yung, Ada; Wong, Ian C.	2017	Low Risk of Bias
Maneeton, B.; Maneeton, N.; Likhitsathian, S.; Suttajit, S.; Narkpongphun, A.; Srisurapanont, M.; Woottiluk, P.	2015	Moderate Risk of Bias
Mann, E. M. et al	1992	High Risk of Bias
Mannuzza, S.; Klein, R. G.; Truong, N. L.; Moulton, Iii J. L.; Roizen, E. R.; Howell, K. H.; Castellanos, F. X.	2008	High Risk of Bias
Marcus, D. K.; Barry, T. D.	2011	Low Risk of Bias
McCarthy, S.; Cranswick, N.; Potts, L.; Taylor, E.; Wong, I. C.	2009	Moderate Risk of Bias
McCarthy, S.; Wilton, L.; Murray, M. L.; Hodgkins, P.; Asherson, P.; Wong, I. C.	2012	Low Risk of Bias

© 2021 Kazda L et al. *JAMA Network Open.*

McKeown, R. E.; Holbrook, J. R.; Danielson, M. L.; Cuffe, S. P.; Wolraich, M. L.; Visser, S. N.	2015	Moderate Risk of Bias
McLennan, J. D.	2016	High Risk of Bias
Merrell, Christine; Sayal, Kapil; Tymms, Peter; Kasim, Adetayo	2017	Low Risk of Bias
Metzger, A.N.; L. T. Hamilton	2020	Low Risk of Bias
Meza, J. I.; Monroy, M.; Ma, R.; Mendoza-Denton, R.	2019	High Risk of Bias
Mikolajczyk, R.; Horn, J.; Schmedt, N.; Langner, I.; Lindemann, C.; Garbe, E.	2015	Low Risk of Bias
Moen, M. D.; Keam, S. J.	2009	High Risk of Bias
Mohr Jensen, C.; Steinhausen, H. C.	2015	Low Risk of Bias
Mohr-Jensen, Christina; Bisgaard, Charlotte Muller; Boldsen, Soren Kjaersgaard; Steinhausen, Hans-Christoph	2019	Low Risk of Bias
Molina, B. S.; Hinshaw, S. P.; Swanson, J. M.; Arnold, L. E.; Vitiello, B.; Jensen, P. S.; Epstein, J. N.; Hoza, B.; Hechtman, L.; Abikoff, H. B.; Elliott, G. R.; Greenhill, L. L.; Newcorn, J. H.; Wells, K. C.; Wigal, T.; Gibbons, R. D.; Hur, K.; Houck, P. R.; M. T. A. Cooperative Group	2009	Low Risk of Bias
Moore, D.; Russell, A.; Arnell, S.; Ford, T.	2017	Low Risk of Bias
Moran, L. V., Ongur, D., Hsu, J., Castro, V. M., Perlis, R. H., Schneeweiss, S.	2019	Low Risk of Bias
Morgan, P. L.; J. Staff; M. M. Hillemeier; G. Farkas; S. Maczuga	2013	Moderate Risk of Bias
Morkem, R.; Patten, S.; Queenan, J.; Barber, D.	2017	High Risk of Bias
Morley, C. P.	2010	Moderate Risk of Bias
Morrow, Richard L.; Garland, E.; Wright, James M.; Maclure, Malcolm; Taylor, Suzanne; Dormuth, Colin R.	2012	Low Risk of Bias
Newcorn, J. H.; Halperin, J. M.; Schwartz, S.; Pascualvaca, D.; Wolf, L.; Schmeidler, J.; Sharma, V.	1994	High Risk of Bias
Newcorn, J.; Halperin, Jeffrey M.; Healey, Jane M.; O'Brien, John D.; Pascualvaca, Daisy M.; Wolf, Lorraine E.; Morganstein, Allen; Sharma, Vanshdeep; Young, J.	1989	High Risk of Bias
Noren Selinus, E.; Molero, Y.; Lichtenstein, P.; Anckarsater, H.; Lundstrom, S.; Bottai, M.; Hellner Gumpert, C.	2016	Low Risk of Bias
Nyarko, K. A.; Grosse, S. D.; Danielson, M. L.; Holbrook, J. R.; Visser, S. N.; Shapira, S. K.	2017	Low Risk of Bias
O'Connor, Briannon; Garner, Annie A.; Peugh, James L.; Simon, John; Epstein, Jeffrey N.	2015	Moderate Risk of Bias
O'Connor, C.; F. McNicholas	2020	Low Risk of Bias
Ohan, J. L.; Visser, T. A.; Moss, R. G.; Allen, N. B.	2013	High Risk of Bias
Ohan, J. L.; Visser, T. A.; Strain, M. C.; Allen, L.	2011	High Risk of Bias
Okumura, Y.; Yamasaki, S.; Ando, S.; Usami, M.; Endo, K.; Hiraiwa-Hasegawa, M.; Kasai, K.; Nishida, A.	2019	Moderate Risk of Bias
Olfson, M.; Gameroff, M. J.	2003	Moderate Risk of Bias
Oner, O.; Yilmaz, E. S.; Karadag, H.; Vural, M.; Vural, E. H.; Akbulat, A.; Gursoz, H.; Turkcapar, H.; Kerman, S.	2017	Low Risk of Bias

© 2021 Kazda L et al. *JAMA Network Open.*

Otasowie, J.; Castells, X.; Ehimare, U. P.; Smith, C. H.	2014	Low Risk of Bias
Owens, J.	2020	Low Risk of Bias
Owens, J.; Jackson, H.	2017	Low Risk of Bias
Parker, J.; Wales, G.; Chalhoub, N.; Harpin, V.	2013	Low Risk of Bias
Perez-Crespo, L.; J. Canals-Sans; E. Suades-Gonzalez; M. Guxens	2020	Low Risk of Bias
Perry, B. A.; Archer, K. R.; Song, Y.; Ma, Y.; Green, J. K.; Elefteriou, F.; Dahir, K. M.	2016	High Risk of Bias
Peyre, H.; Hoertel, N.; Cortese, S.; Acquaviva, E.; De Maricourt, P.; Limosin, F.; Delorme, R.	2014	Moderate Risk of Bias
Polanczyk, G. V.; Willcutt, E. G.; Salum, G. A.; Kieling, C.; Rohde, L. A.	2014	Moderate Risk of Bias
Polanczyk, G.; Caspi, A.; Houts, R.; Kollins, S. H.; Rohde, L. A.; Moffitt, T. E.	2010	Moderate Risk of Bias
Ponizovsky, A. M.; Marom, E.; Fitoussi, I.	2014	Low Risk of Bias
Pottegard, A.; Bjerregaard, B. K.; Glintborg, D.; Hallas, J.; Moreno, S. I.	2012	Low Risk of Bias
Poulton, A.	2005	High Risk of Bias
Poulton, A. S.; Bui, Q.; Melzer, E.; Evans, R.	2016	High Risk of Bias
Powell, S. G.; Frydenberg, M.; Thomsen, P. H.	2015	Moderate Risk of Bias
Pozzi, M.; Carnovale, C.; Peeters, Ggam; Gentili, M.; Antoniazzi, S.; Radice, S.; Clementi, E.; Nobile, M.	2018	Moderate Risk of Bias
Prasad, V.; Brogan, E.; Mulvaney, C.; Grainge, M.; Stanton, W.; Sayal, K.	2013	Moderate Risk of Bias
Prasad, V.; West, J.; Kendrick, D.; Sayal, K.	2018	Low Risk of Bias
Prosser, B.; Lambert, M. C.; Reid, R.	2015	Moderate Risk of Bias
Prosser, B.; Reid, R.	2009	Moderate Risk of Bias
Punja, S.; Shamseer, L.; Hartling, L.; Urichuk, L.; Vandermeer, B.; Nikles, J.; Vohra, S.	2016	Low Risk of Bias
Raman, S. R.; Marshall, S. W.; Haynes, K.; Gaynes, B. N.; Naftel, A. J.; Sturmer, T.	2013	Moderate Risk of Bias
Raman, Sudha R.; Man, Kenneth K.; Bahmanyar, Shahram; Berard, Anick; Bilder, Scott; Boukhris, Takoua; Bushnell, Greta; Crystal, Stephen; Furu, Kari; KaoYang, Yea-Huei; Karlstad, Oystein; Kieler, Helle; Kubota, Kiyoshi; Lai, Edward Chia-Cheng; Martikainen, Jaana E.; Maura, Geric; Moore, Nicholas; Montero, Dolores; Nakamura, Hidefumi; Neumann, Anke; Pate, Virginia; Pottegard, Anton; Pratt, Nicole L.; Roughead, Elizabeth E.; Macias Saint-Gerons, Diego; Sturmer, Til; Su, Chien-Chou; Zoega, Helga; Sturkenbroom, Miriam C.; Chan, Esther W.; Coghill, David; Ip, Patrick; Wong, Ian C.	2018	Low Risk of Bias
Renoux, C.; Shin, J. Y.; Dell'Aniello, S.; Fergusson, E.; Suissa, S.	2016	Moderate Risk of Bias
Reyes, N.; Baumgardner, D. J.; Simmons, D. H.; Buckingham, W.	2013	High Risk of Bias
Riera, M.; Castells, X.; Tobias, A.; Cunill, R.; Blanco, L.; Capella, D.	2017	Low Risk of Bias

© 2021 Kazda L et al. *JAMA Network Open.*

Robison, L. M.; Sclar, D. A.; Skaer, T. L.; Galin, R. S.	1999	Moderate Risk of Bias
Robison, L. M.; Skaer, T. L.; Sclar, D. A.; Galin, R. S.	2002	Moderate Risk of Bias
Rogalin, Mathilda Tassinari; Nencini, Alessio	2015	High Risk of Bias
Romano, E.; Baillargeon, R. H.; Wu, H. X.; Robaey, P.; Tremblay, R. E.	2002	High Risk of Bias
Rowland, A. S.; Umbach, D. M.; Catoe, K. E.; Stallone, L.; Long, S.; Rabiner, D.; Naftel, A. J.; Panke, D.; Faulk, R.; Sandler, D. P.	2001	Moderate Risk of Bias
Rowland, Andrew S.; Umbach, David M.; Stallone, Lil; Naftel, A.; Bohlig, E.; Sandler, Dale P.	2002	Moderate Risk of Bias
Ruiz-Goikoetxea, M.; Cortese, S.; Aznarez-Sanado, M.; Magallon, S.; Alvarez Zallo, N.; Luis, E. O.; de Castro-Manglano, P.; Soutullo, C.; Arrondo, G.	2018	Low Risk of Bias
Rydell, M.; Lundstrom, S.; Gillberg, C.; Lichtenstein, P.; Larsson, H.	2018	Low Risk of Bias
Safer, D. J.	2018	High Risk of Bias
Safer, D. J.	2011	High Risk of Bias
Safer, D. J.; Krager, J. M.	1994	High Risk of Bias
Safer, D. J.; Krager, J. M.	1983	High Risk of Bias
Safer, Daniel J.; Krager, John M.	1984	High Risk of Bias
Salmelainen, P.	2002	Low Risk of Bias
Santosh, P. J.; E. Taylor; J. Swanson; T. Wigal; S. Chuang; M. Davies; L. Greenhill; J. Newcorn; L. E. Arnold; P. Jensen; B. Vitiello; G. Elliott; S. Hinshaw; L. Hechtman; H. Abikoff; W. Pelham; B. Hoza; B. Molina; K. Wells; J. Epstein; M. Posner	2005	Moderate Risk of Bias
Sawyer, Michael G.; Reece, Christy E.; Sawyer, Alyssa C.; Johnson, Sarah E.; Lawrence, David	2018	Low Risk of Bias
Sayal, K.; Chudal, R.; Hinkka-Yli-Salomaki, S.; Joelsson, P.; Sourander, A.	2017	Low Risk of Bias
Sayal, K.; Owen, V.; White, K.; Merrell, C.; Tymms, P.; Taylor, E.	2010	Moderate Risk of Bias
Sayal, K.; Washbrook, E.; Propper, C.	2015	Moderate Risk of Bias
Schachar, R et al	2002	Low Risk of Bias
Schachter, H. M.; Pham, B.; King, J.; Langford, S.; Moher, D.	2001	Low Risk of Bias
Scheffler, R. M.; S. P. Hinshaw; S. Modrek; P. Levine	2007	High Risk of Bias
Scheffler, R. M.; T. T. Brown; B. D. Fulton; S. Hinshaw; P. Levine; S. Stone	2009	Moderate Risk of Bias
Schelleman, H.; Bilker, W. B.; Strom, B. L.; Kimmel, S. E.; Newcomb, C.; Guevara, J. P.; Daniel, G. W.; Cziraky, M. J.; Hennessy, S.	2011	High Risk of Bias
Schmiedeler, S.; Schneider, W.	2014	Moderate Risk of Bias
Schneider, H.; Eisenberg, D.	2006	Low Risk of Bias
Schubert, I.; Koster, I.; Lehmkuhl, G.	2010	Low Risk of Bias
Schwandt, H.; A. Wuppermann	2016	Low Risk of Bias
Schwartz, S.; Correll, C. U.	2014	Moderate Risk of Bias

© 2021 Kazda L et al. *JAMA Network Open.*

Schweren, L.; Hoekstra, P.; van Lieshout, M.; Oosterlaan, J.; Lambregts-Rommelse, N.; Buitelaar, J.; Franke, B.; Hartman, C.	2019	Low Risk of Bias
Sclar, D. A.; Robison, L. M.; Bowen, K. A.; Schmidt, J. M.; Castillo, L. V.; Oganov, A. M.	2012	Moderate Risk of Bias
Sellers, R.; Maughan, B.; Pickles, A.; Thapar, A.; Collishaw, S.	2015	Moderate Risk of Bias
Setlik, Jennifer; Bond, G.; Ho, Mona	2009	Moderate Risk of Bias
Shem-Tov, S.; G. Chodick; D. Weitzman; G. Koren	2019	Low Risk of Bias
Shin, J. Y.; Roughead, E. E.; Park, B. J.; Pratt, N. L.	2016	Low Risk of Bias
Sibley, M. H.; Kuriyan, A. B.; Evans, S. W.; Waxmonsky, J. G.; Smith, B. H.	2014	High Risk of Bias
Sibley, Margaret H.; Rohde, Luis A.; Swanson, James M.; Hechtman, Lily T.; Molina, Brooke S.; Mitchell, John T.; Arnold, L.; Caye, Arthur; Kennedy, Traci M.; Roy, Arunima; Stehli, Annamarie	2018	Low Risk of Bias
Siegel, C. E.; Laska, E. M.; Wanderling, J. A.; Hernandez, J. C.; Levenson, R. B.	2016	Low Risk of Bias
Singh, I.	2011	High Risk of Bias
Skounti, M.; Philalithis, A.; Galanakis, E.	2007	High Risk of Bias
Smith, G.; B. Jongeling; P. Hartmann; C. Russell; L. Landau	2010	Moderate Risk of Bias
Song, I.; Lee, M. S.; Lee, E. K.; Shin, J. Y.	2018	Low Risk of Bias
Song, I.; Shin, J. Y.	2016	Low Risk of Bias
Song, M.; Dieckmann, N. F.; Nigg, J. T.	2018	Moderate Risk of Bias
Stinnett, Terry A.; Crawford, Stephanie A.; Gillespie, Marci D.; Cruce, Michael K.; Langford, Courtney A.	2001	High Risk of Bias
Storebo, O. J.; Pedersen, N.; Ramstad, E.; Kielsholm, M. L.; Nielsen, S. S.; Krogh, H. B.; Moreira-Maia, C. R.; Magnusson, F. L.; Holmskov, M.; Gerner, T.; Skoog, M.; Rosendal, S.; Groth, C.; Gillies, D.; Buch Rasmussen, K.; Gauci, D.; Zwi, M.; Kirubakaran, R.; Hakonsen, S. J.; Aagaard, L.; Simonsen, E.; Gluud, C.	2018	Low Risk of Bias
Storebo, O. J.; Ramstad, E.; Krogh, H. B.; Nilausen, T. D.; Skoog, M.; Holmskov, M.; Rosendal, S.; Groth, C.; Magnusson, F. L.; Moreira-Maia, C. R.; Gillies, D.; Buch Rasmussen, K.; Gauci, D.; Zwi, M.; Kirubakaran, R.; Forsbol, B.; Simonsen, E.; Gluud, C.	2015	Low Risk of Bias
Stuckelman, Z. D.; Mulqueen, J. M.; Ferracioli-Oda, E.; Cohen, S. C.; Coughlin, C. G.; Leckman, J. F.; Bloch, M. H.	2017	Moderate Risk of Bias
Stuhec, M.; Locatelli, I.; Svab, V.	2015	Moderate Risk of Bias
Stuhec, M.; Svab, V.; Locatelli, I.	2015	Moderate Risk of Bias

© 2021 Kazda L et al. *JAMA Network Open.*

Swanson, James M.; Arnold, L.; Molina, Brooke S.; Sibley, Margaret H.; Hechtman, Lily T.; Hinshaw, Stephen P.; Abikoff, Howard B.; Stehli, Annamarie; Owens, Elizabeth B.; Mitchell, John T.; Nichols, Quyen; Howard, Andrea; Greenhill, Laurence L.; Hoza, Betsy; Newcorn, Jeffrey H.; Jensen, Peter S.; Vitiello, Benedetto; Wigal, Timothy; Epstein, Jeffery N.; Tamm, Leanne; Lakes, Kimberly D.; Waxmonsky, James; Lerner, Marc; Etcovitch, Joy; Murray, Desiree W.; Muenke, Maximilian; Acosta, Maria T.; Arcos-Burgos, Mauricio; Pelham, William E.; Kraemer, Helena C.	2017	Low Risk of Bias
Thomas, C. P.; Conrad, P.; Casler, R.; Goodman, E.	2006	Moderate Risk of Bias
Thomas, R.; Sanders, S.; Doust, J.; Beller, E.; Glasziou, P.	2015	Low Risk of Bias
Toh, S.	2006	Moderate Risk of Bias
Treceno, C.; Martin Arias, L. H.; Sainz, M.; Salado, I.; Garcia Ortega, P.; Velasco, V.; Jimeno, N.; Escudero, A.; Velasco, A.; Carvajal, A.	2012	Moderate Risk of Bias
Trip, A. M.; Visser, S. T.; Kalverdijk, L. J.; De Jong-Van Den Berg, L. T. W.	2009	Low Risk of Bias
Valdizan-Uson, J.; Canovas-Martinez, A.; De Lucas-Taracena, M.; Diaz-Atienza, F.; Eddy-Ives, L.; Fernandez-Jaen, A.; Fernandez-Perez, M.; Garcia-Giral, M.; Garcia-Magan, P.; Garraus-Oneca, M.; Idiazabal-Alecha, M.; Lopez-Benito, M.; Lorenzo-Sanz, G.; Martinez-Anton, J.; Martinez-Granero, M.; Montanes-Rada, F.; Mulas-Delgado, F.; Ochando-Perales, G.; Ortega-Garcia, E.; Pelaz-Antolin, A.; Ramos-Quiroga, J.; Ruiz-Sanz, F.; Vaquerizo-Madrid, J.; Yusta-Izquierdo, A.	2013	High Risk of Bias
Valentine, J.; Zubrick, S.; Sly, P.	1996	High Risk of Bias
Van Den Ban, E.; Souverein, P.; Meijer, W.; Van Engeland, H.; Swaab, H.; Egberts, T.; Heerdink, E.	2014	Low Risk of Bias
Van Den Ban, E.; Souverein, P.; Swaab, H.; Van Engeland, H.; Heerdink, R.; Egberts, T.	2010	Low Risk of Bias
Van der Oord, S.; Prins, P. J.; Oosterlaan, J.; Emmelkamp, P. M.	2008	High Risk of Bias
van Lieshout, M.; Luman, M.; Twisk, J. W.; van Ewijk, H.; Groenman, A. P.; Thissen, A. J.; Faraone, S. V.; Heslenfeld, D. J.; Hartman, C. A.; Hoekstra, P. J.; Franke, B.; Buitelaar, J. K.; Rommelse, N. N.; Oosterlaan, J.	2016	Moderate Risk of Bias
Vande Voort, J. L.; He, J. P.; Jameson, N. D.; Merikangas, K. R.	2014	Moderate Risk of Bias
Vasiliadis, H. M.; Diallo, F. B.; Rochette, L.; Smith, M.; Langille, D.; Lin, E.; Kisely, S.; Fombonne, E.; Thompson, A. H.; Renaud, J.; Lesage, A.	2017	Low Risk of Bias
Vinker, S.; Vinker, R.; Elhayany, A.	2006	Low Risk of Bias
Visser, Susanna N.; Danielson, Melissa L.; Bitsko, Rebecca H.; Holbrook, Joseph R.; Kogan, Michael D.; Ghandour, Reem M.; Perou, Ruth; Blumberg, Stephen J.	2014	Moderate Risk of Bias

© 2021 Kazda L et al. *JAMA Network Open.*

Vitiello, Benedetto; Elliott, Glen R.; Swanson, James M.; Arnold, L.; Hechtman, Lily; Abikoff, Howard; Molina, Brooke S.; Wells, Karen; Wigal, Timothy; Jensen, Peter S.; Greenhill, Laurence L.; Kaltman, Jonathan R.; Severe, Joanne B.; Odbert, Carol; Hur, Kwan; Gibbons, Robert	2012	Low Risk of Bias
Vuori, M; Kski-Pirila, A;Martikainen, J E; Saastamoinen, L	2020	Moderate Risk of Bias
Wang, L. J.; Chen, C. K.; Huang, Y. S.	2015	High Risk of Bias
Wang, L. J.; Lee, S. Y.; Yuan, S. S.; Yang, C. J.; Yang, K. C.; Lee, T. L.; Shyu, Y. C.	2016	Low Risk of Bias
Wang, L.; Lee, S.; Yuan, S.; Yang, C.; Yang, K.; Huang, T.; Chou, W.; Chou, M.; Lee, M.; Lee, T.; Shyu, Y.	2017	Low Risk of Bias
Westover, A. N.; Halm, E. A.	2012	High Risk of Bias
Whalen, C. K.; Jamner, L. D.; Henker, B.; Delfino, R. J.; Lozano, J. M.	2002	High Risk of Bias
Whitely, M.; Raven, M.; Timimi, S.; Jureidini, J.; Phillimore, J.; Leo, J.; Moncrieff, J.; Landman, P.	2019	Moderate Risk of Bias
Wienen, A. W.; Sluiter, M. N.; Thoutenhoofd, E.; de Jonge, P.; Batstra, L.	2019	High Risk of Bias
Wilens, T. E.; Adamson, J.; Monuteaux, M. C.; Faraone, S. V.; Schillinger, M.; Westerberg, D.; Biederman, J.	2008	High Risk of Bias
Wilens, T. E.; Faraone, S. V.; Biederman, J.; Gunawardene, S.	2003	High Risk of Bias
Wilens, T.E.; L. A. Adler; J. Adams; S. Sgambati; J. Rotrosen; R. Sawtelle; L. Utzinger; S. Fusillo	2008	High Risk of Bias
Winterstein, A. G.; Gerhard, T.; Kubilis, P.; Saidi, A.; Linden, S.; Crystal, S.; Zito, J.; Shuster, J. J.; Olfson, M.	2012	Low Risk of Bias
Winterstein, A. G.; Gerhard, T.; Shuster, J.; Zito, J.; Johnson, M.; Liu, H.; Saidi, A.	2008	Low Risk of Bias
Winterstein, A.G.; Y. Li; T. Gerhard; S. Linden; J. J. Shuster	2020	Low Risk of Bias
Wolraich, M. L.; Hannah, J. N.; Baumgaertel, A.; Feurer, I. D.	1998	Moderate Risk of Bias
Wolraich, M. L.; Hannah, J. N.; Pinnock, T. Y.; Baumgaertel, A.; Brown, J.	1996	High Risk of Bias
Xu, G.; Strathearn, L.; Liu, B.; Yang, B.; Bao, W.	2018	Moderate Risk of Bias
Yoon, E. Y.; Cohn, L.; Rocchini, A.; Kershaw, D.; Clark, S. J.	2012	Moderate Risk of Bias
Yoshida, M.; Obara, T.; Kikuchi, S.; Satoh, M.; Morikawa, Y.; Ooba, N.; Yamaguchi, H.; Mano, N.	2019	Low Risk of Bias
Zablotsky, B.; L. I. Black; M. J. Maenner; L. A. Schieve; M. L. Danielson; R. H. Bitsko; S. J. Blumberg; M. D. Kogan; C. A. Boyle	2019	Moderate Risk of Bias
Zachor, D. A.; Roberts, A. W.; Bart Hodgens, J.; Isaacs, J. S.; Merrick, J.	2006	High Risk of Bias
Zetterqvist, J.; Asherson, P.; Halldner, L.; Langstrom, N.; Larsson, H.	2013	Low Risk of Bias
Zhang, H et al	2010	High Risk of Bias
Zito, J. M.; Safer, D. J.; dosReis, S.; Gardner, J. F.; Boles, M.; Lynch, F.	2000	Low Risk of Bias

© 2021 Kazda L et al. *JAMA Network Open.*

Zito, J. M.; Safer, D. J.; DosReis, S.; Magder, L. S.; Gardner, J. F.; Zarin, D. A.	1999	Moderate Risk of Bias
Zoega, H.; Rothman, K. J.; Huybrechts, K. F.; Olafsson, O.; Baldursson, G.; Almarsdottir, A. B.; Jonsdottir, S.; Halldorsson, M.; Hernandez-Diaz, S.; Valdimarsdottir, U. A.	2012	Low Risk of Bias
Zuvekas, S. H.; Vitiello, B.	2012	Moderate Risk of Bias
Zuvekas, S. H.; Vitiello, B.; Norquist, G. S.	2006	Moderate Risk of Bias

All details for each critical appraisal can be found here.

© 2021 Kazda L et al. *JAMA Network Open.*

eAppendix 6. Results Table

**Type of evidence (n)**	**Theme (n)**	**Subtheme (n)**	**Main outcomes (n)**
**Q1 Is there potential for increased diagnosis (n=104)**			
Prevalence Variations (68)	in Subpopulations (48) by	Gender (25)	• Lower diagnosis in girls than boys (12)[2-13]<br>• with no change in ratios over time (1)[14]<br>• with decreasing ratios over time (8)[15-22]<br>• and lower symptomatic prevalence in girls (2)[23,24]<br>• Symptomatic girls less likely to be diagnosed (2)[25,26]
		SES/ Insurance status (21)	• Higher diagnosis in lower SES (12)[3-5,7,8,10,13,21,22,27-29]<br>• and higher symptomatic prevalence (1)[24]<br>• Higher diagnosis in higher SES (1)[30]<br>• Higher diagnosis in public vs private health insurance (2)[13,29]<br>• but ratios decreasing over time (3)[18,21,31]<br>• Lower diagnosis in no vs any health insurance (7)[13,21,25,29,32-34]<br>• Lower diagnosis in lower SES in rural areas but not in urban areas (1)[35]<br>• No effect of insurance status on diagnosis in hypothetical scenario (1)[26]
		Race/ Ethnicity/ Indigenous status (21)	• Lower diagnosis in black/ Hispanic vs white youths (7)[3,4,6,9,10,13,33]<br>• but decreasing ratios over time (sometimes black rates overtaking white ones) (5)[16,18,22,27,36]<br>• but not lower symptomatic prevalence (2)[32,37]<br>• Lower diagnosis in whites than in black youths (4)[5,11,12,38]<br>• Lower diagnosis in youths from non-English speaking background (2)[3,4]<br>• No effect of race on diagnosis in hypothetical scenario (1)[26]<br>• Youths with migrant background less likely to be diagnosed (1)[24]
		Relative age (12)	• Youngest children in class much more likely to be diagnosed than oldest (11)[39-49]<br>• In a setting with low diagnosis where only specialists can diagnose, there is no difference in diagnosis probability between youngest and oldest in class (1)[50]

©2021 Kazda L et al. *JAMA Network Open*

Type of evidence (n)	Theme (n)	Subtheme (n)	Main outcomes (n)
		Location/ Region (8)	• Large variations in diagnosis by region (8)[2-5,8,22,27,35]
		Other (1)	• Higher diagnosis and higher teacher perceived symptomatic prevalence in larger classrooms (1)[6]
	by Diagnosis (20)	Diagnostic criteria (20)	• Broadening of criteria leads to increases in potential cases (18) for comparisons between:<br>◦ DSMIII and DSMIIIR (3)[51-53]<br>◦ DSMIIIR and DSMIV (2)[12,54]<br>◦ DSMIV to DSM5 (2)[55,56]<br>◦ DSMIII, DSMIIIR and DSMIV (4)[57-60]<br>◦ DSMIII, DSMIIIR, DSMIV and DSM5 (1)[61]<br>◦ DSMIII, DSMIIIR and ICD10 (1)[62]<br>◦ DSMIV and ICD10 (3)[63-65]<br>◦ DSMIV with early onset or late onset (2)[66,67]<br>• Broadening of criteria leads to small/ minimal increases in potential cases (2) for comparisons between:<br>◦ DSMIV with early onset or late onset (1)[68]<br>◦ DSM5 with early onset or late onset (1)[69]
Reservoir (44) due to	Medicalisation (3) of	Behavioural problems (3)	• Society's decreasing tolerance towards different behaviour leads to increased range of behaviour identified and diagnosed as abnormal (2)[28,70]<br>• Mental health professionals from China/ Indonesia rating same attention difficulties higher than those from USA/ Japan (1)[71]
	Phenotype changes (5)	Trends over time (5)	• Percentage of youths with clinically significant symptoms declined/stable over time (3)[72-74]<br>• Percentage of youths with clinically significant symptoms increased within one year (1)[75]<br>• Mean hyperactivity scores are stable over time (1)[76]<br>• Percentage of youths with subthreshold symptoms increased over time (1)[72]
	Diagnostic inaccuracy (16) leading to	Over-/Underdiagnosis (16)	• Potential over- and underdiagnosis occurring due to false-negatives and -positives (6)[34,37,77-80]

©2021 Kazda L et al. *JAMA Network Open*

Type of evidence (n)	Theme (n)	Subtheme (n)	Main outcomes (n)
			• Potential underdiagnosis due to false-negatives (5)[23,72,73,81,82]<br>• Potential overdiagnosis due to false-positives (5)[25,54,75,83,84]
	Spectrum of disorder (22) indicated by	Impact of extend of symptoms over time (13)	• Continuous dose-response relationships between increasing symptoms and increased risk of various later adverse outcomes (educational, employment, substance use, social and emotional difficulties), even at subthreshold level (13)[85-97]
		Subthreshold prevalence (8)	• Large reservoirs (very varied estimated depending on criteria used) of youths displaying subthreshold symptoms (6)[85-87,91,92,98]<br>• Percentage of youths displaying full- or subthreshold symptoms declines significantly with age until early adulthood (2)[99,100]
		Verification of dimensional structure (7)	• Inattention, hyperactivity and impulsivity problems are best viewed on a continuum with ADHD as the extreme end of a spectrum (7)[96,101-106]
**Q2 Is diagnosis actually increased? (n=45)**			
Diagnosis (45)	Trend over time (45) indicated by	Change in prevalence (30)	• Increasing trend (27)[5,14,15,18-21,31,36,46,73,107-122]<br>• Increasing trend until early 2000s (3)[2,30,123]<br>(data from 1989-2016)
		Change in incidence (12)	• Increasing trend (11)[7,17,19,110,118,120,124-128]<br>• Increasing trend until 2007 (1)[30]<br>(data from 1995-2017)
		Change in lifetime prevalence (12)	• Increasing trend (11)[4,13,16,17,21,22,27,72,73,128,129]<br>• Stagnant in adolescent, increasing in children (1)[29]<br>(data from 1996-2015)
**Q3 Are additional cases milder? (n=25)**			
	Impairment levels (11) indicated by	Change in adverse outcomes over time (2)	• Behaviour and academic problems decrease with increasing group of youths with ADHD (1)[75]

©2021 Kazda L et al. *JAMA Network Open*

Type of evidence (n)	Theme (n)	Subtheme (n)	Main outcomes (n)
Severity of disorder (17) indicated by			• Reduction in hospital visits in medicated children is decreased with increasing group of youths with ADHD (1)*[129]
		Difference in adverse outcomes by diagnostic criteria (9)	• Youths fulfilling ADHD criteria are less likely to be impaired in various domains with broadening of diagnostic criteria and increasing of group (5)[53,54,57,62,65], only marginal effect in this direction (1)[64]<br>• Reduction in ADHD symptoms and social impairments in medicated children is decreased with broadening of ADHD criteria (1)*[130]<br>• Sample size too small to detect differences (2)[52,68]
	Symptom severity (6) indicated by	Trend over time (3)	• Severity proportions stable (1)[109]<br>• Larger increase in moderate/ severe cases (2)[21,116]
		Proportions of mild, moderate, severe cases (2)	• Proportion of youths with severe ADHD is (very) low (2)[4,131]
		Change by diagnostic criteria (1)	• Additional late onset cases do not differ from early onset ones in symptom severity (1)[67]
Level of symptoms (8)	Clinically significant symptom prevalence (8) by	Change over time (compared to change in official diagnoses) (6)	• Prevalence estimates of symptomatic ADHD have remained steady or slightly declined (4)[59,60,74,76]<br>• while diagnostic prevalence has increased (2)[72,73]
		Change in relative age effect over time (2)	• The relative age effect (younger children are more likely to be diagnosed than older children in the same year of school) has increased in later birth cohorts (2)[41,47]
**Q4 Are additional cases treated? (n=83)**			
Medication (83)	Trend over time (83) indicated by	Change in prevalent treatment rate (58) (in youths)	• Increasing trend (55)[5,14,15,17,21,29,46,111,114,119-121,128,129,132-172]<br>• Decreases in <6-year-olds, increases in >6-year-olds (2)[173,174]<br>• Mostly decreasing trend (2)[112,142]<br>(data from 1971-2018)

©2021 Kazda L et al. *JAMA Network Open*

Type of evidence (n)	Theme (n)	Subtheme (n)	Main outcomes (n)
		Change in incident treatment rate (9)<br>(in youths)	• Increasing trend (5)[120,145,154,175,176]<br>• Stable/ variable rate (4)[151,177-179]<br>(data from 1977-2008)
		Change in medication usage (11)<br>(in scripts/doses/sales)	• Increasing trend (10)[14,180-188]<br>• Increasing trend until 2010 (1)[189]<br>(data from 1992-2012)
		Change in consultations in which ADHD medication is prescribed (10)	• Increasing trend (9)[18,20,109,113,117,122,123,190,191]<br>• Increasing trend until 2004 in <6-year-olds (1)[192]<br>(data from 1989-2015)
**Q5a Might harms outweigh benefits of diagnosis? (n=31)**			
Potential benefits of the diagnosis (15)	Empowerment (14) through	Explanation for problems (11)	• Increased legitimacy and validation (5)[28,193-196]<br>• Increased understanding, sympathy and reassurance (8)[194-201]<br>• Reduced feelings of guilt, blame, fault, failure and anger (6)[28,193,194,198,200,202]
		Increased control (6)	• Reduced uncertainty leads to reclaiming of identity/ feeling of belonging (3)[194,197,202]<br>• Increased self-esteem and confidence (3)[194,197,199]<br>• Expectation of solution (4)[193,194,197,200]
	Enablement (10) to	Support (10)	• Increased ability to seek, receive and accept support (4)[194,195,200,202]:<br>• educational (3)[193,196,200]<br>• financial (1)[28]<br>• medical/psychological (4)[70,193,198,199]
Potential harms of the diagnosis (29)	Disempowerment (22) through	Excuse for problems (15)	• Decreased responsibility for behaviour, parenting and teaching problems (6)[70,193,195,199,203,204]<br>• Increased deflection from underlying social, systemic or individual problems (3)[28,193,194]<br>• No meaningful benefit from diagnosis/ no change apart from label (10)[70,80,195,197,199,200,205-208]

©2021 Kazda L et al. *JAMA Network Open*

Type of evidence (n)	Theme (n)	Subtheme (n)	Main outcomes (n)
		Loss of control (15)	• Diagnosis as first step to behaviour correction, control and manipulation by others (4)[70,193,199,202]<br>• Increased passiveness and hopelessness (3)[194,195,200]<br>• Self-fulfilling prophecy: perceived inability to change or achieve (by self or others) leads to exclusion and reduced opportunities (9)*[34,194,196,209-214]
	Stigmatisation (14) through	Permanent label/ identity (14)	• Creates an identity which enhances prejudice, stereotypes, judgement (14)[194-197,199,200,203,209,213,215-219]<br>• Increases feelings of isolation, exclusion and shame (3)[195,199,203]
**Q5b Might harms outweigh benefits of treatment (n=120)**			
Outcomes of pharmacological treatment (120)	Academic (19)	Cognitive functioning (3)	• Only improvements in commission errors, all other unchanged (1)[220]<br>• Positive impact on several aspects of cognition (1)[221]<br>• No effect after wash-out period (1)[222]
		Motor functioning (1)	• Significant improvements in motor skills (1)[223]
		Academic performance (15)	• Treated children have worse educational outcomes compared to the rest of the population (1)[224]<br>• No effect of treatment compared to untreated/ less treated youths with ADHD (3)[225-227]<br>• Small positive effect of treatment in some areas compared to untreated youths with ADHD (6)[228-233]<br>• Substantial improvement with treatment compared to untreated/ less treated youths (2)[234,235]<br>• Population level deterioration in academic outcomes per unit increase in ADHD score after increased uptake of medication treatment (1)[236]<br>• Potential harmful effect, especially in youths with less severe symptoms (1)*[34]<br>• Medication treatment only beneficial for youths with more severe symptoms (1)*[211]
	Accidents (12)	ED use/ Hospital admissions (5)	• Fewer hospital contacts in treated vs untreated youths but effects are smaller in later, larger diagnosed and treated cohorts (1)*[129]

©2021 Kazda L et al. *JAMA Network Open*

Type of evidence (n)	Theme (n)	Subtheme (n)	Main outcomes (n)
			• No effect on hospital contacts during treated vs before treatment periods (1)[237]<br>• No difference in hospital contacts between ever treated vs never treated youths with ADHD (1)[238]<br>• But fewer contacts during treated vs untreated periods (2)[238,239]<br>• Treated children have worse health outcomes compared to the rest of the population (1)[224]
		Injuries (7)	• Lower risk of injuries during treated vs untreated periods (3)[239-241] and in treated vs untreated youths (2)[242,243]<br>• No difference in injuries (1)[244] or motor vehicle accidents (1)[245] during treated vs untreated periods
		Poisoning (1)	• Significant increase in hospital admissions for unintentional poisoning with ADHD medication (1)[246]
	Cardiovascular (8)	Blood pressure/ Heart rate (2)	• No effect of treatment on blood pressure (2)[247,248]<br>• Significant long-term effect of stimulant treatment on heart rate (1)[247]
		Safety (6)	• No effect of treatment on severe cardiovascular events (3)[249-251]<br>• Increased risk of arrythmia (1)[252] or of any cardiac evet requiring hospitalisation (1)[253] with treatment<br>• Potential for increased risk but not enough statistical power to detect small differences (3)[249,250,254]
	Efficacy (30)	Symptom reduction (30)	• Significant short-term (<12 months) symptom reduction in many youths with treatment (24)[175,220,227,255-275]<br>• No evidence of any symptom improvement after 48-hour wash-out period (1)[222]<br>• No difference in symptoms between regularly treated and untreated/irregularly treated ADHD youths in late adolescence/ early adulthood (3)[225,276,277]<br>• Subjects with more severe symptoms at baseline showed greatest treatment response in the long-term (5 years) (1)*[278]

©2021 Kazda L et al. *JAMA Network Open*

Type of evidence (n)	Theme (n)	Subtheme (n)	Main outcomes (n)
	Physical (14)	Activity (2)	• Reduced levels of physical activity in medicated youths compared to unmedicated (1)[279] and in treated vs untreated periods (1)[280]
		Height (12)	• Growth delay observed with treatment (2)[281,282]<br>• Decreased growth with treatment (6)[276,283-287]<br>• No effect observed/ significant heterogeneity (4)[248,279,288,289]
		Weight (5)	• No effect observed (2)[279,282]<br>• Reduction in weight in medicated youths (3)[283,284,287]
	Psychological (20)	Other (2)	• Increased risk of psychosis with amphetamine treatment (1)[290]<br>• Significant increase in tics with current treatment vs never or previously treated (1)[291]
		Substance abuse (12)	• No effect of treatment on later substance abuse (4)[292-295]<br>• Decreased risk of subsequent substance abuse in ever treated vs never treated youths (5)[296-300]<br>• Increased risk of stimulant abuse in ever treated subjects vs never treated (1)[301]<br>• Significant prescription stimulant misuse and diversion prevalence amongst youths with and without ADHD (2)[302,303]
		Suicidal behaviour (6)	• No effect of treatment on risk of suicidal behaviour (2)[304,305]<br>• Reduced risk of suicidal behaviour during treatment periods compared to untreated period (2)[306,307] and in treated vs untreated youths (1)[308]<br>• Increased risk of suicidal behaviour with treatment (1)[309]
	Social/ Emotional (11)	Emotional (3)	• Increased risk of irritability with amphetamine treatment (1)[310]<br>• Mixed outcomes for various emotional behaviours with stimulant treatment (some increased, some decreased) (1)[311]<br>• Population level deterioration in happiness per unit increase in ADHD score after increased uptake of medication treatment (1)[236]
		Criminal behaviour (3)	• Reduced risk of conviction and incarceration during treatment periods compared to periods without treatment (1)[312]

©2021 Kazda L et al. *JAMA Network Open*

Type of evidence (n)	Theme (n)	Subtheme (n)	Main outcomes (n)
			• No difference in risk of receiving driving citation for treated vs untreated periods (1)[245]<br>• Reduced risk of being charged with a crime in treated vs untreated youths but effects are smaller in later, larger diagnosed and treated cohorts (1)*[129]
		Social impairment (2)	• No improvements in social impairment for most domains with treatment vs before treatment (1)[226]<br>• Potentially clinically relevant improvements in some domains with treatment vs placebo (1)[313]
		Quality of life (4)	• Small short-term improvements in quality of life with treatment (3)[313-315]<br>• No effect detected (1)[316]
	Tolerability (29)	Adverse events (25)	• Low occurrence of mild AEs (2)[175,257]<br>• Relatively common mild/moderate AEs (16)[256,258,260,261,266,269,271,272,274,275,278,317-321]<br>• Young children more vulnerable to AEs (2)[275,322]<br>• Reporting of AEs is unsatisfactory (6)[262,271,274,323-325]<br>• Serious AEs seem rare but difficult to determine from reported data (5)[267,317,319,324,325]<br>• Common AEs: Appetite suppression, insomnia, headaches, weight loss, abdominal pain, emotional symptoms, anxiousness, sleep disturbance, fatigue, dizziness, nausea
		Discontinuation (6)	• Moderate to high discontinuation rates (20-44%) (4)[270,320,326,327]<br>• Discontinuation similar to placebo group/ low quality evidence (2)[255,265]
	Various (8)	Mixed (8)	• 50% of various outcomes reported some beneficial treatment effect (1)[328]<br>• Reduced risk of various outcomes (e.g. injuries, MVAs, academic difficulties, criminality, substance abuse, depression, seizures) (2)[329,330]<br>• Lack of methodologically sound research upon which to base decisions (1)[331]<br>• Longer treatment duration indicated better outcomes for various functionality and problem domains (1)[332]<br>• No long-term treatment effect for various functioning outcomes (e.g. academic achievement, social/ emotional skills, psychological problems) (3)[333-335]

©2021 Kazda L et al. *JAMA Network Open*

eReferences

1. Moher D, Liberati A, Tetzlaff J, Altman D, The PRSIMA Group. Preferred Reporting Items for Systematic Reviews and MetaAnalyses: The PRISMA Statement. *PLoS Med.* 2009;6(7).
2. Akmatov MK, Steffen A, Holstiege J, Hering R, Schulz M, Batzing J. Trends and regional variations in the administrative prevalence of attention-deficit/hyperactivity disorder among children and adolescents in Germany. *Scientific Reports.* 2018;8(1):17029.
3. Centers for Disease C, Prevention. Mental health in the United States. Prevalence of diagnosis and medication treatment for attention-deficit/hyperactivity disorder--United States, 2003. *Morbidity & Mortality Weekly Report.* 2005;54(34):842-847.
4. Centers for Disease C, Prevention. Increasing prevalence of parent-reported attention-deficit/hyperactivity disorder among children --- United States, 2003 and 2007. *Morbidity & Mortality Weekly Report.* 2010;59(44):1439-1443.
5. Danielson ML, Visser SN, Gleason MM, Peacock G, Claussen AH, Blumberg SJ. A National Profile of Attention-Deficit Hyperactivity Disorder Diagnosis and Treatment Among US Children Aged 2 to 5 Years. *JJournal of Developmental and Behavioral Pediatrics.* 2017;38(7):455-464.
6. Havey J, Olson JM, McCormick C, Cates GL. Teachers' Perceptions of the Incidence and Management of Attention-Deficit Hyperactivity Disorder. *Applied Neuropsychology.* 2005;12(2):120-127.
7. Hire AJ, Ashcroft DM, Springate DA, Steinke DT. ADHD in the United Kingdom: Regional and Socioeconomic Variations in Incidence Rates Amongst Children and Adolescents (2004-2013). *Journal of Attention Disorders.* 2018;22(2):134-142.
8. Prasad V, West J, Kendrick D, Sayal K. Attention-deficit/hyperactivity disorder: Variation by socioeconomic deprivation. *Archives of Disease in Childhood.* 2018;30.
9. Rowland AS, Umbach DM, Stallone L, Naftel A, Bohlig E, Sandler DP. Prevalence of medication treatment for attention deficit--hyperactivity disorder among elementary school children in Johnston County, North Carolina. *American Journal of Public Health.* 2002;92(2):231-234.
10. Schneider H, Eisenberg D. Who receives a diagnosis of attention-deficit/hyperactivity disorder in the United States elementary school population? *Pediatrics.* 2006;117(4):e601-e609.
11. Siegel CE, Laska EM, Wanderling JA, Hernandez JC, Levenson RB. Prevalence and diagnosis rates of childhood ADHD among racial-ethnic groups in a public mental health system. *Psychiatric Services.* 2016;67(2):199-205.
12. Skounti M, Philalithis A, Galanakis E. Variations in prevalence of attention deficit hyperactivity disorder worldwide. *European Journal of Pediatrics.* 2007;166(2):117-123.
13. Zablotsky B, Black LI, Maenner MJ, et al. Prevalence and Trends of Developmental Disabilities among Children in the United States: 2009-2017. *Pediatrics.* 2019;144(4):10.
14. Schubert I, Koster I, Lehmkuhl G. The changing prevalence of attention-deficit/hyperactivity disorder and methylphenidate prescriptions: A study of data from a random sample of insurees of the AOK Health Insurance Company in the German state of Hesse, 2000-2007. [German, English]. *Deutsches Arzteblatt.* 2010;107(36):615-621.
15. Brault MC, Lacourse E. Prevalence of prescribed attention-deficit hyperactivity disorder medications and diagnosis among canadian preschoolers and school-age children: 1994-2007. *Canadian Journal of Psychiatry.* 2012;57(2):93-101.
16. Collins KP, Cleary SD. Racial and ethnic disparities in parent-reported diagnosis of ADHD: national survey of children's health (2003, 2007, and 2011). *Journal of Clinical Psychiatry.* 2016;77(1):52-59.
17. Davidovitch M, Koren G, Fund N, Shrem M, Porath A. Challenges in defining the rates of ADHD diagnosis and treatment: Trends over the last decade. *BMC Pediatrics.* 2017;17 (1) (no pagination)(218).
18. Fairman KA, Peckham AM, Sclar DA. Diagnosis and Treatment of ADHD in the United States: Update by Gender and Race. *Journal of Attention Disorders.* 2020;24(1):10-19.
19. Huang CL, Chu CC, Cheng TJ, Weng SF. Epidemiology of treated attention-deficit/hyperactivity disorder (ADHD) across the lifespan in Taiwan: a nationwide population-based longitudinal study. *PLoS ONE.* 2014;9(4):e95014.

©2021 Kazda L et al. *JAMA Network Open*

20. Robison LM, Skaer TL, Sclar DA, Galin RS. Is attention deficit hyperactivity disorder increasing among girls in the US? Trends in diagnosis and the prescribing of stimulants. *CNS Drugs.* 2002;16(2):129-137.
21. Visser SN, Danielson ML, Bitsko RH, et al. Trends in the parent-report of health care provider-diagnosed and medicated attention-deficit/hyperactivity disorder: United states, 2003-2011. *J Am Acad Child Psy.* 2014;53(1):34-46.
22. Xu G, Strathearn L, Liu B, Yang B, Bao W. Twenty-Year Trends in Diagnosed Attention-Deficit/Hyperactivity Disorder Among US Children and Adolescents, 1997-2016. *JAMA netw.* 2018;1(4):e181471.
23. Cuffe SP, Moore CG, McKeown RE. Prevalence and correlates of ADHD symptoms in the National Health Interview Survey. *Journal of Attention Disorders.* 2005;9(2):392-401.
24. Huss M, Holling H, Kurth BM, Schlack R. How often are german children and adolescents diagnosed with ADHD? Prevalence based on the judgment of health care professionals: Results of the german health and examination survey (KiGGS). *European Child and Adolescent Psychiatry.* 2008;17(SUPPL. 1):52-58.
25. Froehlich TE, Lanphear BP, Epstein JN, Barbaresi WJ, Katusic SK, Kahn RS. Prevalence, recognition, and treatment of attention-deficit/hyperactivity disorder in a national sample of US children. *Archives of Pediatrics and Adolescent Medicine.* 2007;161(9):857-864.
26. Morley CP. The effects of patient characteristics on ADHD diagnosis and treatment: a factorial study of family physicians. *BMC Family Practice.* 2010;11:11.
27. Akinbami LJ, Liu X, Pastor PN, Reuben CA. Attention deficit hyperactivity disorder among children aged 5-17 years in the United States, 1998-2009. *NCHS data brief.* 2011(70):1-8.
28. Allan J, Harwood V. Medicus interruptus in the behaviour of children in disadvantaged contexts in Scotland. *Brit J Sociol Educ.* 2014;35(3):413-431.
29. Anderson J. Reported Diagnosis and Prescription Utilization Related to Attention Deficit Hyperactivity Disorder in Children Ages 5-17, 2008-2015. *Agency for Healthcare Research and Quality (US).* 2001.
30. Holden SE, Jenkins-Jones S, Poole CD, Morgan CL, Coghill D, Currie CJ. The prevalence and incidence, resource use and financial costs of treating people with attention deficit/hyperactivity disorder (ADHD) in the united kingdom (1998 to 2010). *Child and Adolescent Psychiatry and Mental Health.* 2013;7 (1) (no pagination)(34).
31. Nyarko KA, Grosse SD, Danielson ML, Holbrook JR, Visser SN, Shapira SK. Treated Prevalence of Attention-Deficit/Hyperactivity Disorder Increased from 2009 to 2015 among School-Aged Children and Adolescents in the United States. *Journal of Child and Adolescent Psychopharmacology.* 2017;27(8):731-734.
32. Bax AC, Bard DE, Cuffe SP, McKeown RE, Wolraich ML. The Association Between Race/Ethnicity and Socioeconomic Factors and the Diagnosis and Treatment of Children with Attention-Deficit Hyperactivity Disorder. *Journal of Developmental and Behavioral Pediatrics.* 2019;40(2):81-91.
33. Morgan PL, Staff J, Hillemeier MM, Farkas G, Maczuga S. Racial and ethnic disparities in ADHD diagnosis from kindergarten to eighth grade. *Pediatrics.* 2013;132(1):85-93.
34. Owens J, Jackson H. Attention-deficit/hyperactivity disorder severity, diagnosis, & later academic achievement in a national sample. *Social ScienceRresearch.* 2017;61:251-265.
35. Brownell MD, Yogendran MS. Attention-deficit hyperactivity disorder in Manitoba children: Medical diagnosis and psychostimulant treatment rates. *Canadian Journal of Psychiatry.* 2001;46(3):264-272.
36. Getahun D, Jacobsen SJ, Fassett MJ, Chen W, Demissie K, Rhoads GG. Recent trends in childhood attention-deficit/hyperactivity disorder. *JAMA Pediatrics.* 2013;167(3):282-288.
37. Coker TR, Elliott MN, Toomey SL, et al. Racial and Ethnic Disparities in ADHD Diagnosis and Treatment. *Pediatrics.* 2016;138(3).
38. Reyes N, Baumgardner DJ, Simmons DH, Buckingham W. The potential for sociocultural factors in the diagnosis of ADHD in children. *Wisconsin Medical Journal.* 2013;112(1):13-17.
39. Bonati M, Cartabia M, Zanetti M, et al. Age level vs grade level for the diagnosis of ADHD and neurodevelopmental disorders. *European Child and Adolescent Psychiatry.* 2018;27(9):1171-1180.
40. Elder TE. The importance of relative standards in ADHD diagnoses: evidence based on exact birth dates. *Journal of Health Economics.* 2010;29(5):641-656.

41. Evans WN, Morrill MS, Parente ST. Measuring inappropriate medical diagnosis and treatment in survey data: The case of ADHD among school-age children. *Journal of Health Economics.* 2010;29(5):657-673.
42. Halldner L, Tillander A, Lundholm C, et al. Relative immaturity and ADHD: Findings from nationwide registers, parent- and self-reports. *Journal of Child Psychology and Psychiatry.* 2014;55(8):897-904.
43. Holland J, Sayal K. Relative age and ADHD symptoms, diagnosis and medication: a systematic review. *European Child and Adolescent Psychiatry.* 2018.
44. Karlstad O, Furu K, Stoltenberg C, Haberg SE, Bakken IJ. ADHD treatment and diagnosis in relation to children's birth month: Nationwide cohort study from Norway. *Scandinavian Journal of Public Health.* 2017;45(4):343-349.
45. Layton TJ, Barnett ML, Hicks TR, Jena AB. Attention deficit-hyperactivity disorder and month of school enrollment. *New England Journal of Medicine.* 2018;379(22):2122-2130.
46. Morrow RL, Garland E, Wright JM, Maclure M, Taylor S, Dormuth CR. Influence of relative age on diagnosis and treatment of attention-deficit/hyperactivity disorder in children. *Canadian Medical Association Journal.* 2012;184(7):755-762.
47. Sayal K, Chudal R, Hinkka-Yli-Salomaki S, Joelsson P, Sourander A. Relative age within the school year and diagnosis of attention-deficit hyperactivity disorder: a nationwide population-based study. *The Lancet Psychiatry.* 2017;4(11):868-875.
48. Schwandt H, Wuppermann A. The youngest get the pill: ADHD misdiagnosis in Germany, its regional correlates and international comparison. *Labour Economics.* 2016;43:72-86.
49. Whitely M, Raven M, Timimi S, et al. Attention deficit hyperactivity disorder late birthdate effect common in both high and low prescribing international jurisdictions: a systematic review. *Journal of Child Psychology and Psychiatry and Allied Disciplines.* 2019;60(4):380-391.
50. Dalsgaard S, Humlum MK, Nielsen HS, Simonsen M. Relative standards in ADHD diagnoses: The role of specialist behavior. *Economics Letters.* 2012;117(3):663-665.
51. Lahey BB, Loeber R, Stouthamer-Loeber M, et al. Comparison of DSM-III and DSM-III-R diagnoses for prepubertal children: Changes in prevalence and validity. *J Am Acad Child Psy.* 1990;29(4):620-626.
52. Newcorn J, Halperin JM, Healey JM, et al. Are ADDH and ADHD the same or different? *J Am Acad Child Psy.* 1989;28(5):734-738.
53. Newcorn JH, Halperin JM, Schwartz S, et al. Parent and teacher ratings of attention-deficit hyperactivity disorder symptoms: implications for case identification. *Journal of Developmental and Behavioral Pediatrics.* 1994;15(2):86-91.
54. Wolraich ML, Hannah JN, Pinnock TY, Baumgaertel A, Brown J. Comparison of diagnostic criteria for attention-deficit hyperactivity disorder in a county-wide sample. *J Am Acad Child Psy.* 1996;35(3):319-324.
55. Ghanizadeh A. Agreement between diagnostic and statistical manual of mental disorders, Fourth Edition, and the proposed DSM-V attention deficit hyperactivity disorder diagnostic criteria: An exploratory study. *Comprehensive Psychiatry.* 2013;54(1):7-10.
56. McKeown RE, Holbrook JR, Danielson ML, Cuffe SP, Wolraich ML, Visser SN. The Impact of Case Definition on Attention-Deficit/Hyperactivity Disorder Prevalence Estimates in Community-Based Samples of School-Aged Children. *J Am Acad Child Psy.* 2015;54(1):53-61.
57. Baumgaertel A, Wolraich ML, Dietrich M. Comparison of diagnostic criteria for attention deficit disorders in a German elementary school sample. *J Am Acad Child Psy.* 1995;34(5):629-638.
58. Lahey BB, Applegate B, McBurnett K, et al. DSM-IV field trials for attention deficit hyperactivity disorder in children and adolescents. *American Journal of Psychiatry.* 1994;151(11):1673-1685.
59. Polanczyk GV, Willcutt EG, Salum GA, Kieling C, Rohde LA. ADHD prevalence estimates across three decades: An updated systematic review and meta-regression analysis. *International Journal of Epidemiology.* 2014;43(2):434-442.
60. Thomas R, Sanders S, Doust J, Beller E, Glasziou P. Prevalence of attention-deficit/hyperactivity disorder: a systematic review and meta-analysis. *Pediatrics.* 2015;135(4):e994-1001.
61. Fabiano F, Haslam N. Diagnostic inflation in the DSM: A meta-analysis of changes in the stringency of psychiatric diagnosis from DSM-III to DSM-5. *Clinical Psychology Review.* 2020:101889.

62. Leung PW, Luk SL, Ho TP, Taylor E, Mak FL, Bacon-Shone J. The diagnosis and prevalence of hyperactivity in Chinese schoolboys. *Br J Psychiatry.* 1996;168(4):486-496.
63. Dopfner M, Breuer D, Wille N, Erhart M, Ravens-Sieberer U. How often do children meet ICD-10/DSM-IV criteria of attention deficit-/hyperactivity disorder and hyperkinetic disorder? Parent-based prevalence rates in a national sample - Results of the BELLA study. *European Child and Adolescent Psychiatry.* 2008;17(SUPPL. 1):59-70.
64. Lahey BB, Pelham WE, Chronis A, et al. Predictive validity of ICD-10 hyperkinetic disorder relative to DSM-IV attention-deficit/hyperactivity disorder among younger children. *Journal of Child Psychology and Psychiatry.* 2006;47(5):472-479.
65. Lee SI, Schachar RJ, Chen SX, et al. Predictive validity of DSM-IV and ICD-10 criteria for ADHD and hyperkinetic disorder. *Journal of Child Psychology and Psychiatry and Allied Disciplines.* 2008;49(1):70-78.
66. Peyre H, Hoertel N, Cortese S, et al. Attention-deficit/hyperactivity disorder symptom expression: a comparison of individual age at onset using item response theory. *Journal of Clinical Psychiatry.* 2014;75(4):386-392.
67. Vande Voort JL, He JP, Jameson ND, Merikangas KR. Impact of the DSM-5 attention-deficit/hyperactivity disorder age-of-onset criterion in the us adolescent population. *J Am Acad Child Psy.* 2014;53(7):736-744.
68. Polanczyk G, Caspi A, Houts R, Kollins SH, Rohde LA, Moffitt TE. Implications of Extending the ADHD Age-of-Onset Criterion to Age 12: Results from a Prospectively Studied Birth Cohort. *J Am Acad Child Psy.* 2010;49(3):210-216.
69. Sibley MH, Rohde LA, Swanson JM, et al. Late-onset ADHD reconsidered with comprehensive repeated assessments between ages 10 and 25. *American Journal of Psychiatry.* 2018;175(2):140-149.
70. Malacrida C. Medicalization, ambivalence and social control: Mothers' descriptions of educators and ADD/ADHD. *Health.* 2004;8(1):61-80.
71. Mann EM, Ikeda Y, Mueller CW, et al. Cross-cultural differences in rating hyperactive-disruptive behaviors in children. *American Journal of Psychiatry.* 1992;149(11):1539-1542.
72. Rydell M, Lundstrom S, Gillberg C, Lichtenstein P, Larsson H. Has the attention deficit hyperactivity disorder phenotype become more common in children between 2004 and 2014? Trends over 10 years from a Swedish general population sample. *Journal of Child Psychology and Psychiatry.* 2018;59(8):863-871.
73. Safer DJ. Is ADHD Really Increasing in Youth? *Journal of Attention Disorders.* 2018;22(2):107-115.
74. Sawyer MG, Reece CE, Sawyer AC, Johnson SE, Lawrence D. Has the prevalence of child and adolescent mental disorders in Australia changed between 1998 and 2013 to 2014? *J Am Acad Child Psy.* 2018;57(5):343-350.
75. Wolraich ML, Hannah JN, Baumgaertel A, Feurer ID. Examination of DSM-IV criteria for attention deficit/hyperactivity disorder in a county-wide sample. *Journal of Developmental and Behavioral Pediatrics.* 1998;19(3):162-168.
76. Sellers R, Maughan B, Pickles A, Thapar A, Collishaw S. Trends in parent- and teacher-rated emotional, conduct and ADHD problems and their impact in prepubertal children in Great Britain: 1999-2008. *Journal of Child Psychology and Psychiatry and Allied Disciplines.* 2015;56(1):49-57.
77. Bruchmuller K, Margraf J, Schneider S. Is ADHD diagnosed in accord with diagnostic criteria? Overdiagnosis and influence of client gender on diagnosis. *Journal of Consulting and Clinical Psychology.* 2012;80(1):128-138.
78. Longridge R, Norman S, Henley W, Newlove Delgado T, Ford T. Investigating the agreement between the clinician and research diagnosis of attention deficit hyperactivity disorder and how it changes over time; a clinical cohort study. *Child and Adolescent Mental Health.* 2019;24(2):133-141.
79. Madsen KB, Ravn MH, Arnfred J, Olsen J, Rask CU, Obel C. Characteristics of undiagnosed children with parent-reported ADHD behaviour. *European Child and Adolescent Psychiatry.* 2018;27(2):149-158.
80. Okumura Y, Yamasaki S, Ando S, et al. Psychosocial Burden of Undiagnosed Persistent ADHD Symptoms in 12-Year-Old Children: A Population-Based Birth Cohort Study. *Journal of Attention Disorders.* 2019:1087054719837746.

©2021 Kazda L et al. *JAMA Network Open*

81. Loughran SB. Agreement and Stability of Teacher Rating Scales for Assessing ADHD in Preschoolers. *Early Childhood Education Journal.* 2003;30(4):247-253.
82. Rowland AS, Umbach DM, Catoe KE, et al. Studying the epidemiology of attention-deficit hyperactivity disorder: Screening method and pilot results. *Canadian Journal of Psychiatry.* 2001;46(10):931-940.
83. Fabiano GA, Pelham WE, Jr., Majumdar A, et al. Elementary and middle school teacher perceptions of attention-deficit/hyperactivity disorder prevalence. *Child and Youth Care Forum.* 2013;42(2):87-99.
84. Foreman DM, Ford T. Assessing the diagnostic accuracy of the identification of hyperkinetic disorders following the introduction of government guidelines in England. *Child and Adolescent Psychiatry and Mental Health.* 2008;2.
85. Balazs J, Kereszteny A. Subthreshold attention deficit hyperactivity in children and adolescents: a systematic review. *European Child and Adolescent Psychiatry.* 2014;23(6):393-408.
86. Biederman J, Fitzgerald M, Kirova AM, Woodworth KY, Biederman I, Faraone SV. Further Evidence of Morbidity and Dysfunction Associated With Subsyndromal ADHD in Clinically Referred Children. *Journal of Clinical Psychiatry.* 2018;79(5):07.
87. Cho SC, Kim BN, Kim JW, et al. Full syndrome and subthreshold attention-deficit/hyperactivity disorder in a Korean community sample: Comorbidity and temperament findings. *European Child and Adolescent Psychiatry.* 2009;18(7):447-457.
88. Fergusson DM, Boden JM, Horwood L. Classification of behavior disorders in adolescence: Scaling methods, predictive validity, and gender differences. *Journal of Abnormal Psychology.* 2010;119(4):699-712.
89. Fergusson DM, Horwood L. Predictive validity of categorically and dimensionally scored measures of disruptive childhood behaviors. *J Am Acad Child Psy.* 1995;34(4):477-485.
90. Fergusson DM, Lynskey MT, Horwood LJ. Attentional difficulties in middle childhood and psychosocial outcomes in young adulthood. *Journal of Child Psychology and Psychiatry and Allied Disciplines.* 1997;38(6):633-644.
91. Hong SB, Dwyer D, Kim JW, et al. Subthreshold attention-deficit/hyperactivity disorder is associated with functional impairments across domains: A comprehensive analysis in a large-scale community study. *European Child and Adolescent Psychiatry.* 2014;23(8):627-636.
92. Kirova AM, Kelberman C, Storch B, et al. Are subsyndromal manifestations of attention deficit hyperactivity disorder morbid in children? A systematic qualitative review of the literature with meta-analysis. *Psychiatry Research.* 2019;274:75-90.
93. Merrell C, Sayal K, Tymms P, Kasim A. A longitudinal study of the association between inattention, hyperactivity and impulsivity and children's academic attainment at age 11. *Learning and Individual Differences.* 2017;53:156-161.
94. Noren Selinus E, Molero Y, Lichtenstein P, et al. Subthreshold and threshold attention deficit hyperactivity disorder symptoms in childhood: psychosocial outcomes in adolescence in boys and girls. *Acta Psychiatrica Scandinavica.* 2016;134(6):533-545.
95. Sayal K, Washbrook E, Propper C. Childhood behavior problems and academic outcomes in adolescence: Longitudinal population-based study. *J Am Acad Child Psy.* 2015;54(5):360-368.e362.
96. Schmiedeler S, Schneider W. Attention-deficit hyperactivity disorder (ADHD) in the early years: diagnostic issues and educational relevance. *Clinical Child Psychology and Psychiatry.* 2014;19(3):460-475.
97. Whalen CK, Jamner LD, Henker B, Delfino RJ, Lozano JM. The ADHD spectrum and everyday life: experience sampling of adolescent moods, activities, smoking, and drinking. *Child Development.* 2002;73(1):209-227.
98. Larsson H, Anckarsater H, Rastam M, Chang Z, Lichtenstein P. Childhood attention-deficit hyperactivity disorder as an extreme of a continuous trait: a quantitative genetic study of 8,500 twin pairs. *Journal of Child Psychology and Psychiatry and Allied Disciplines.* 2012;53(1):73-80.
99. Lecendreux M, Konofal E, Cortese S, Faraone SV. A 4-Year follow-up of attention-deficit/hyperactivity disorder in a population sample. *Journal of Clinical Psychiatry.* 2015;76(6):712-719.

100. Lecendreux M, Silverstein M, Konofal E, Cortese S, Faraone SV. A 9-Year Follow-Up of Attention-Deficit/Hyperactivity Disorder in a Population Sample. *Journal of Clinical Psychiatry.* 2019;80(3).
101. Haslam N, Williams B, Prior M, Haslam R, Graetz B, Sawyer M. The latent structure of attention-deficit/hyperactivity disorder: A taxometric analysis. *Australian and New Zealand Journal of Psychiatry.* 2006;40(8):639-647.
102. Hudziak JJ, Heath AC, Madden PF, et al. Latent class and factor analysis of DSM-IV ADHD: A twin study of female adolescents. *J Am Acad Child Psy.* 1998;37(8):848-857.
103. Levy F, Hay DA, McStephen M, Wood C, Waldman I. Attention-deficit hyperactivity disorder: A category or a continuum? Genetic analysis of a large-scale twin study. *J Am Acad Child Psy.* 1997;36(6):737-744.
104. Lubke GH, Hudziak JJ, Derks EM, van Bijsterveldt TC, Boomsma DI. Maternal ratings of attention problems in ADHD: evidence for the existence of a continuum. *J Am Acad Child Psy.* 2009;48(11):1085-1093.
105. Marcus DK, Barry TD. Does Attention-Deficit/Hyperactivity Disorder Have a Dimensional Latent Structure? A Taxometric Analysis. *Journal of Abnormal Psychology.* 2011;120(2):427-442.
106. McLennan JD. Understanding attention deficit hyperactivity disorder as a continuum. *Can Fam Physician.* 2016;62(12):979-982.
107. Atladottir HO, Gyllenberg D, Langridge A, et al. The increasing prevalence of reported diagnoses of childhood psychiatric disorders: a descriptive multinational comparison. *European Child and Adolescent Psychiatry.* 2015;24(2):173-183.
108. Fulton BD, Scheffler RM, Hinshaw SP. State variation in increased adhd prevalence: Links to NCLB school accountability and state medication laws. *Psychiatric Services.* 2015;66(10):1074-1082.
109. Garfield CF, Dorsey ER, Zhu S, et al. Trends in attention deficit hyperactivity disorder ambulatory diagnosis and medical treatment in the United States, 2000-2010. *Academic Pediatrics.* 2012;12(2):110-116.
110. Giacobini M, Medin E, Ahnemark E, Russo LJ, Carlqvist P. Prevalence, Patient Characteristics, and Pharmacological Treatment of Children, Adolescents, and Adults Diagnosed With ADHD in Sweden. *Journal of Attention Disorders.* 2018;22(1):3-13.
111. Hoagwood KE, Kelleher K, Zima BT, Perrin JM, Bilder S, Crystal S. Ten-Year Trends In Treatment Services For Children With Attention Deficit Hyperactivity Disorder Enrolled In Medicaid. *Health Affairs.* 2016;35(7):1266-1270.
112. Langner I, Haug U, Scholle O, Lindemann C, Schroder C, Riedel O. Potential Explanations for Increasing Methylphenidate Use in Children and Adolescents with Attention-Deficit/Hyperactivity Disorder in Germany from 2004 to 2013. *Journal of Clinical Psychopharmacology.* 2019;39(1):39-45.
113. Robison LM, Sclar DA, Skaer TL, Galin RS. National trends in the prevalence of attention-deficit/hyperactivity disorder and the prescribing of methylphenidate among school-age children: 1990-1995. *Clinical Pediatrics.* 1999;38(4):209-217.
114. Song I, Lee MS, Lee EK, Shin JY. Patient and provider characteristics related with prescribing of ADHD medication: Nationwide health insurance claims database study in Korea. *Asia-Pacific Psychiatry.* 2018;10 (1) (no pagination)(e12289).
115. Song I, Shin JY. Prescribing patterns for attention deficit hyperactivity disorder medications among children and adolescents in Korea, 2007-2011. *Epidemiology and Health.* 2016;38:e2016045.
116. Song M, Dieckmann NF, Nigg JT. Addressing Discrepancies Between ADHD Prevalence and Case Identification Estimates Among U.S. Children Utilizing NSCH 2007-2012. *Journal of Attention Disorders.* 2018:1087054718799930.
117. Toh S. Trends in ADHD and stimulant use among children, 1993-2003. *Psychiatric Services.* 2006;57(8):1091.
118. Vasiliadis HM, Diallo FB, Rochette L, et al. Temporal Trends in the Prevalence and Incidence of Diagnosed ADHD in Children and Young Adults between 1999 and 2012 in Canada: A Data Linkage Study. *Canadian Journal of Psychiatry.* 2017;62(12):818-826.
119. Wang L, Lee S, Yuan S, et al. Prevalence rates of youths diagnosed with and medicated for ADHD in a nationwide survey in Taiwan from 2000 to 2011. *Epidemiology and Psychiatric Sciences.* 2017;26(6):624-634.

©2021 Kazda L et al. *JAMA Network Open*

120. Winterstein AG, Gerhard T, Shuster J, et al. Utilization of pharmacologic treatment in youths with attention deficit/hyperactivity disorder in Medicaid database. *Annals of Pharmacotherapy*. 2008;42(1):24-31.
121. Yoshida M, Obara T, Kikuchi S, et al. Drug Prescriptions for Children With ADHD in Japan: A Study Based on Health Insurance Claims Data Between 2005 and 2015. *Journal of Attention Disorders*. 2019:1087054719843179.
122. Zito JM, Safer DJ, DosReis S, Magder LS, Gardner JF, Zarin DA. Psychotherapeutic medication patterns for youths with attention- deficit/hyperactivity disorder. *Archives of Pediatrics and Adolescent Medicine*. 1999;153(12):1257-1263.
123. Sclar DA, Robison LM, Bowen KA, Schmidt JM, Castillo LV, Oganov AM. Attention-deficit/hyperactivity disorder among children and adolescents in the United States: Trend in diagnosis and use of pharmacotherapy by gender. *Clinical Pediatrics*. 2012;51(6):584-589.
124. Huang CL, Wang JJ, Ho CH. Trends in incidence rates of diagnosed attention-deficit/hyperactivity disorder (ADHD) over 12 years in Taiwan: A nationwide population-based study. *Psychiatry Research*. 2020;284:112792.
125. Mohr Jensen C, Steinhausen HC. Time trends in incidence rates of diagnosed attention-deficit/hyperactivity disorder across 16 years in a nationwide Danish registry study. *Journal of Clinical Psychiatry*. 2015;76(3):e334-341.
126. Perez-Crespo L, Canals-Sans J, Suades-Gonzalez E, Guxens M. Temporal trends and geographical variability of the prevalence and incidence of attention deficit/hyperactivity disorder diagnoses among children in Catalonia, Spain. *Scientific Reports*. 2020;10(1):6397.
127. Stuhec M, Svab V, Locatelli I. Prevalence and incidence of attention-deficit/hyperactivity disorder in Slovenian children and adolescents: A database study from a national perspective. *Croatian Medical Journal*. 2015;56(2):159-165.
128. Chien IC, Lin CH, Chou YJ, Chou P. Prevalence, incidence, and stimulant use of attention-deficit hyperactivity disorder in Taiwan, 1996-2005: a national population-based study. *Soc Psychiatry Psychiatr Epidemiol*. 2012;47(12):1885-1890.
129. Dalsgaard S, Nielsen HS, Simonsen M. Consequences of ADHD medication use for children's outcomes. *Journal of Health Economics*. 2014;37:137-151.
130. Santosh PJ, Taylor E, Swanson J, et al. Refining the diagnoses of inattention and overactivity syndromes: A reanalysis of the Multimodal Treatment study of attention deficit hyperactivity disorder (ADHD) based on ICD-10 criteria for hyperkinetic disorder. *Clinical Neuroscience Research*. 2005;5(5-6):307-314.
131. Donfrancesco R, Marano A, Calderoni D, et al. Prevalence of severe ADHD: An epidemiological study in the Italian regions of Tuscany and Latium. *Epidemiology and Psychiatric Sciences*. 2015;24(6):525-533.
132. Bachmann CJ, Wijlaars LP, Kalverdijk LJ, et al. Trends in ADHD medication use in children and adolescents in five western countries, 2005-2012. *Eur Neuropsychopharmacol*. 2017;27(5):484-493.
133. Barczyk ZA, Rucklidge JJ, Eggleston M, Mulder RT. Psychotropic Medication Prescription Rates and Trends for New Zealand Children and Adolescents 2008-2016. *Journal of Child and Adolescent Psychopharmacology*. 2020;30(2):87-96.
134. Beau-Lejdstrom R, Douglas I, Evans SJW, Smeeth L. Latest trends in ADHD drug prescribing patterns in children in the UK: Prevalence, incidence and persistence. *BMJ Open*. 2016;6 (6) (no pagination)(010508).
135. Boland F, Galvin R, Reulbach U, et al. Psychostimulant prescribing trends in a paediatric population in Ireland: a national cohort study. *BMC Pediatrics*. 2015;15:118.
136. Burcu M, Zito J, Metcalfe L. Trends in stimulant medication use in commercially insured youths and adults, 2010-2014. *JAMA Psychiatry*. 2016;73(9).
137. Castle L, Aubert RE, Verbrugge RR, Khalid M, Epstein RS. Trends in medication treatment for ADHD. *Journal of Attention Disorders*. 2007;10(4):335-342.
138. Dalsgaard S, Nielsen HS, Simonsen M. Five-fold increase in national prevalence rates of attention-deficit/ hyperactivity disorder medications for children and adolescents with autism spectrum disorder, attention-deficit/hyperactivity disorder, and other psychiatric disorders: A danish register-based study. *Journal of Child and Adolescent Psychopharmacology*. 2013;23(7):432-439.

139. Fogelman Y, Vinker S, Guy N, Kahan E. Prevalence of and change in the prescription of methylphenidate in Israel over a 2-year period. *CNS Drugs.* 2003;17(12):915-919.
140. Fullerton CA, Epstein AM, Frank RG, Normand SL, Fu CX, McGuire TG. Medication use and spending trends among children with ADHD in Florida's Medicaid program, 1996-2005. *Psychiatric Services.* 2012;63(2):115-121.
141. Furu K, Karlstad O, Zoega H, et al. Utilization of Stimulants and Atomoxetine for Attention-Deficit/Hyperactivity Disorder among 5.4 Million Children Using Population-Based Longitudinal Data. *Basic and Clinical Pharmacology and Toxicology.* 2017;120(4):373-379.
142. Grimmsmann T, Himmel W. The 10-year trend in drug prescriptions for attention-deficit/hyperactivity disorder (ADHD) in Germany. *European Journal of Clinical Pharmacology.* 2020;17:17.
143. Gumy C, Huissoud T, Dubois-Arber F. Prevalence of methylphenidate prescription among school-aged children in a swiss population: Increase in the number of prescriptions in the swiss canton of vaud, from 2002 to 2005, and changes in patient demographics. *Journal of Attention Disorders.* 2010;14(3):267-272.
144. Habel LA, Schaefer CA, Levine P, Bhat AK, Elliott G. Treatment with stimulants among youths in a large California health plan. *Journal of Child and Adolescent Psychopharmacology.* 2005;15(1):62-67.
145. Hodgkins P, Sasane R, Meijer WM. Pharmacologic Treatment of Attention-Deficit/Hyperactivity Disorder in Children: Incidence, Prevalence, and Treatment Patterns in The Netherlands. *Clinical Therapeutics.* 2011;33(2):188-203.
146. Hugtenburg J, Heerdink E, Egberts A. Increased psychotropic drug consumption by children in the Netherlands during 1995–2001 is caused by increased use of methylphenidate by boys. *European Journal of Clinical Pharmacology.* 2004;60.
147. Jaber L, Rigler S, Shuper A, Diamond G. Changing Epidemiology of Methylphenidate Prescriptions in the Community: A Multifactorial Model. *Journal of Attention Disorders.* 2017;21(14):1143-1150.
148. Johansen ME, Matic K, McAlearney AS. Attention Deficit Hyperactivity Disorder Medication Use among Teens and Young Adults. *Journal of Adolescent Health.* 2015;57(2):192-197.
149. Karanges EA, Stephenson CP, McGregor IS. Longitudinal trends in the dispensing of psychotropic medications in Australia from 2009-2012: Focus on children, adolescents and prescriber specialty. *Australian and New Zealand Journal of Psychiatry.* 2014;48(10):917-931.
150. Knellwolf A-L, Deligne J, Chiarotti F, et al. Prevalence and patterns of methylphenidate use in French children and adolescents. *European Journal of Clinical Pharmacology.* 2008;64(3).
151. Lillemoen PK, Kjosavik SR, Hunskar S, Ruths S. Prescriptions for ADHD medication, 2004-08. *Tidsskrift for den Norske laegeforening.* 2012;132(16):1856-1860.
152. Lopez-Leon S, Lopez-Gomez MI, Warner B, Ruiter-Lopez L. Psychotropic medication in children and adolescents in the United States in the year 2004 vs 2014. *DARU, Journal of Pharmaceutical Sciences.* 2018;26(1):5-10.
153. Man KK, Ip P, Hsia Y, et al. ADHD drug prescribing trend is increasing among children and adolescents in Hong Kong. *Journal of Attention Disorders.* 2017;21(14):1161-1168.
154. McCarthy S, Wilton L, Murray ML, Hodgkins P, Asherson P, Wong IC. The epidemiology of pharmacologically treated attention deficit hyperactivity disorder (ADHD) in children, adolescents and adults in UK primary care. *BMC Pediatrics.* 2012;12:78.
155. Olfson M, Gameroff MJ. National trends in the treatment of attention deficit hyperactivity disorder. *American Journal of Psychiatry.* 2003;160(6):1071-1077.
156. Raman SR, Man KK, Bahmanyar S, et al. Trends in attention-deficit hyperactivity disorder medication use: A retrospective observational study using population-based databases. *The Lancet Psychiatry.* 2018;5(10):824-835.
157. Renoux C, Shin JY, Dell'Aniello S, Fergusson E, Suissa S. Prescribing trends of attention-deficit hyperactivity disorder (ADHD) medications in UK primary care, 1995-2015. *British Journal of Clinical Pharmacology.* 2016.
158. Romano E, Baillargeon RH, Wu HX, Robaey P, Tremblay RE. Prevalence of methylphenidate use and change over a two-year period: A nationwide study of 2- to 11-year-old Canadian children. *Journal of Pediatrics.* 2002;141(1):71-75.
159. Safer DJ, Krager JM. Trends in medication treatment of hyperactive school children. Results of six biannual surveys. *Clinical Pediatrics.* 1983;22(7):500-504.

160. Safer DJ, Krager JM. Trends in medication therapy for hyperactivity: National and international perspectives. *Advances in Learning & Behavioral Disabilities*. 1984;3:125-149.
161. Safer DJ, Krager JM. The increased rate of stimulant treatment for hyperactive/inattentive students in secondary schools. *Pediatrics*. 1994;94(4 Pt 1):462-464.
162. Salmelainen P. Trends in the prescribing of stimulant medication for the treatment of attention deficit hyperactivity disorder in children and adolescents in New South Wales. *New South Wales Public Health Bulletin*. 2002;13 Suppl S-1:1-65.
163. Trip AM, Visser ST, Kalverdijk LJ, De Jong-Van Den Berg LTW. Large increase of the use of psycho-stimulants among youth in the Netherlands between 1996 and 2006. *British Journal of Clinical Pharmacology*. 2009;67(4):466-468.
164. Valentine J, Zubrick S, Sly P. National trends in the use of stimulant medication for attention deficit hyperactivity disorder. *Journal of Paediatrics and Child Health*. 1996;32(3):223-227.
165. Vinker S, Vinker R, Elhayany A. Prevalence of methylphenidate use among Israeli children: 1998-2004. *Clinical Drug Investigation*. 2006;26(3):161-167.
166. Vuori M, Koski-Pirila A, Martikainen JE, Saastamoinen L. Gender- and age-stratified analyses of ADHD medication use in children and adolescents in Finland using population-based longitudinal data, 2008-2018. *Scandinavian Journal of Public Health*. 2020;48(3):303-307.
167. Wang LJ, Lee SY, Yuan SS, et al. Impact of negative media publicity on attention-deficit/hyperactivity disorder medication in Taiwan. *Pharmacoepidemiology and Drug Safety*. 2016;25(1):45-53.
168. Yoon EY, Cohn L, Rocchini A, Kershaw D, Clark SJ. Clonidine utilization trends for Medicaid children. *Clinical Pediatrics*. 2012;51(10):950-955.
169. Zetterqvist J, Asherson P, Halldner L, Langstrom N, Larsson H. Stimulant and non-stimulant attention deficit/hyperactivity disorder drug use: Total population study of trends and discontinuation patterns 2006-2009. *Acta Psychiatrica Scandinavica*. 2013;128(1):70-77.
170. Zuvekas SH, Vitiello B, Norquist GS. Recent trends in stimulant medication use among U.S. children. *American Journal of Psychiatry*. 2006;163(4):579-585.
171. Zito JM, Safer DJ, dosReis S, Gardner JF, Boles M, Lynch F. Trends in the prescribing of psychotropic medications to preschoolers. *Jama*. 2000;283(8):1025-1030.
172. Morkem R, Patten S, Queenan J, Barber D. Recent Trends in the Prescribing of ADHD Medications in Canadian Primary Care. *Journal of Attention Disorders*. 2017:1087054717720719.
173. Davis DW, Feygin Y, Creel L, et al. Longitudinal Trends in the Diagnosis of Attention-Deficit/Hyperactivity Disorder and Stimulant Use in Preschool Children on Medicaid. *Journal of Pediatrics*. 2019;207:185-191.e181.
174. Zuvekas SH, Vitiello B. Stimulant medication use in children: a 12-year perspective. *American Journal of Psychiatry*. 2012;169(2):160-166.
175. Janols LO, Liliemark J, Klintberg K, von Knorring AL. Central stimulants in the treatment of attention-deficit hyperactivity disorder (ADHD) in children and adolescents. A naturalistic study of the prescription in Sweden, 1977-2007. *Nord J Psychiatry*. 2009;63(6):508-516.
176. Van Den Ban E, Souverein P, Swaab H, Van Engeland H, Heerdink R, Egberts T. Trends in incidence and characteristics of children, adolescents, and adults initiating immediate- or extended-release methylphenidate or atomoxetine in The Netherlands during 2001-2006. *Journal of Child and Adolescent Psychopharmacology*. 2010;20(1):55-61.
177. Jick H, Kaye JA, Black C. Incidence and prevalence of drug-treated attention deficit disorder among boys in the UK. *British Journal of General Practice*. 2004;54(502):345-347.
178. Prosser B, Lambert MC, Reid R. Psychostimulant prescription for ADHD in new South Wales: a longitudinal perspective. *Journal of Attention Disorders*. 2015;19(4):284-292.
179. Prosser B, Reid R. Changes in use of psychostimulant medication for ADHD in South Australia (1990-2006). *Australian and New Zealand Journal of Psychiatry*. 2009;43(4):340-347.
180. Alessi-Severini S, Biscontri RG, Collins DM, Sareen J, Enns MW. Ten years of antipsychotic prescribing to children: a Canadian population-based study. *Canadian Journal of Psychiatry*. 2012;57(1):52-58.
181. Bruckner TA, Hodgson A, Mahoney CB, Fulton BD, Levine P, Scheffler RM. Health care supply and county-level variation in attention-deficit hyperactivity disorder prescription medications. *Pharmacoepidemiology and Drug Safety*. 2012;21(4):442-449.

182. Chai G, Governale L, McMahon AW, Trinidad JP, Staffa J, Murphy D. Trends of outpatient prescription drug utilization in US children, 2002-2010. *Pediatrics.* 2012;130(1):23-31.
183. Hollingworth SA, Nissen LM, Stathis SS, Siskind DJ, Varghese JMN, Scott JG. Australian national trends in stimulant dispensing: 2002a" 2009. *Australian and New Zealand Journal of Psychiatry.* 2011;45(4):332-336.
184. Oner O, Yilmaz ES, Karadag H, et al. ADHD Medication Trends in Turkey: 2009-2013. *Journal of Attention Disorders.* 2017;21(14):1192-1197.
185. Ponizovsky AM, Marom E, Fitoussi I. Trends in attention deficit hyperactivity disorder drugs consumption, Israel, 2005-2012. *Pharmacoepidemiology and Drug Safety.* 2014;23(5):534-538.
186. Scheffler RM, Hinshaw SP, Modrek S, Levine P. The global market for ADHD medications. *Health Affairs.* 2007;26(2):450-457.
187. Stuhec M, Locatelli I, Svab V. Trends in Attention-Deficit/Hyperactivity Disorder Drug Consumption in Children and Adolescents in Slovenia from 2001 to 2012: A Drug Use Study from a National Perspective. *Journal of Child and Adolescent Psychopharmacology.* 2015;25(3):254-259.
188. Treceno C, Martin Arias LH, Sainz M, et al. Trends in the consumption of attention deficit hyperactivity disorder medications in Castilla y Leon (Spain): Changes in the consumption pattern following the introduction of extended release methylphenidate. *Pharmacoepidemiology and Drug Safety.* 2012;21(4):435-441.
189. Pottegard A, Bjerregaard BK, Glintborg D, Hallas J, Moreno SI. The use of medication against attention deficit hyperactivity disorder in Denmark: a drug use study from a national perspective. *European Journal of Clinical Pharmacology.* 2012;68(10):1443-1450.
190. Girand HL, Litkowiec S, Sohn M. Attention-deficit/hyperactivity disorder and psychotropic polypharmacy prescribing trends. *Pediatrics.* 2020;146 (1) (no pagination)(e20192832).
191. Thomas CP, Conrad P, Casler R, Goodman E. Trends in the use of psychotropic medications among adolescents, 1994 to 2001. *Psychiatric Services.* 2006;57(1):63-69.
192. Chirdkiatgumchai V, Xiao H, Fredstrom BK, et al. National trends in psychotropic medication use in young children: 1994-2009. *Pediatrics.* 2013;132(4):615-623.
193. Damico JS, Augustine LE. Social considerations in the labeling of students as attention deficit hyperactivity disordered. *Seminars in speech and language.* 1995;16(4):259-273; quiz 274.
194. Klasen H. A name, what's in a name? The medicalization of hyperactivity, revisited. *Harvard Review of Psychiatry.* 2000;7(6):334-344.
195. Moore D, Russell A, Arnell S, Ford T. Educators' experiences of managing students with ADHD: A qualitative study. *Child: Care, Health and Development.* 2017;43(4):489-498.
196. Rogalin MT, Nencini A. Consequences of the "Attention-Deficit/Hyperactivity Disorder" (ADHD) diagnosis. An investigation with education professionals. *Psychological Studies.* 2015;60(1):41-49.
197. Andersson Frondelius I, Ranjbar V, Danielsson L. Adolescents' experiences of being diagnosed with attention deficit hyperactivity disorder: a phenomenological study conducted in Sweden. *BMJ Open.* 2019;9(8):e031570.
198. Carr-Fanning K, Mc Guckin C. The powerless or the empowered? Stakeholders' experiences of diagnosis and treatment for attention-deficit hyperactivity disorder in Ireland. *Ir J Psychol Med.* 2018;35(3):203-212.
199. Hamed AM, Kauer AJ, Stevens HE. Why the diagnosis of attention deficit hyperactivity disorder matters. *Frontiers in Psychiatry.* 2015;6 (NOV) (no pagination)(168).
200. Wienen AW, Sluiter MN, Thoutenhoofd E, de Jonge P, Batstra L. The advantages of an ADHD classification from the perspective of teachers. *European Journal of Special Needs Education.* 2019.
201. Meza JI, Monroy M, Ma R, Mendoza-Denton R. Stigma and attention-deficit/hyperactivity disorder: negative perceptions and anger emotional reactions mediate the link between active symptoms and social distance. *Atten Defic Hyperact Disord.* 2019;27.
202. Comstock E. The rhetorical construction of the AD/HD subject: Managing the self. *Journal of Language, Identity and Education.* 2015;14(1):1-18.
203. Singh I. A disorder of anger and aggression: Children's perspectives on attention deficit/hyperactivity disorder in the UK. *Social Science and Medicine.* 2011;73(6):889-896.

204. Dryer R, Kiernan MJ, Tyson GA. The effects of diagnostic labelling on the implicit theories of attention-deficit/hyperactivity disorder held by health professionals. *Behaviour Change*. 2006;23(3):177-185.
205. Cornett-Ruiz S, Hendricks B. Effects of labeling and ADHD behaviors on peer and teacher judgments. *Journal of Educational Research*. 1993;86(6):349-355.
206. Ghanizadeh A, Fallahi M, Akhondzadeh S. Disclosure of attention deficit hyperactivity disorder and its effect on rejection of students by teachers. *Iranian Journal of Medical Sciences*. 2009;34(4):259-264.
207. Gibbs S, Beckmann JF, Elliott J, Metsapelto RL, Vehkakoski T, Aro M. What's in a name: the effect of category labels on teachers' beliefs. *European Journal of Special Needs Education*. 2020;35(1):115-127.
208. Law G, Sinclair S, Fraser N. Children's attitudes and behavioural intentions towards a peer with symptoms of ADHD: Does the addition of a diagnostic label make a difference? *Journal of Child Health Care*. 2007;11(2):98-111.
209. Metzger AN, Hamilton LT. The Stigma of ADHD: Teacher Ratings of Labeled Students. *Sociological Perspectives*. 2020:0731121420937773.
210. O'Connor C, McNicholas F. What Differentiates Children with ADHD Symptoms Who Do and Do Not Receive a Formal Diagnosis? Results from a Prospective Longitudinal Cohort Study. *Child Psychiatry Hum Dev*. 2020;51(1):138-150.
211. Owens J. Relationships between an ADHD Diagnosis and Future School Behaviors among Children with Mild Behavioral Problems. *Sociology of Education*. 2020;93(3):191-214.
212. Sayal K, Owen V, White K, Merrell C, Tymms P, Taylor E. Impact of early school-based screening and intervention programs for ADHD on children's outcomes and access to services: follow-up of a school-based trial at age 10 years. *Archives of Pediatrics and Adolescent Medicine*. 2010;164(5):462-469.
213. Ohan JL, Visser TA, Strain MC, Allen L. Teachers' and education students' perceptions of and reactions to children with and without the diagnostic label "ADHD". *J Sch Psychol*. 2011;49(1):81-105.
214. Stinnett TA, Crawford SA, Gillespie MD, Cruce MK, Langford CA. Factors affecting treatment acceptability for psychostimulant medication versus psychoeducational intervention. *Psychology in the Schools*. 2001;38(6):585-591.
215. DosReis S, Barksdale CL, Sherman A, Maloney K, Charach A. Stigmatizing experiences of parents of children with a new diagnosis of ADHD. *Psychiatric Services*. 2010;61(8):811-816.
216. Batzle CS, Weyandt LL, Janusis GM, DeVietti TL. Potential impact of adhd with stimulant medication label on teacher expectations. *Journal of Attention Disorders*. 2010;14(2):157-166.
217. Coleman D, Walker JS, Lee J, Friesen BJ, Squire PN. Children's beliefs about causes of childhood depression and ADHD: A study of stigmatization. *Psychiatric Services*. 2009;60(7):950-957.
218. Koonce DA, Cruce MK, Aldridge JO, Langford CA, Sporer AK, Stinnett TA. The ADHD label, analogue methodology, and participants' geographic location on judgments of social and attentional skills. *Psychology in the Schools*. 2004;41(2):221-234.
219. Ohan JL, Visser TA, Moss RG, Allen NB. Parents' stigmatizing attitudes toward psychiatric labels for ADHD and depression. *Psychiatric Services*. 2013;64(12):1270-1273.
220. Wang LJ, Chen CK, Huang YS. Neurocognitive performance and behavioral symptoms in patients with attention-deficit/hyperactivity disorder during twenty-four months of treatment with methylphenidate. *Journal of Child and Adolescent Psychopharmacology*. 2015;25(3):246-253.
221. Coghill DR, Seth S, Pedroso S, Usala T, Currie J, Gagliano A. Effects of methylphenidate on cognitive functions in children and adolescents with attention-deficit/hyperactivity disorder: evidence from a systematic review and a meta-analysis. *Biol Psychiatry*. 2014;76(8):603-615.
222. Schweren L, Hoekstra P, van Lieshout M, et al. Long-term effects of stimulant treatment on ADHD symptoms, social-emotional functioning, and cognition. *Psychological Medicine*. 2019;49(2):217-223.
223. Brossard-Racine M, Shevell M, Snider L, Belanger SA, Majnemer A. Motor skills of children newly diagnosed with Attention Deficit Hyperactivity Disorder prior to and following treatment with stimulant medication. *Research in Developmental Disabilities*. 2012;33(6):2080-2087.

©2021 Kazda L et al. *JAMA Network Open*

224. Fleming M, Fitton CA, Steiner MFC, et al. Educational and Health Outcomes of Children Treated for Attention-Deficit/Hyperactivity Disorder. *JAMA Pediatrics*. 2017;171(7):e170691.
225. Charles L, Schain R. A four-year follow-up study of the effects of methylphenidate on the behavior and academic achievement of hyperactive children. *J Abnorm Child Psychol.* 1981;9(4):495-505.
226. O'Connor B, Garner AA, Peugh JL, Simon J, Epstein JN. Improved but still impaired: Symptom-impairment correspondence among youth with attention-deficit hyperactivity disorder receiving community-based care. *Journal of Developmental and Behavioral Pediatrics*. 2015;36(2):106-114.
227. Van der Oord S, Prins PJ, Oosterlaan J, Emmelkamp PM. Efficacy of methylphenidate, psychosocial treatments and their combination in school-aged children with ADHD: a meta-analysis. *Clinical Psychology Review*. 2008;28(5):783-800.
228. Barbaresi WJ, Katusic SK, Colligan RC, Weaver AL, Jacobsen SJ. Modifiers of long-term school outcomes for children with attention-deficit/hyperactivity disorder: does treatment with stimulant medication make a difference? Results from a population-based study. *Journal of Developmental and Behavioral Pediatrics*. 2007;28(4):274-287.
229. Kortekaas-Rijlaarsdam AF, Luman M, Sonuga-Barke E, Oosterlaan J. Does methylphenidate improve academic performance? A systematic review and meta-analysis. *European Child and Adolescent Psychiatry*. 2019;28(2):155-164.
230. Langberg JM, Becker SP. Does Long-Term Medication Use Improve the Academic Outcomes of Youth with Attention-Deficit/Hyperactivity Disorder? *Clinical Child and Family Psychology Review*. 2012;15(3):215-233.
231. Prasad V, Brogan E, Mulvaney C, Grainge M, Stanton W, Sayal K. How effective are drug treatments for children with ADHD at improving on-task behaviour and academic achievement in the school classroom? A systematic review and meta-analysis. *European Child and Adolescent Psychiatry*. 2013;22(4):203-216.
232. Scheffler RM, Brown TT, Fulton BD, Hinshaw S, Levine P, Stone S. Positive association between attention-deficit/hyperactivity disorder medication use and academic achievement during elementary school. *Pediatrics*. 2009;123(5).
233. Zoega H, Rothman KJ, Huybrechts KF, et al. A population-based study of stimulant drug treatment of ADHD and academic progress in children. *Pediatrics*. 2012;130(1):e53-62.
234. Jangmo A, Stalhandske A, Chang Z, et al. Attention-Deficit/Hyperactivity Disorder, School Performance, and Effect of Medication. *J Am Acad Child Psy*. 2019;58(4):423-432.
235. Keilow M, Holm A, Fallesen P. Medical treatment of Attention Deficit/ Hyperactivity Disorder (ADHD) and children's academic performance. *PLoS ONE*. 2018;13 (11) (no pagination)(e0207905).
236. Currie J, Stabile M, Jones L. Do stimulant medications improve educational and behavioral outcomes for children with ADHD? *Journal of Health Economics*. 2014;37:58-69.
237. Van Den Ban E, Souverein P, Meijer W, et al. Association between ADHD drug use and injuries among children and adolescents. *European Child and Adolescent Psychiatry*. 2014;23(2):95-102.
238. Leibson CL, Barbaresi WJ, Ransom J, et al. Emergency department use and costs for youth with attention-deficit/hyperactivity disorder: Associations with stimulant treatment. *Ambulatory Pediatrics*. 2006;6(1):45-53.
239. Dalsgaard S, Leckman JF, Mortensen PB, Nielsen HS, Simonsen M. Effect of drugs on the risk of injuries in children with attention deficit hyperactivity disorder: A prospective cohort study. *The Lancet Psychiatry*. 2015;2(8):702-709.
240. Raman SR, Marshall SW, Haynes K, Gaynes BN, Naftel AJ, Sturmer T. Stimulant treatment and injury among children with attention deficit hyperactivity disorder: An application of the self-controlled case series study design. *Injury Prevention*. 2013;19(3):164-170.
241. Ruiz-Goikoetxea M, Cortese S, Aznarez-Sanado M, et al. Risk of unintentional injuries in children and adolescents with ADHD and the impact of ADHD medications: A systematic review and meta-analysis. *Neuroscience and Biobehavioral Reviews*. 2018;84:63-71.
242. Perry BA, Archer KR, Song Y, et al. Medication therapy for attention deficit/hyperactivity disorder is associated with lower risk of fracture: a retrospective cohort study. *Osteoporosis International*. 2016;27(7):2223-2227.

©2021 Kazda L et al. *JAMA Network Open*

243. Shem-Tov S, Chodick G, Weitzman D, Koren G. The Association Between Attention-Deficit Hyperactivity Disorder, Injuries, and Methylphenidate. *Glob*. 2019;6:2333794X19845920.
244. Mikolajczyk R, Horn J, Schmedt N, Langner I, Lindemann C, Garbe E. Injury prevention by medication among children with attention-deficit/Hyperactivity disorder a case-only study. *JAMA Pediatrics*. 2015;169(4):391-395.
245. Winterstein AG, Li Y, Gerhard T, Linden S, Shuster JJ. Medication Use for ADHD and the Risk of Driving Citations and Crashes Among Teenage Drivers: A Population-Based Cohort Study. *Journal of Attention Disorders*. 2020:1087054720915768.
246. Levine M, Froberg B, Ruha AM, et al. Assessing the toxicity and associated costs among pediatric patients admitted with unintentional poisonings of attention-deficit/hyperactivity disorder drugs in the United States. *Clinical Toxicology*. 2013;51(3):147-150.
247. Vitiello B, Elliott GR, Swanson JM, et al. Blood pressure and heart rate over 10 years in the multimodal treatment study of children with ADHD. *American Journal of Psychiatry*. 2012;169(2):167-177.
248. Landgren M, Nasic S, Johnson M, Lovoll T, Holmgren D, Fernell E. Blood pressure and anthropometry in children treated with stimulants: A longitudinal cohort study with an individual approach. *Neuropsychiatric Disease and Treatment*. 2017;13:499-506.
249. Cooper WO, Habel LA, Sox CM, et al. ADHD drugs and serious cardiovascular events in children and young adults. *New England Journal of Medicine*. 2011;365(20):1896-1904.
250. Schelleman H, Bilker WB, Strom BL, et al. Cardiovascular events and death in children exposed and unexposed to ADHD agents. *Pediatrics*. 2011;127(6):1102-1110.
251. Winterstein AG, Gerhard T, Kubilis P, et al. Cardiovascular safety of central nervous system stimulants in children and adolescents: Population based cohort study. *BMJ (Online)*. 2012;345 (7869) (no pagination)(e4627).
252. Shin JY, Roughead EE, Park BJ, Pratt NL. Cardiovascular safety of methylphenidate among children and young people with attention-deficit/hyperactivity disorder (ADHD): Nationwide self controlled case series study. *BMJ (Online)*. 2016;353 (no pagination)(i2550).
253. Dalsgaard S, Kvist AP, Leckman JF, Nielsen HS, Simonsen M. Cardiovascular safety of stimulants in children with attention-deficit/hyperactivity disorder: a nationwide prospective cohort study. *Journal of Child and Adolescent Psychopharmacology*. 2014;24(6):302-310.
254. Westover AN, Halm EA. Do prescription stimulants increase the risk of adverse cardiovascular events?: A systematic review. *BMC Cardiovascular Disorders*. 2012;12 (no pagination)(41).
255. Catala-Lopez F, Hutton B, Nunez-Beltran A, et al. The pharmacological and non-pharmacological treatment of attention deficit hyperactivity disorder in children and adolescents: A systematic review with network meta-analyses of randomised trials. *PLoS ONE*. 2017;12(7):e0180355.
256. Cheng JY, Chen RY, Ko JS, Ng EM. Efficacy and safety of atomoxetine for attention-deficit/hyperactivity disorder in children and adolescents-meta-analysis and meta-regression analysis. *Psychopharmacology (Berl)*. 2007;194(2):197-209.
257. Connor DF. Preschool attention deficit hyperactivity disorder: a review of prevalence, diagnosis, neurobiology, and stimulant treatment. *Journal of Developmental and Behavioral Pediatrics*. 2002;23(1 SUPPL.):S1-S9.
258. Cortese S, Adamo N, Del Giovane C, et al. Comparative efficacy and tolerability of medications for attention-deficit hyperactivity disorder in children, adolescents, and adults: a systematic review and network meta-analysis. *The Lancet Psychiatry*. 2018;5(9):727-738.
259. Gayleard JL, Mychailyszyn MP. Atomoxetine treatment for children and adolescents with Attention-Deficit/Hyperactivity Disorder (ADHD): a comprehensive meta-analysis of outcomes on parent-rated core symptomatology. *Atten Defic Hyperact Disord*. 2017;9(3):149-160.
260. Hirota T, Schwartz S, Correll CU. Alpha-2 agonists for attention-deficit/hyperactivity disorder in youth: a systematic review and meta-analysis of monotherapy and add-on trials to stimulant therapy. *J Am Acad Child Psy*. 2014;53(2):153-173.
261. Keen D, Hadijikoumi I. ADHD in children and adolescents. *BMJ Clinical Evidence*. 2011(pagination).
262. King S, Griffin S, Hodges Z, et al. A systematic review and economic model of the effectiveness and cost-effectiveness of methylphenidate, dexamfetamine and atomoxetine for the treatment of attention deficit hyperactivity disorder in children and adolescents. *Health Technology Assessment*. 2006;10(23):iii+146.

©2021 Kazda L et al. *JAMA Network Open*

263. Klassen A, Miller A, Raina P, Lee SK, Olsen L. Attention-deficit hyperactivity disorder in children and youth: a quantitative systematic review of the efficacy of different management strategies. *Canadian Journal of Psychiatry.* 1999;44(10):1007-1016.
264. Maia CR, Cortese S, Caye A, et al. Long-Term Efficacy of Methylphenidate Immediate-Release for the Treatment of Childhood ADHD. *Journal of Attention Disorders.* 2017;21(1):3-13.
265. Maneeton B, Maneeton N, Likhitsathian S, et al. Comparative efficacy, acceptability, and tolerability of lisdexamfetamine in child and adolescent ADHD: a meta-analysis of randomized, controlled trials. *Drug Des Devel Ther.* 2015;9:1927-1936.
266. Moen MD, Keam SJ. Dexmethylphenidate extended release: A review of its use in the treatment of attention-deficit hyperactivity disorder. *CNS Drugs.* 2009;23(12):1057-1083.
267. Otasowie J, Castells X, Ehimare UP, Smith CH. Tricyclic antidepressants for attention deficit hyperactivity disorder (ADHD) in children and adolescents. *Cochrane Database of Systematic Reviews.* 2014(9):CD006997.
268. Parker J, Wales G, Chalhoub N, Harpin V. The long-term outcomes of interventions for the management of attention-deficit hyperactivity disorder in children and adolescents: a systematic review of randomized controlled trials. *Psychology Research and Behavior Management.* 2013;6:87-99.
269. Punja S, Shamseer L, Hartling L, et al. Amphetamines for attention deficit hyperactivity disorder (ADHD) in children and adolescents. *Cochrane Database of Systematic Reviews.* 2016;2:CD009996.
270. Riera M, Castells X, Tobias A, Cunill R, Blanco L, Capella D. Discontinuation of pharmacological treatment of children and adolescents with attention deficit hyperactivity disorder: meta-analysis of 63 studies enrolling 11,788 patients. *Psychopharmacology (Berl).* 2017;234(17):2657-2671.
271. Schachter HM, Pham B, King J, Langford S, Moher D. How efficacious and safe is short-acting methylphenidate for the treatment of attention-deficit disorder in children and adolescents? A meta-analysis. *Canadian Medical Association Journal.* 2001;165(11):1475-1488.
272. Schwartz S, Correll CU. Efficacy and safety of atomoxetine in children and adolescents with attention-deficit/hyperactivity disorder: results from a comprehensive meta-analysis and metaregression. *J Am Acad Child Psy.* 2014;53(2):174-187.
273. Sibley MH, Kuriyan AB, Evans SW, Waxmonsky JG, Smith BH. Pharmacological and psychosocial treatments for adolescents with ADHD: an updated systematic review of the literature. *Clinical Psychology Review.* 2014;34(3):218-232.
274. Storebo OJ, Ramstad E, Krogh HB, et al. Methylphenidate for children and adolescents with attention deficit hyperactivity disorder (ADHD). *Cochrane Database of Systematic Reviews.* 2015;2015 (11) (no pagination)(CD009885).
275. Valdizan-Uson J, Canovas-Martinez A, De Lucas-Taracena M, et al. Response to methylphenidate by adult and pediatric patients with attention-deficit/hyperactivity disorder: The Spanish multicenter DIHANA study. *Neuropsychiatric Disease and Treatment.* 2013;9.
276. Swanson JM, Arnold L, Molina BS, et al. Young adult outcomes in the follow-up of the multimodal treatment study of attention-deficit/hyperactivity disorder: Symptom persistence, source discrepancy, and height suppression. *Journal of Child Psychology and Psychiatry.* 2017;58(6):663-678.
277. van Lieshout M, Luman M, Twisk JW, et al. A 6-year follow-up of a large European cohort of children with attention-deficit/hyperactivity disorder-combined subtype: outcomes in late adolescence and young adulthood. *European Child and Adolescent Psychiatry.* 2016;25(9):1007-1017.
278. Charach A, Ickowicz A, Schachar R. Stimulant treatment over five years: adherence, effectiveness, and adverse effects. *J Am Acad Child Psy.* 2004;43(5):559-567.
279. Bowling A, Davison K, Haneuse S, Beardslee W, Miller DP. ADHD Medication, Dietary Patterns, Physical Activity, and BMI in Children: A Longitudinal Analysis of the ECLS-K Study. *Obesity.* 2017;25(10):1802-1808.
280. Butte NF, Treuth MS, Voigt RG, Llorente AM, Heird WC. Stimulant medications decrease energy expenditure and physical activity in children with attention-deficit/hyperactivity disorder. *Journal of Pediatrics.* 1999;135(2 I):203-207.

©2021 Kazda L et al. *JAMA Network Open*

281. Poulton AS, Bui Q, Melzer E, Evans R. Stimulant medication effects on growth and bone age in children with attention-deficit/hyperactivity disorder: a prospective cohort study. *Int Clin Psychopharmacol.* 2016;31(2):93-99.
282. Zhang H, Du M, Zhuang S. Impact of long-term treatment of methylphenidate on height and weight of school age children with ADHD. *Neuropediatrics.* 2010;41(2):55-59.
283. Diez-Suarez A, Vallejo-Valdivielso M, Marin-Mendez JJ, De Castro-Manglano P, Soutullo CA. Weight, Height, and Body Mass Index in Patients with Attention-Deficit/Hyperactivity Disorder Treated with Methylphenidate. *Journal of Child and Adolescent Psychopharmacology.* 2017;27(8):723-730.
284. Faraone SV, Biederman J, Morley CP, Spencer TJ. Effect of stimulants on height and weight: A review of the literature. *J Am Acad Child Psy.* 2008;47(9):994-1009.
285. Lisska MC, Rivkees SA. Daily methylphenidate use slows the growth of children: A community based study. *Journal of Pediatric Endocrinology and Metabolism.* 2003;16(5):711-718.
286. Poulton A. Growth on stimulant medication; clarifying the confusion: A review. *Archives of Disease in Childhood.* 2005;90(8):801-806.
287. Powell SG, Frydenberg M, Thomsen PH. The effects of long-term medication on growth in children and adolescents with ADHD: An observational study of a large cohort of real-life patients. *Child and Adolescent Psychiatry and Mental Health.* 2015;9 (1) (no pagination)(50).
288. Zachor DA, Roberts AW, Bart Hodgens J, Isaacs JS, Merrick J. Effects of long-term psychostimulant medication on growth of children with ADHD. *Research in Developmental Disabilities.* 2006;27(2):162-174.
289. Harstad EB, Weaver AL, Katusic SK, et al. ADHD, stimulant treatment, and growth: a longitudinal study. *Pediatrics.* 2014;134(4):e935-944.
290. Moran LV, Ongur D, Hsu J, Castro VM, Perlis RH, Schneeweiss S. Psychosis with methylphenidate or amphetamine in patients with ADHD. *New England Journal of Medicine.* 2019;380(12):1128-1138.
291. Angold A, Erkanli A, Egger HL, Costello EJ. Stimulant treatment for children: a community perspective. *J Am Acad Child Psy.* 2000;39(8):975-984; discussion 984-994.
292. Barkley RA, Fischer M, Smallish L, Fletcher K. Does the treatment of attention-deficit/hyperactivity disorder with stimulants contribute to drug use/abuse? A 13-year prospective study. *Pediatrics.* 2003;111(1):97-109.
293. Biederman J, Monuteaux MC, Spencer T, Wilens TE, Macpherson HA, Faraone SV. Stimulant therapy and risk for subsequent substance use disorders in male adults with ADHD: a naturalistic controlled 10-year follow-up study. *American Journal of Psychiatry.* 2008;165(5):597-603.
294. Humphreys KL, Eng T, Lee SS. Stimulant medication and substance use outcomes ameta-analysis. *JAMA Psychiatry.* 2013;70(7):740-749.
295. Huss M, Poustka F, Lehmkuhl G, Lehmkuhl U. No increase in long-term risk for nicotine use disorders after treatment with methylphenidate in children with attention-deficit/hyperactivity disorder (ADHD): Evidence from a non-randomised retrospective study. *Journal of Neural Transmission.* 2008;115(2):335-339.
296. Biederman J, Wilens T, Mick E, Spencer T, Faraone SV. Pharmacotherapy of attention-deficit/hyperactivity disorder reduces risk for substance use disorder. *Pediatrics.* 1999;104(2):e20.
297. Groenman AP, Oosterlaan J, Rommelse NN, et al. Stimulant treatment for attention-deficit hyperactivity disorder and risk of developing substance use disorder. *Br J Psychiatry.* 2013;203(2):112-119.
298. Mannuzza S, Klein RG, Truong NL, et al. Age of methylphenidate treatment initiation in children with ADHD and later substance abuse: Prospective follow-up into adulthood. *American Journal of Psychiatry.* 2008;165(5):604-609.
299. Wilens TE, Adamson J, Monuteaux MC, et al. Effect of prior stimulant treatment for attention-deficit/hyperactivity disorder on subsequent risk for cigarette smoking and alcohol and drug use disorders in adolescents. *Archives of Pediatrics and Adolescent Medicine.* 2008;162(10):916-921.
300. Wilens TE, Faraone SV, Biederman J, Gunawardene S. Does stimulant therapy of attention-deficit/hyperactivity disorder beget later substance abuse? A meta-analytic review of the literature. *Pediatrics.* 2003;111(1):179-185.

301. Bjerkeli PJ, Vicente RP, Mulinari S, Johnell K, Merlo J. Overuse of methylphenidate: An analysis of swedish pharmacy dispensing data. *Clinical Epidemiology*. 2018;10:1657-1665.
302. Wilens TE, Adler LA, Adams J, et al. Misuse and diversion of stimulants prescribed for ADHD: a systematic review of the literature. *J Am Acad Child Psy*. 2008;47(1):21-31.
303. Setlik J, Bond G, Ho M. Adolescent prescription ADHD medication abuse is rising along with prescriptions for these medications. *Pediatrics*. 2009;124(3):875-880.
304. Davies M, Coughtrie A, Layton D, Shakir SA. Use of atomoxetine and suicidal ideation in children and adolescents: Results of an observational cohort study within general practice in England. *European Psychiatry*. 2017;39:11-16.
305. Man KKC, Coghill D, Chan EW, et al. Association of Risk of Suicide Attempts With Methylphenidate Treatment. *JAMA Psychiatry*. 2017;74(10):1048-1055.
306. Chang Z, Quinn PD, O'Reilly L, et al. Medication for Attention-Deficit/Hyperactivity Disorder and Risk for Suicide Attempts. *Biol Psychiatry*. 2019;13:13.
307. Chen Q, Sjolander A, Runeson B, D'Onofrio BM, Lichtenstein P, Larsson H. Drug treatment for attention-deficit/hyperactivity disorder and suicidal behaviour: Register based study. *BMJ (Online)*. 2014;348 (no pagination)(g3769).
308. Liang SH, Yang YH, Kuo TY, et al. Suicide risk reduction in youths with attention-deficit/hyperactivity disorder prescribed methylphenidate: A Taiwan nationwide population-based cohort study. *Research in Developmental Disabilities*. 2018;72:96-105.
309. McCarthy S, Cranswick N, Potts L, Taylor E, Wong IC. Mortality associated with attention-deficit hyperactivity disorder (ADHD) drug treatment: a retrospective cohort study of children, adolescents and young adults using the general practice research database. *Drug Saf*. 2009;32(11):1089-1096.
310. Stuckelman ZD, Mulqueen JM, Ferracioli-Oda E, et al. Risk of irritability with psychostimulant treatment in children with ADHD: A meta-analysis. *Journal of Clinical Psychiatry*. 2017;78(6):e648-e655.
311. Pozzi M, Carnovale C, Peeters G, et al. Adverse drug events related to mood and emotion in paediatric patients treated for ADHD: A meta-analysis. *J Affect Disord*. 2018;238:161-178.
312. Mohr-Jensen C, Bisgaard CM, Boldsen SK, Steinhausen H-C. Attention-deficit/hyperactivity disorder in childhood and adolescence and the risk of crime in young adulthood in a Danish nationwide study. *J Am Acad Child Psy*. 2019;58(4):443-452.
313. Coghill DR, Banaschewski T, Soutullo C, Cottingham MG, Zuddas A. Systematic review of quality of life and functional outcomes in randomized placebo-controlled studies of medications for attention-deficit/hyperactivity disorder. *European Child and Adolescent Psychiatry*. 2017;26(11):1283-1307.
314. Bastiaens L. Improvement in global psychopathology increases quality of life during treatment of ADHD with atomoxetine or stimulants. *Psychiatric Quarterly*. 2011;82(4):303-308.
315. Coghill D. The impact of medications on quality of life in attention-deficit hyperactivity disorder: a systematic review. *CNS Drugs*. 2010;24(10):843-866.
316. Goetz M, Yeh CB, Ondrejka I, et al. A 12-month prospective, observational study of treatment regimen and quality of life associated with adhd in central and eastern europe and eastern asia. *Journal of Attention Disorders*. 2012;16(1):44-59.
317. Benner-Davis S, Heaton PC. Attention deficit and hyperactivity disorder: controversies of diagnosis and safety of pharmacological and nonpharmacological treatment. *Curr Drug Saf*. 2007;2(1):33-42.
318. Ching C, Eslick GD, Poulton AS. Evaluation of Methylphenidate Safety and Maximum-Dose Titration Rationale in Attention-Deficit/Hyperactivity Disorder: A Meta-analysis. *JAMA Pediatrics*. 2019.
319. Clavenna A, Bonati M. Safety of medicines used for ADHD in children: a review of published prospective clinical trials. *Archives of Disease in Childhood*. 2014;99(9):866-872.
320. Didoni A, Sequi M, Panei P, Bonati M. One-year prospective follow-up of pharmacological treatment in children with attention-deficit/hyperactivity disorder. *European Journal of Clinical Pharmacology*. 2011;67(10):1061-1067.
321. Holmskov M, Storebo OJ, Moreira-Maia CR, et al. Gastrointestinal adverse events during methylphenidate treatment of children and adolescents with attention deficit hyperactivity disorder: A systematic review with meta-analysis and Trial Sequential Analysis of randomised clinical trials. *PLoS ONE*. 2017;12(6):e0178187.

©2021 Kazda L et al. *JAMA Network Open*

322. Safer DJ. Age-grouped differences in adverse drug events from psychotropic medication. *Journal of Child and Adolescent Psychopharmacology.* 2011;21(4):299-309.
323. Ghanizadeh A, Freeman RD, Berk M. Efficacy and adverse effects of venlafaxine in children and adolescents with ADHD: a systematic review of non-controlled and controlled trials. *Rev Recent Clin Trials.* 2013;8(1):2-8.
324. Konrad-Bindl DS, Gresser U, Richartz BM. Changes in behavior as side effects in methylphenidate treatment: Review of the literature. *Neuropsychiatric Disease and Treatment.* 2016;12:2635-2647.
325. Storebo OJ, Pedersen N, Ramstad E, et al. Methylphenidate for attention deficit hyperactivity disorder (ADHD) in children and adolescents - assessment of adverse events in non-randomised studies. *Cochrane Database of Systematic Reviews.* 2018;2018 (5) (no pagination)(CD012069).
326. Frank E, Ozon C, Nair V, Othee K. Examining why patients with attention-deficit/hyperactivity disorder lack adherence to medication over the long term: a review and analysis. *Journal of Clinical Psychiatry.* 2015;76(11):e1459-1468.
327. Gajria K, Lu M, Sikirica V, et al. Adherence, persistence, and medication discontinuation in patients with attention-deficit/hyperactivity disorder - A systematic literature review. *Neuropsychiatric Disease and Treatment.* 2014;10:1543-1569.
328. Arnold LE, Hodgkins P, Caci H, Kahle J, Young S. Effect of treatment modality on long-term outcomes in attention-deficit/hyperactivity disorder: a systematic review. *PLoS One.* 2015;10(2):e0116407.
329. Chang Z, Ghirardi L, Quinn PD, Asherson P, D'Onofrio BM, Larsson H. Risks and Benefits of Attention-Deficit/Hyperactivity Disorder Medication on Behavioral and Neuropsychiatric Outcomes: A Qualitative Review of Pharmacoepidemiology Studies Using Linked Prescription Databases. *Biol Psychiatry.* 2019.
330. Boland H, DiSalvo M, Fried R, et al. A literature review and meta-analysis on the effects of ADHD medications on functional outcomes. *J Psychiatr Res.* 2020;123:21-30.
331. Schachar R, Jadad AR, Gauld M, et al. Attention-deficit hyperactivity disorder: critical appraisal of extended treatment studies. *Canadian Journal of Psychiatry.* 2002;47(4):337-348.
332. Ercan ES, Kose S, Kutlu A, Akyol O, Durak S, Aydin C. Treatment duration is associated with functioning and prognosis in children with attention deficit hyperactivity disorder. *Klinik Psikofarmakoloji Bulteni.* 2012;22(2):148-160.
333. Smith G, Jongeling B, Hartmann P, Russell C, Landau L. *Raine ADHD Study: Long-term outcomes associated with stimulant medication in the treatment of ADHD in children.* Western Australian Department of Health;2010.
334. Molina BS, Hinshaw SP, Swanson JM, et al. The MTA at 8 years: prospective follow-up of children treated for combined-type ADHD in a multisite study. *J Am Acad Child Psy.* 2009;48(5):484-500.
335. M. T. A. Cooperative Group. National Institute of Mental Health Multimodal Treatment Study of ADHD follow-up: changes in effectiveness and growth after the end of treatment. *Pediatrics.* 2004;113(4):762-769.
